# Nucleophilic functionalization of thianthrenium salts under basic conditions

**DOI:** 10.3762/bjoc.20.26

**Published:** 2024-02-08

**Authors:** Xinting Fan, Duo Zhang, Xiangchuan Xiu, Bin Xu, Yu Yuan, Feng Chen, Pan Gao

**Affiliations:** 1 School of Chemistry and Chemical Engineering, Yangzhou University, Yangzhou 225002, China; 2 Medicine Center, Guangxi University of Science and Technology, Liushi Road 257, Liuzhou, Guangxi 545006, China

**Keywords:** amination, functionalization of alcohol, metal-free, S-(alkyl)thianthrenium salts, thioetherification

## Abstract

In recent years, S-(alkyl)thianthrenium salts have become an important means of functionalizing alcohol compounds. However, additional transition metal catalysts and/or visible light are required. Herein, a direct thioetherification/amination reaction of thianthrenium salts is realized under metal-free conditions. This strategy exhibits good functional-group tolerance, operational simplicity, and an extensive range of compatible substrates.

## Introduction

**Scheme 1:**
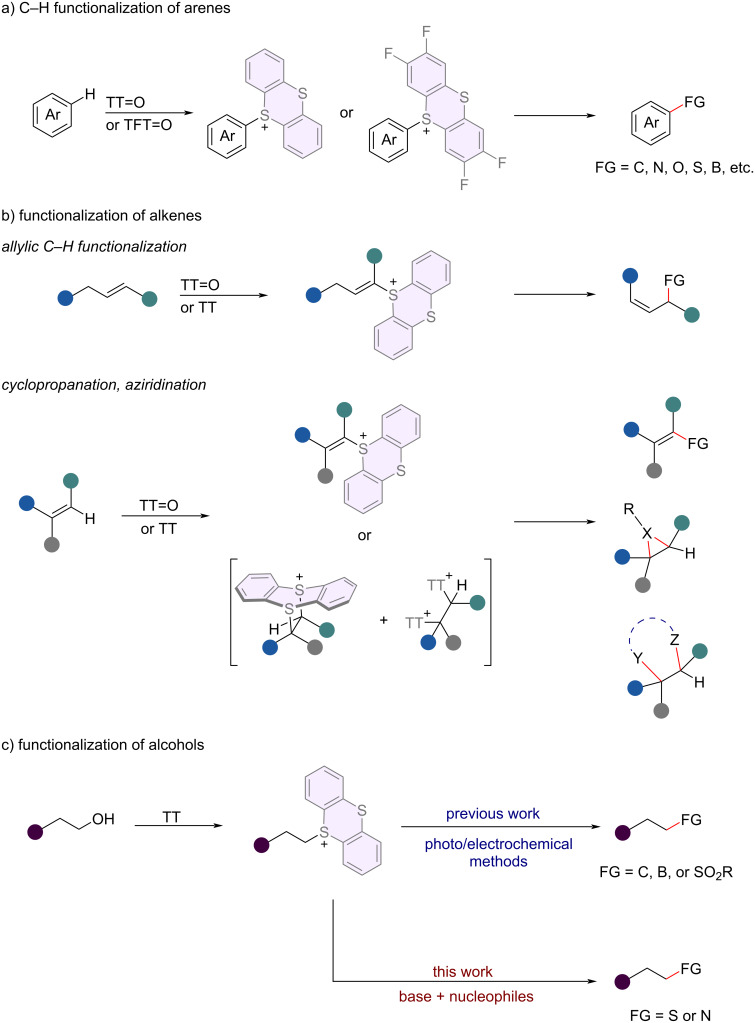
Synthetic application of thianthrenium salts.

Sulfonium salts [1–10] have been extensively utilized as readily accessible synthetic building blocks in organic synthesis, particularly in the *ipso*-functionalization of C–S bonds. Of the sulfonium salts, organothianthrenium salts exhibit distinct structural properties and reactivities, thereby offering further potential in organic synthesis. Despite establishing a few preliminary methods for preparing organothianthrenium salts, their application potential is historically thwarted by harsh synthetic conditions and the poor durability of the resulting products [7]. Recently, Ritter and co-workers have introduced a pioneering method for synthesizing air- and moisture-stable arylthianthrenium salts [11]. This novel approach involves the utilization of *tetra*-fluorothianthrene sulfoxide (TFT=O) or thianthrene sulfoxide (TT=O), which react with arenes under mild conditions, exhibiting exclusive regioselectivity. Significant advancements in the synthesis of arylthianthrenium salts have prompted a growing interest in their utilization as versatile precursors for the conversion of C–H bonds in arenes into C–C/X bonds through transition-metal-catalyzed cross-coupling processes [12–20]. On the other hand, sulfonium salts have emerged as appealing sources of aryl radicals for a wide range of transformations aimed at creating novel chemical bonds driven by their distinctive structural attributes and chemical tendencies (Scheme 1a) [9,21–26]. In addition to late-stage C–H functionalization of arenes, Wickens’s group has introduced an oxidative alkene aziridination strategy that relies on thianthrenation of an alkene under electrochemical conditions [27]. Subsequently, cyclopropanation, [28] aziridination, [29] allylic C–H functionalization, [30–31] transition-metal-catalyzed cross-coupling [32–33] and aminofunctionalization [34] of alkenes were achieved, benefiting from the unique reactivity of organothianthrenium species that are generated through the reaction of alkenes and thianthrene sulfoxide (TT=O) or thianthrene (TT) (Scheme 1b).

Alcohols are widely accessible and have significant importance in the pharmaceutical industry, positioning them as appealing candidates for C(sp^3^) coupling due to their availability as a common chemical feedstock. However, due to the high bond dissociation energy of the C–O bond and the poor leaving ability of the hydroxy group [35], it is still a great challenge to transform alcohols into valuable chemicals [36–38]. A recent study by Shi and co-workers has successfully converted alcohols into thianthrenium salts, enabling the transformation of the hydroxy (OH) group into various functional groups via the photoassisted generation of alkyl radicals [39]. After that, a series of methods for the modification of alkylthianthrenium salts have been developed, including the transition-metal-catalyzed cross-coupling with terminal alkynes [40], sulfonylation with DABCO·(SO_2_)_2_ [41–43], or alkylation of active alkenes [44–45]. Recently, Ritter and co-workers reported that alkylthianthrenium salts can be employed to undergo reactions with N/O-nucleophiles under photocatalytic conditions [46]. Nevertheless, additional transition metal catalysts, visible light, or electrochemical devices are required for the reported works. Therefore, developing a green method to functionalize alkylthianthrenium salts is still highly desirable. Considering the highly polarized C(sp^3^)–S bond in alkylthianthrenium salts, alkylthianthrenium salts have the potential to serve as alkyl electrophiles to react with nucleophiles directly in the absence of a metal catalyst [46].

## Results and Discussion

With these considerations in mind, we investigated the possibility of the thioetherification between alkylthianthrenium salts and thiophenols. After extensive screening of the reaction parameters, the desired thioetherification product **3aa** was obtained in 88% yield under the following optimal conditions: S-(alkyl)thianthrenium salt **1a** and 4-bromothiophenol (**2a**) as the model substrates, *N*,*N*-diisopropylethylamine (DIPEA) as the base, were added to a vessel in air; after stirring for 16 hours at room temperature, the corresponding product was isolated by chromatographic purification in 88% yield (Table 1, entry 1). Reducing the amount of DIPEA gave diminished yield (Table 1, entry 2). Adding more base was unnecessary to get a higher yield (Table 1, entry 3). Subsequently, the use of other bases was not appropriate for promoting the generation of the thioetherification product **3aa** (Table 1, entries 4–7). Furthermore, the yields significantly varied (63−79%) depending on the different solvents tested (Table 1, entries 8–11).

**Table 1:** Optimization of the thioetherification of S-(alkyl)thianthrenium salt.^a^

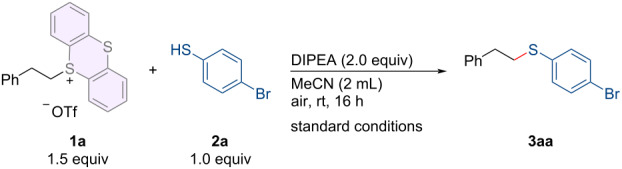		

Entry	Deviation from "standard conditions"	Yield of **2a** (%)^b^

1	none	88
2	1.5 equiv of DIPEA	73
3	3.0 equiv of DIPEA	85
4	NEt_3_ instead of DIPEA	76
5	Na_2_CO_3_ instead of DIPEA	74
6	LiO*t*-Bu instead of DIPEA	67
7	NaOH instead of DIPEA	71
8	toluene as solvent	63
9	DMF as solvent	67
10	THF as solvent	72
11	DCM as solvent	79

^a^Reaction conditions: all reactions were carried out using **1a** (0.3 mmol), **2a** (0.2 mmol), and DIPEA (0.4 mmol) in 2.0 mL of MeCN at room temperature for 16 h under an air atmosphere. ^b^Isolated yields after purification by column chromatography.

**Scheme 2:**
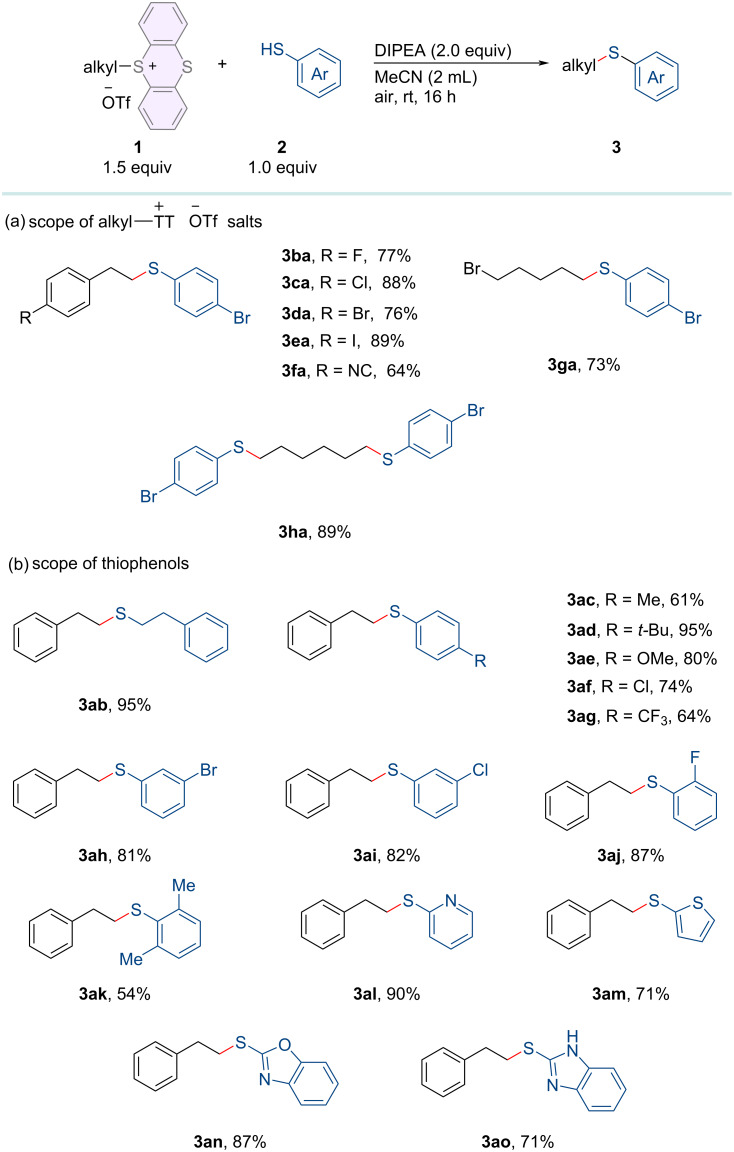
Substrate scope. Reaction conditions: alkylthianthrenium salts **1** (0.3 mmol), thiophenols **2** (0.2 mmol), DIPEA (0.4 mmol) in 2.0 mL of MeCN at room temperature for 16 h under air atmosphere. Isolated yields.

Having established the optimized reaction conditions, we assessed the range of substrates suitable for this method. First, the scope of sulfonium salts was examined, as summarized in Scheme 2a. Alkylsulfonium salts substituted with a halide (F, Cl, or Br) or isocyano group at the *para*-position of the aryl ring (**1b–e**) were successfully converted into the carbon–sulfur bond formation products (**3ba–fa**) in moderate to good yields. Even when sulfonium salt **1g** bearing a C(sp^3^)–Br bond is susceptible to nucleophilic attack, the desired product **3ga** can still be obtained in good yield. Furthermore, substrate **1h** featuring two sulfonium salt motifs could undergo dual thioetherification at both reaction sites, resulting in the target product **3ha** in good yield. Next, we investigated the compatibility of various thiophenols with thianthrenium salt **1a** (Scheme 2b). When simple thiophenol **2b** was used as the substrate, a good yield of the target product **3ab** was obtained smoothly. To our satisfaction, both electron-donating groups (Me, *t*-Bu, OMe; **2c–e**) and electron-withdrawing groups (Cl and CF_3_; **2f**, and **2g**) at the *para*-position of the aryl ring of thiophenols were well tolerated, furnishing the desired products (**3ac–ag**) in good yields. The reaction yield remains unaffected by the position of halogen substituents (**2h–j**), and the resulting products (**3ah–aj**) can also be obtained with high efficiency. This underscores the viability of integrating this metal-free thioetherification method with other traditional cross-coupling reactions. Sterically hindered *ortho*-disubstituted thiophenol **2k** is also compatible with this reaction system, yielding product **3ak** in good yield. Furthermore, heteroaromatic rings, such as pyridine (**2l**), thiophene (**2m**), benzoxazole (**2n**), and benzimidazole (**2o**) all afforded the desired products (**3al–ao**) in satisfactory yields.

**Scheme 3:**
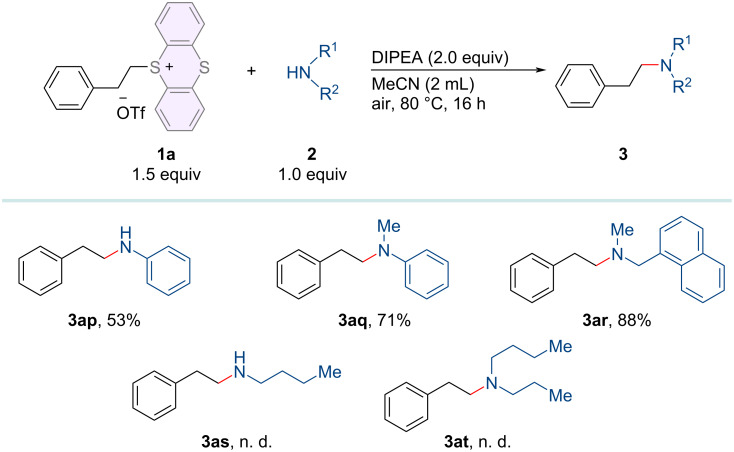
Substrate scope of amines. Reaction conditions: alkylthianthrenium salts **1** (0.3 mmol), amines **2** (0.2 mmol), DIPEA (0.4 mmol) in 2.0 mL of MeCN at 80 °C for 16 h under air atmosphere. Isolated yields.

Subsequently, we investigated the substrate scope of amines, which is outlined in Scheme 3. To our delight, various amines (**2p–r**), including aniline, *N*-methylaniline, and naphthylmethylamine are also compatible under the optimal conditions to give the corresponding amination products (**3ap–ar**) in moderate to high yields. For this amination method, it was necessary to investigate simple alkylamines (**2s** and **2t**) as the substrates. In doing so, we could not isolate the corresponding amination products **3as** and **3at**.

**Scheme 4:**
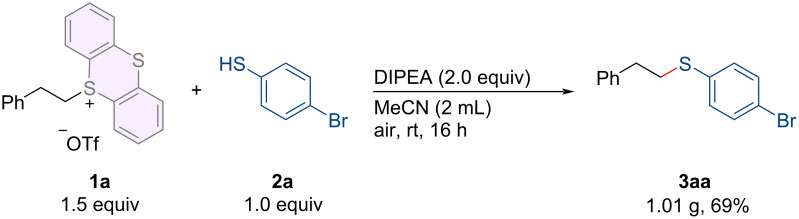
Scale-up reaction.

To showcase the practical utility of our metal-free thioether formation process, we conducted a 5.0 mmol scale reaction and obtained the target product **3aa** in 69% yield (Scheme 4). This operationally simple protocol enables the rapid development of novel thioetherification reactions using bench-stable alkylthianthrenium salts as the electrophiles. As is well known, alkyl trifluoromethanesulfonate (alkyl-OTf), serving as a potent electrophilic reagent, can also engage in reactions with electrophilic reagents like thiophenol or amines under alkaline conditions, facilitating the formation of respective C–N/O bonds. The synthesis of alkylthianthrenium salts requires alkyl trifluoromethanesulfonate as a precursor, which can also act as an electrophile. However, alkyl-OTf is prone to decomposition and poses challenges for storage at ambient temperature (for details, see Supporting Information File 1).

## Conclusion

In summary, using the presented operational simple and metal-free method, we have synthesized thioethers and amines from bench stable and readily available alkylthianthrenium salts. Given the importance of alkylthianthrenium salts in synthetic chemistry as alkyl reagents and the distinctive reactivities observed under photo/electrochemical conditions, we foresee significant opportunities emerging in the functionalization of alkylthianthrenium salts using nucleophiles directly, without the need for an external metal catalyst.

## Supporting Information

File 1 Experimental procedures, characterization data for all new compounds, and NMR spectra of products.

## Data Availability

All data that supports the findings of this study is available in the published article and/or the supporting information to this article.
