# Supplementary material for: Nucleophilic functionalization of thianthrenium salts under basic conditions
Source: Beilstein J Org Chem. 2024 Feb 8;20:257–63. doi: 10.3762/bjoc.20.26 (PMC10862136; doi:10.3762/bjoc.20.26)
Supplement: File 1 — Experimental procedures, characterization data for all new compounds, and NMR spectra of products. [file Beilstein_J_Org_Chem-20-257-s001.pdf]

BEILSTEIN JOURNAL OF ORGANIC CHEMISTRY

Supporting Information

for

Nucleophilic functionalization of thianthrenium salts under basic conditions

Xinting Fan, Duo Zhang, Xiangchuan Xiu, Bin Xu, Yu Yuan, Feng Chen and Pan Gao

Beilstein J. Org. Chem. **2024,** *20,* 257–263. doi:10.3762/bjoc.20.26

Experimental procedures, characterization data for all new compounds, and NMR spectra of products

License and Terms: This is a supporting information file under the terms of the Creative Commons Attribution License (https://creativecommons.org/licenses/by/4.0). Please note that the reuse, redistribution and reproduction in particular requires that the author(s) and source are credited and that individual graphics may be subject to special legal provisions.
The license is subject to the *Beilstein Journal of Organic Chemistry* terms and conditions: (https://www.beilstein-journals.org/bjoc/terms)

Table of contents

1. General information. S1

2. General procedure for the synthesis of starting materials..S2

3. Experimental procedures S5

4. Mechanistic studies..S18

5. References...S19

6. Spectra of ^{1}H NMR, ^{13}C NMR, ^{19}F NMR S20

1. General information.

All new compounds were fully characterized. All reactions and manipulations involving air-sensitive compounds were performed using standard Schlenk techniques. Anhydrous MeCN was purchased from Annaiji Chemical and was used as received. ^{1}H and ^{13}C NMR spectra were recorded on an Agilent DD2 400 MHz spectrometer. The chemical shifts in ^{1}H NMR spectra were recorded relative to $CDCl_3$ (δ 7.26). The chemical shifts in ^{13}C NMR spectra were recorded relative to $CDCl_3$ (δ 77.0). The high-resolution mass spectral (HRMS) data were obtained on quadrupole-type Bruker Dalton MAXIS (APCI). Gas analyses were conducted with a Shimadzu GC-2014 equipped with ULBON HR-1 glass capillary column (Shinwa Chemical Industries) and an FID detector. Unless otherwise noted, materials obtained from commercial suppliers were used without further purification.

2. General procedure for the synthesis of starting materials

General procedure A

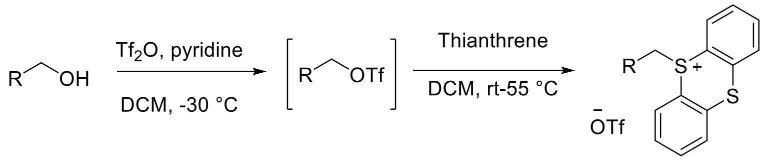

A flame-dried 100 mL flask was placed under an atmosphere of nitrogen and charged with a stir bar and alcohol (5.0 mmol, 1.0 equiv). The alcohol was dissolved in CH_2Cl_2 (20.0 mL) and cooled to −30 °C before adding pyridine (483 µL, 6.0 mmol, 1.2 equiv). While stirring, triflic anhydride (1.0 mL, 29.3 mmol, 1.20 equiv) was added dropwise, and then the reaction mixture stirred for 3 h while remaining at −5 °C. While the flask was still in a −5 °C bath, 0.5 M H_2SO_4 (30 mL) was added. The flask was removed from the cold bath, and the mixture was transferred to a separatory funnel and extracted with 3 × 20 mL of CH_2Cl_2. The organic layers were combined and washed 1 × 50 mL of distilled water. The collected organic layers were then dried over $MgSO_4$, then filtered and concentrated to a 10 mL liquid under vacuum (without heating), which was used directly in the next step.

Flame-dried 25 mL Schlenk tube was added thianthrene (1.08 g, 5.0 mmol), then the above liquid was added. The mixture was stired at 55 °C for 24 h. The mixture was carefully condensed under reduced pressure at 25 ºC and purified by precipitation with Et_2O/DCM. Most of the unreacted dibenzothiophene was removed by repeating the precipitation procedure 2 or 3 times. If the salt still did not precipitate, it was sub*j*ected to silica gel chromatography with acetone/DCM.[1]

Method B:

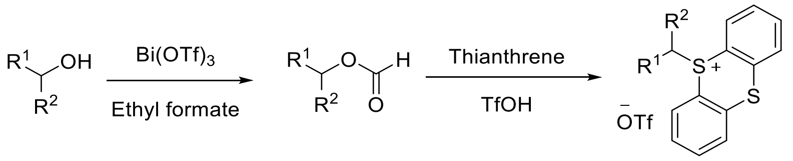

A solution of alcohol (20 mmol) in ethyl formate (60 mL) was treated with 0.4 mmol of $Bi(OTf)_3$. The reaction mixture was stirred under reflux conditions for 5 h. Evaporation of the solvent followed by silica-gel chromatography gave the pure formate.

To a stirred mixture of thianthrene (1.08 g, 5.0 mmol) and formate (10.0 mmol), cooled in an ice-bath, was added 2.5 mL of trifluoromethanesulfonic acid. The mixture was removed from the ice-bath and stirred for 10 h at room temperature, after which it was poured into 100 mL of water. The resulting suspension was extracted with DCM. The collected organic layers were then dried over $MgSO_4$, then filtered and concentrated under reduced pressure at 25 ºC and purified by precipitation with Et_2O/DCM. Most of the unreacted dibenzothiophene was removed by repeating the precipitation procedure 2 or 3 times.[1]

Alkylthianthrenium salts exhibit greater stability and are more amenable to long-term preservation when compared to alkyl trifluoromethanesulfonate compounds. For instance, when phenethyl trifluoromethanesulfonate is exposed to air at room temperature for 24 hours, its initially colorless and transparent appearance transforms into a deep yellow hue (Scheme S1). Our comparison of the NMR data reveals that compound has undergone decomposition, resulting in the formation of unidentified by-products. By comparison, alkylthianthrenium salt **1a** do not exhibit significant changes

after being placed in the air for 24 hours (Scheme S3).

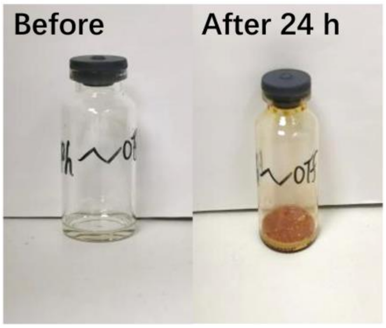

Scheme S1

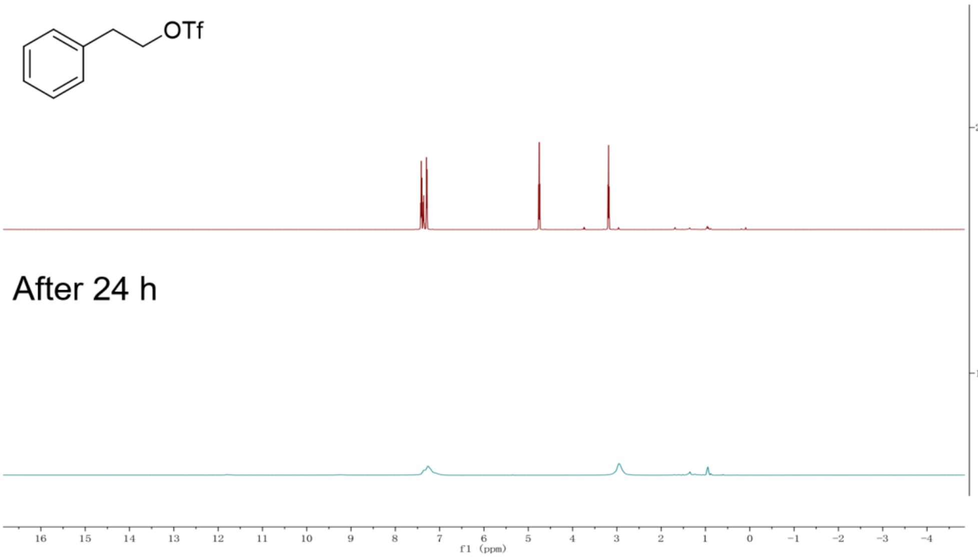

Scheme S2

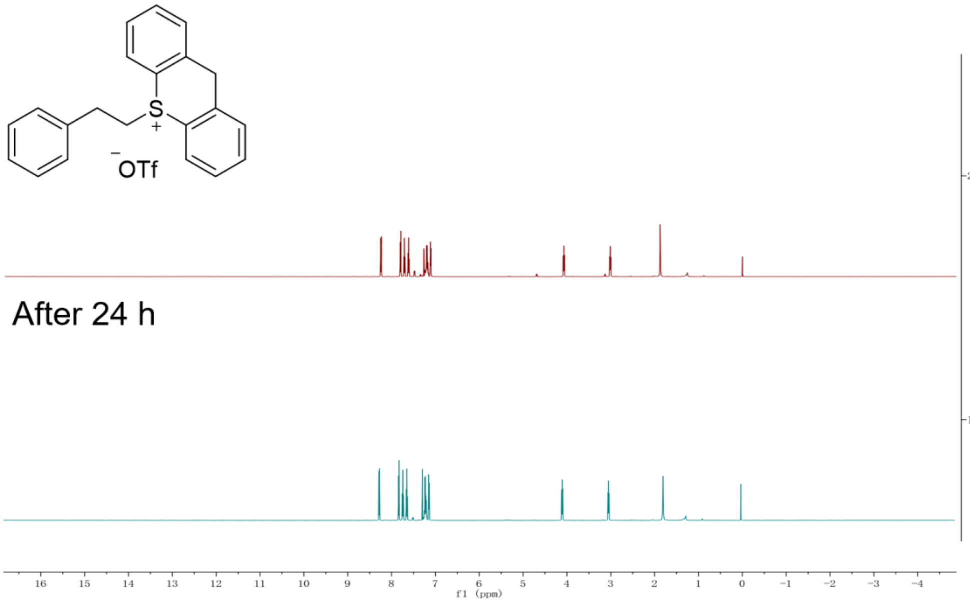

Scheme S3

3. Experimental procedures

General procedure for the synthesis of thioetherification products:

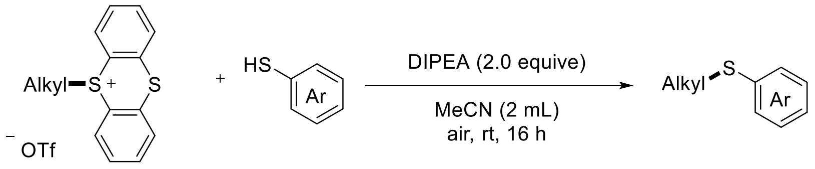

1 **2** **3**

To a 10 mL Schlenk tube was added alkyl sulfonate **1** (0.3 mmol,1.5 equiv), thiophenol **2** (0.2 mmol, 1.0 equiv), DIPEA (0.4 mmol, 2.0 equiv) and anhydrous acetonitrile (2 mL). The resulting mixture was stirred at room temperature for 16 h. The reaction mixture was quenched with dichloromethane. The organic layer was concentrated and purified by silica gel chromatography (petroleum ether and ethyl acetate) to afford the desired product **3**.

Gram-scale reaction:

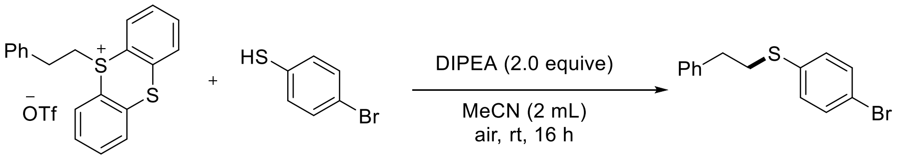

1a 6 mmol; **2a** 5 mmol; **3aa** 1.01 g,69%

To a 50 mL Schlenk tube was added alkyl sulfonate **1a** (6 mmol, 2.82 g, 1.5 equiv), thiophenol **2a** (5 mmol, 0.96 g, 1.0 equiv), DIPEA (10 mmol, 1.77 mL, 2.0 equiv) and anhydrous acetonitrile (10 mL). The resulting mixture was stirred at room temperature for 16 h. After the reaction was completed, it was quenched with water. The residue was then extracted with CH_2Cl_2 (3 × 20 mL) and the combined organic layers were dried over anhydrous Na_2SO_4. The organic layer was concentrated in vacuo, resulting in a Colorless oil. It was then concentrated and purified by silica gel chromatography to afford **3aa** (1.01 g, yield: 69%).

(4-Bromophenyl)(phenethyl)sulfane (3aa)[2]

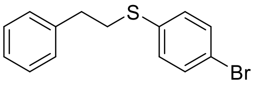

3aa

Following the general procedure, the reaction of **1a** (141.3 mg, 0.3 mmol, 1.5 equiv), **2a** (38.3 mg, 0.2 mmol, 1.0 equiv), DIPEA (71 µL, 0.4 mmol, 2.0 equiv) and anhydrous acetonitrile (2 mL) at room temperature for 16 h. After column chromatography on silica (eluent: petroleum ether), afford **3aa** (55.4 mg, 88%) as a colorless oil. **^{1}H NMR (400 MHz, Chloroform-d)** δ 7.42 (d, *J* = 7.0 Hz, 2H), 7.32 (t, *J* = 7.3 Hz, 2H), 7.27 – 7.16 (m, 5H), 3.16 (t, *J* = 7.8 Hz, 2H), 2.98 – 2.89 (m, 2H) ppm. **^{13}C NMR (101 MHz, Chloroform-d)** δ 139.9, 135.7, 132.0, 130.7, 128.6, 128.5, 126.7, 119.8, 35.5, 35.2 ppm. **HRMS m/z (ESI):** calcd for $C_{14}H_{14}BrS$ $(M + H)^+$ 292.9994, found 292.9996.

(5-Bromophenyl)(4-fluorophenethyl)sulfane (3ba)

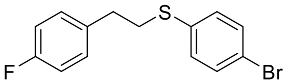

3ba

Following the general procedure, the reaction of **1b** (146.7 mg, 0.3 mmol, 1.5 equiv), **2a** (38.3 mg, 0.2 mmol, 1.0 equiv), DIPEA (71 µL, 0.4 mmol, 2.0 equiv) and anhydrous acetonitrile (2 mL) at room temperature for 16 h. After column chromatography on silica (eluent: petroleum ether), afford **3ba** (47.9 mg, 77%) as a colorless oil. **^{1}H NMR (400 MHz, Chloroform-d)** δ 7.42 (d, *J* = 8.4 Hz, 2H), 7.20 (d, *J* = 8.4 Hz, 2H), 7.17 – 7.10 (m, 2H), 6.99 (t, *J* = 8.6 Hz, 2H), 3.17 – 3.09 (m, 2H), 2.94 – 2.85 (m, 2H) ppm. **^{13}C NMR (101 MHz, Chloroform-d)** δ 161.6 (d, *J* = 244.6 Hz), 135.5 (d, *J* = 3.2 Hz), 135.4, 132.0, 130.8, 129.9 (d, *J* = 7.9 Hz), 119.9, 115.3 (d, *J* = 21.2 Hz), 35.3, 34.6 ppm. **^{19}F NMR (376 MHz, Chloroform-d)** δ -116.42 ppm. **EI-MS (m/z, relative intensity)**: 311.21 (M+, 55), 309.94 (55), 202.94 (64), 200.93 (72), 123.07 (86), 122.03 (100), 109.02 (50), 103.01 (42).

(4-Bromophenyl)(4-chlorophenethyl)sulfane (3ca)[2]

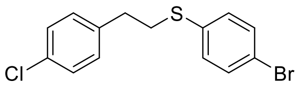

3ca

Following the general procedure, the reaction of **1c** (151.5 mg, 0.3 mmol, 1.5 equiv), **2a** (38.3 mg, 0.2 mmol, 1.0 equiv), DIPEA (71 μL, 0.4 mmol, 2.0 equiv) and anhydrous acetonitrile (2 mL) at room temperature for 16 h. After column chromatography on silica (eluent: petroleum ether), afford **3ca** (57.8 mg, 88%) as a colorless oil. **^{1}H NMR (400 MHz, Chloroform-d)**δ 7.41 (d, J = 8.4 Hz, 2H), 7.26 (d, J = 8.3 Hz, 2H), 7.19 (d, J = 8.4 Hz, 2H), 7.10 (d, J = 8.3 Hz, 2H), 3.16 – 3.08 (m, 2H), 2.92 – 2.83 (m, 2H) ppm. **^{13}C NMR (101 MHz, Chloroform-d)** δ 138.2, 135.3, 132.4, 132.0, 130.9, 129.9, 128.7, 120.0, 35.1, 34.8 ppm. **HRMS m/z (ESI):** calcd for $C_{14}H_{12}BrClNaS$ $(M + Na)^{+}$ 348.9424, found 348.9428.

(4-Bromophenethyl)(4-bromophenyl)sulfane (3da)[3]

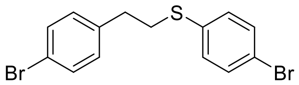

3da

Following the general procedure, the reaction of **1d** (164.7 mg, 0.3 mmol, 1.5 equiv), **2a** (38.3 mg, 0.2 mmol, 1.0 equiv), DIPEA (71 μL, 0.4 mmol, 2.0 equiv) and anhydrous acetonitrile (2 mL) at room temperature for 16 h. After column chromatography on silica (eluent: petroleum ether), afford **3da** (56.3 mg, 76%) as a colorless oil. **^{1}H NMR (400 MHz, Chloroform-d)** δ 7.42 (dd, J = 8.5, 2.1 Hz, 4H), 7.20 (d, J = 8.5 Hz, 2H), 7.05 (d, J = 8.3 Hz, 2H), 3.16 – 3.08 (m, 2H), 2.90 – 2.83 (m, 2H) ppm. **^{13}C NMR (101 MHz, Chloroform-d)** δ 138.8, 135.3, 132.0, 131.6, 130.9, 130.3, 120.4, 120.0, 35.0, 34.8 ppm. **HRMS m/z (APCI):** calcd for $C_{14}H_{12}Br_{2}S$ $(M)^{+}$ 369.9026, found 369.9024.

(5-Bromophenyl)(4-iodophenethyl)sulfane (3ea)

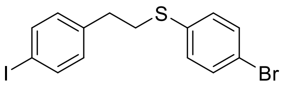

3ea

Following the general procedure, the reaction of **1e** (178.9 mg, 0.3 mmol, 1.5 equiv.), **2a** (38.3 mg, 0.2 mmol, 1.0 equiv), DIPEA (71 µL, 0.4 mmol, 2.0 equiv) and anhydrous acetonitrile (2 mL) at room temperature for 16 h. After column chromatography on silica (eluent: petroleum ether), afford **3ea** (74.5 mg, 89%) as a colorless oil. **^{1}H NMR (400 MHz, Chloroform-d)** δ 7.62 (d, J = 8.2 Hz, 2H), 7.41 (d, J = 8.4 Hz, 2H), 7.19 (d, J = 8.5 Hz, 2H), 6.93 (d, J = 8.1 Hz, 2H), 3.16 – 3.08 (m, 2H), 2.90 – 2.81 (m, 2H) ppm. **^{13}C NMR (101 MHz, Chloroform-d)** δ 139.4, 137.6, 135.3, 132.0, 130.9, 130.6, 120.0, 91.8, 35.0, 34.9 ppm. **HRMS m/z (ESI):** calcd for $C_{14}H_{12}BrINaS$ $(M + Na)^+$ 440.8780, found 440.8718.

4-(2-((4-Bromophenyl)thio)ethyl)benzonitrile (3fa)

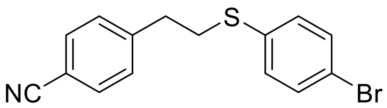

3fa

Following the general procedure, the reaction of **1f** (148.8 mg, 0.3 mmol, 1.5 equiv.), **2a** (38.3 mg, 0.2 mmol, 1.0 equiv), DIPEA (71 µL, 0.4 mmol, 2.0 equiv) and anhydrous acetonitrile (2 mL) at room temperature for 16 h. After column chromatography on silica (eluent: petroleum ether), afford **3fa** (40.4 mg, 64%) as a colorless oil. **^{1}H NMR (400 MHz, Chloroform-d)** δ 7.58 (d, *J* = 8.2 Hz, 2H), 7.41 (d, *J* = 8.5 Hz, 2H), 7.27 (d, *J* = 8.1 Hz, 2H), 7.19 (d, *J* = 8.5 Hz, 2H), 3.14 (t, *J* = 7.5 Hz, 2H), 2.95 (t, *J* = 7.5 Hz, 2H) ppm. **^{13}C NMR (101 MHz, Chloroform-d)** δ 145.2, 134.8, 132.3, 132.1, 131.1, 129.4, 120.3, 118.8, 110.5, 35.4, 34.7 ppm. **HRMS m/z (ESI):** calcd for $C_{15}H_{12}BrNNaS$ $(M + Na)^+$ 339.9766, found 339.9756.

(7-Bromopentyl)(4-bromophenyl)sulfane (3ga)

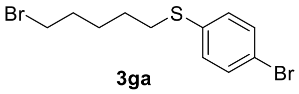

3ga

Following the general procedure, the reaction of **1g** (158.7 mg, 0.3 mmol, 1.5 equiv), **2a** (38.3 mg, 0.2 mmol, 1.0 equiv), DIPEA (71 μL, 0.4 mmol, 2.0 equiv) and anhydrous acetonitrile (2 mL) at room temperature for 16 h. After column chromatography on silica (eluent: petroleum ether), afford **3ga** (49.5 mg, 73%) as a colorless oil. **^{1}H NMR (400 MHz, Chloroform-d)** δ 7.39 (d, J = 8.6 Hz, 2H), 7.18 (d, J = 8.6 Hz, 2H), 3.39 (t, J = 6.7 Hz, 2H), 2.90 (t, J = 7.1 Hz, 2H), 1.86 (p, J = 6.8 Hz, 2H), 1.71 – 1.51 (m, 4H) ppm. **^{13}C NMR (101 MHz, Chloroform-d)** δ 135.8, 131.9, 130.6, 119.6, 33.49, 33.46, 32.2, 28.1, 27.3 ppm. **HRMS m/z (APCI):** calcd for $C_{11}H_{15}Br_2S$ $(M + H)^+$ 336.9256, found 339.9245.

1,6-Bis((4-bromophenyl)thio)hexane (3ha)

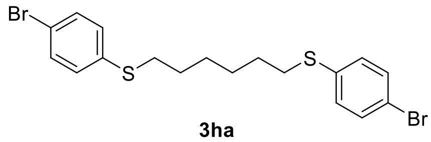

3ha

Following the general procedure, the reaction of **1h** (179.6 mg, 0.3 mmol, 1.5 equiv), **2a** (76.6 mg, 0.4 mmol, 2.0 equiv), DIPEA (71 μL, 0.4 mmol, 2.0 equiv) and anhydrous acetonitrile (2 mL) at room temperature for 16 h. After column chromatography on silica (eluent: petroleum ether), afford **3ha** (124.9 mg, 89%) as a white solid. **^{1}H NMR (400 MHz, Chloroform-d)** δ 7.38 (d, J = 8.5 Hz, 4H), 7.16 (d, J = 8.5 Hz, 4H), 2.87 (t, J = 7.3 Hz, 4H), 1.68 – 1.59 (m, 4H), 1.42 (p, J = 3.7 Hz, 4H) ppm. **^{13}C NMR (101 MHz, Chloroform-d)** δ 136.0, 131.9, 130.4, 119.5, 33.5, 28.8, 28.2 ppm. **m.p.:** 73.6-78.8 °C. **HRMS m/z (ESI):** calcd for $C_{18}H_{21}Br_2S_2$ $(M + H)^+$ 458.9446, found 458.9445.

Diphenethylsulfane (3ab)[4]

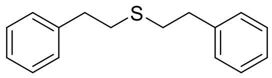

3ab

Following the general procedure, the reaction of **1a** (141.3 mg, 0.3 mmol, 1.5 equiv), **2b** (27 µL, 0.2 mmol, 1.0 equiv), DIPEA (71 µL, 0.4 mmol, 2.0 equiv) and anhydrous acetonitrile (2 mL) at room temperature for 16 h. After column chromatography on silica (eluent: petroleum ether), afford **3ab** (46.3 mg, 95%) as a colorless oil. **^{1}H NMR (400 MHz, Chloroform-d)** δ 7.38 – 7.29 (m, 4H), 7.29 – 7.18 (m, 6H), 2.96 – 2.87 (m, 4H), 2.87 – 2.77 (m, 4H) ppm. **^{13}C NMR (101 MHz, Chloroform-d)** δ 140.6, 128.5, 126.4, 36.4, 33.8 ppm. **HRMS m/z (ESI):** calcd for $C_{16}H_{18}NaS$ $(M + Na)^{+}$ 265.1021, found 265.1003.

Phenethyl(*p*-tolyl)sulfane (3ac)[5]

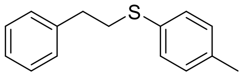

3ac

Following the general procedure, the reaction of **1a** (141.3 mg, 0.3 mmol, 1.5 equiv), **2c** (25 µL, 0.2 mmol, 1.0 equiv), DIPEA (71 µL, 0.4 mmol, 2.0 equiv) and anhydrous acetonitrile (2 mL) at room temperature for 16 h. After column chromatography on silica (eluent: petroleum ether), afford **3ea** (28.0 mg, 61%) as a colorless oil. **^{1}H NMR (400 MHz, Chloroform-d)** δ 7.35 – 7.26 (m, 4H), 7.25 – 7.17 (m, 3H), 7.13 (d, J = 7.9 Hz, 2H), 3.18 – 3.10 (m, 2H), 2.96 – 2.87 (m, 2H), 2.34 (s, 3H) ppm. **^{13}C NMR (101 MHz, Chloroform-d)** δ 140.3, 136.2, 132.5, 130.1, 129.7, 128.5, 126.4, 35.84, 35.77, 21.0 ppm. **HRMS m/z (ESI):** calcd for $C_{15}H_{16}Na$ S $(M + Na)^{+}$ 251.0865, found 251.0857.

(4-(*tert*-Butyl)phenyl)(phenethyl)sulfane (3ad)

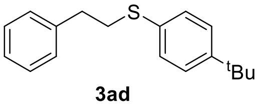

3ad

Following the general procedure, the reaction of **1a** (141.3 mg, 0.3 mmol, 1.5 equiv), **2d** (38.3 mg, 0.2 mmol, 1.0 equiv), DIPEA (71 µL, 0.4 mmol, 2.0 equiv) and anhydrous acetonitrile (2 mL) at room temperature for 16 h. After column chromatography on silica (eluent: petroleum ether), afford **3ad** (51.4 mg, 95%) as a colorless oil. **^{1}H NMR (400 MHz, Chloroform-d)** δ 7.40 – 7.31 (m, 6H), 7.26 (t, *J* = 8.7 Hz, 3H), 3.23 – 3.16 (m, 2H), 3.02 – 2.93 (m, 2H), 1.36 (s, 9H) ppm. **^{13}C NMR (101 MHz, Chloroform-d)**δ 149.3, 140.4, 132.7, 129.5, 128.5, 126.4, 126.0, 35.9, 35.5, 34.5, 31.3 ppm. **HRMS m/z (ESI):** calcd for $C_{18}H_{22}NaS$ $(M + Na)^+$ 293.1334, found 293.1331.

(4-Methoxyphenyl)(phenethyl)sulfane (3ae)[6]

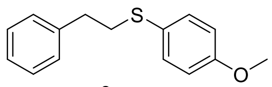

3ae

Following the general procedure, the reaction of **1a** (141.3 mg, 0.3 mmol, 1.5 equiv), **2e** (25 µL, 0.2 mmol, 1.0 equiv), DIPEA (71 µL, 0.4 mmol, 2.0 equiv) and anhydrous acetonitrile (2 mL) at room temperature for 16 h. After column chromatography on silica (eluent: petroleum ether/ethyl acetate 100:1), afford **3ae** (39.2 mg, 80%) as a colorless oil. **^{1}H NMR (400 MHz, Chloroform-d)** δ 7.39 (d, *J* = 7.9 Hz, 2H), 7.31 (t, *J* = 7.6 Hz, 2H), 7.23 (d, *J* = 6.9 Hz, 1H), 7.19 (d, *J* = 7.6 Hz, 2H), 6.88 (d, *J* = 8.8 Hz, 2H), 3.82 (s, 3H), 3.13 – 3.05 (m, 2H), 2.93 – 2.84 (m, 2H) ppm. **^{13}C NMR (101 MHz, Chloroform-d)** δ 158.9, 140.4, 133.3, 128.5, 128.4, 126.4, 114.6, 55.4, 37.3, 35.9 ppm. **HRMS m/z (ESI):** calcd for $C_{15}H_{16}NaOS$ $(M + Na)^+$ 267.0814, found 267.0794.

(4-Chlorophenyl)(phenethyl)sulfane (3af)[5]

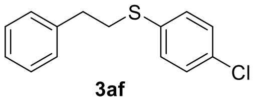

3af

Following the general procedure, the reaction of **1a** (141.3 mg, 0.3 mmol, 1.5 equiv), **2f** (28.9 mg, 0.2 mmol, 1.0 equiv), DIPEA (71 µL, 0.4 mmol, 2.0 equiv) and anhydrous acetonitrile (2 mL) at room temperature for 16 h. After column chromatography on silica (eluent:petroleum ether), afford **3af** (36.6mg, 74%) as a colorless oil. **^{1}H NMR**

(400 MHz, Chloroform-d) δ 7.35 – 7.22 (m, 7H), 7.20 (d, J = 6.9 Hz, 2H), 3.20 – 3.12 (m, 2H), 2.97 – 2.87 (m, 2H) ppm. **^{13}C NMR (101 MHz, Chloroform-d)** δ 139.9, 134.9, 132.0, 130.6, 129.1, 128.6, 128.5, 126.6, 35.5, 35.4 ppm. **HRMS m/z (ESI):** calcd for $C_{14}H_{13}ClNaS$ (M + Na)$^+$ 271.0319, found 271.0297.

Phenethyl(4-(trifluoromethyl)phenyl)sulfane (3ag)

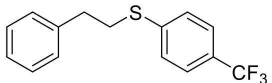

3ag

Following the general procedure, the reaction of **1a** (141.3 mg, 0.3 mmol, 1.5 equiv), **2g** (28 μL, 0.2 mmol, 1.0 equiv), DIPEA (71 μL, 0.4 mmol, 2.0 equiv) and anhydrous acetonitrile (2 mL) at room temperature for 16 h. After column chromatography on silica (eluent: petroleum ether), afford **3ag** (36.0 mg, 64%) as a colorless oil. **^{1}H NMR (400 MHz, Chloroform-d)** δ 7.53 (d, J = 8.2 Hz, 2H), 7.41 – 7.29 (m, 4H), 7.24 (dd, J = 15.8, 6.9 Hz, 3H), 3.28 – 3.19 (m, 2H), 3.02 – 2.93 (m, 2H) ppm. **^{13}C NMR (101 MHz, Chloroform-d)** δ 142.1, 139.7, 128.6 (d, J = 13.1 Hz), 127.4, 126.7, 125.7 (q, J = 3.7 Hz), 124.2 (q, J = 271.6 Hz), 35.2, 33.9 ppm. **^{19}F NMR (471 MHz, Chloroform-d)** δ -62.4 ppm. **HRMS m/z (ESI)**: calcd for $C_{15}H_{14}F_3S$ (M + H)$^+$ 283.0763, found 283.0755.

(3-Bromophenyl)(phenethyl)sulfane (3ah)

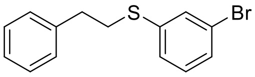

3ah

Following the general procedure, the reaction of **1a** (141.3 mg, 0.3 mmol, 1.5 equiv), **2h** (21 μL, 0.2 mmol, 1.0 equiv), DIPEA (71 μL, 0.4 mmol, 2.0 equiv) and anhydrous acetonitrile (2 mL) at room temperature for 16 h. After column chromatography on silica (eluent: petroleum ether), afford **3ah** (47.4 mg, 81%) as a colorless oil. **^{1}H NMR (400 MHz, Chloroform-d)** δ 7.47 (s, 1H), 7.33 (t, J = 7.3 Hz, 3H), 7.24 (dd, J = 16.7, 7.5 Hz, 4H), 7.15 (t, J = 7.9 Hz, 1H), 3.22 – 3.15 (m, 2H), 2.99 – 2.91 (m, 2H) ppm.

^{13}C NMR (101 MHz, Chloroform-d) δ 139.8, 139.0, 131.1, 130.2, 128.8, 128.6, 128.5, 127.2, 126.6, 122.9, 35.4, 34.8 ppm. **HRMS m/z (ESI):** calcd for $C_{14}H_{13}BrNaS$ (M + Na)$^+$ 314.9814, found 314.9803.

(3-Chlorophenyl)(phenethyl)sulfane (3ai)

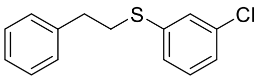

3ai

Following the general procedure, the reaction of **1a** (141.3 mg, 0.3 mmol, 1.5 equiv), **2i** (23 μL, 0.2 mmol, 1.0 equiv), DIPEA (71 μL, 0.4 mmol, 2.0 equiv) and anhydrous acetonitrile (2 mL) at room temperature for 16 h. After column chromatography on silica (eluent: petroleum ether), afford **3ai** (40.8 mg, 82%) as a colorless oil. **^{1}H NMR (400 MHz, Chloroform-d)** δ 7.38 (d, J = 7.1 Hz, 1H), 7.36 – 7.19 (m, 7H), 7.12 (t, J = 7.1 Hz, 1H), 3.26 – 3.14 (m, 2H), 3.03 – 2.93 (m, 2H) ppm. **^{13}C NMR (101 MHz, Chloroform-d)** δ 140.0, 135.8, 133.6, 129.7, 128.6, 128.5, 128.4, 127.1, 126.6, 126.5, 35.2, 33.9 ppm. **HRMS m/z (ESI):** calcd for $C_{14}H_{13}ClNaS$ (M + Na)$^+$ 271.0319, found 271.0297.

(2-Fluorophenyl)(phenethyl)sulfane (3aj)

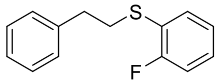

3aj

Following the general procedure, the reaction of **1a** (141.3 mg, 0.3 mmol, 1.5 equiv), **2j** (22 μL, 0.2 mmol, 1.0 equiv), DIPEA (71 μL, 0.4 mmol, 2.0 equiv) and anhydrous acetonitrile (2 mL) at room temperature for 16 h. After column chromatography on silica (eluent: petroleum ether), afford **3aj** (40.5 mg, 87%) as a colorless oil. **^{1}H NMR (400 MHz, Chloroform-d)** δ 7.44 – 7.37 (m, 1H), 7.31 (t, J = 7.2 Hz, 2H), 7.27 – 7.17 (m, 4H), 7.14 – 7.04 (m, 2H), 3.22 – 3.12 (m, 2H), 2.98 – 2.87 (m, 2H) ppm. **^{13}C NMR (101 MHz, Chloroform-d)** δ 161.5 (d, J = 245.4 Hz), 140.0, 132.2 (d, J = 15.1 Hz), 128.6, 128.5, 126.5, 124.4, 123.0 (d, J = 17.8 Hz), 115.7 (d, J = 22.0 Hz), 35.8, 34.8 ppm. **^{19}F NMR (376 MHz, Chloroform-d)** δ -109.3 ppm. **HRMS m/z (ESI):** calcd for

$C_{14}H_{13}FNaS$ (M + Na)$^+$ 255.0614, found 255.0610.

(2,6-Dimethylphenyl)(phenethyl)sulfane (3ak)

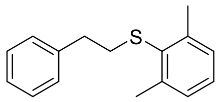

3ak

Following the general procedure, the reaction of **1a** (141.3 mg, 0.3 mmol, 1.5 equiv), **2k** (27 μL, 0.2 mmol, 1.0 equiv), DIPEA (71 μL, 0.4 mmol, 2.0 equiv) and anhydrous acetonitrile (2 mL) at room temperature for 16 h. After column chromatography on silica (eluent: petroleum ether), afford **3ak** (31.8 mg, 54%) as a colorless oil. **^{1}H NMR (400 MHz, Chloroform-d)** δ 7.29 (d, J = 7.0 Hz, 2H), 7.24 – 7.06 (m, 6H), 2.98 – 2.88 (m, 2H), 2.88 – 2.79 (m, 2H), 2.54 (s, 6H) ppm. **^{13}C NMR (101 MHz, Chloroform-d)** δ 143.1, 140.5, 133.4, 128.4, 128.4, 128.2, 128.1, 126.3, 36.5, 36.4, 22.1 ppm. **HRMS m/z (ESI):** calcd for $C_{16}H_{18}NaS$ (M + Na)$^+$ 265.1021, found 265.1008.

2-(Phenethylthio)pyridine(3al)[7]

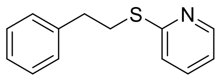

3al

Following the general procedure, the reaction of **1a** (141.3 mg, 0.3 mmol, 1.5 equiv), **2l** (23 μL, 0.2 mmol, 1.0 equiv), DIPEA (71 μL, 0.4 mmol, 2.0 equiv) and anhydrous acetonitrile (2 mL) at room temperature for 16 h. After column chromatography on silica (eluent: petroleum ether), afford **3al** (37.8 mg, 90%) as a colorless oil. **^{1}H NMR (400 MHz, Chloroform-d)** δ 8.50 – 8.42 (m, 1H), 7.51 – 7.43 (m, 1H), 7.35 – 7.20 (m, 5H), 7.17 (d, J = 8.1 Hz, 1H), 6.98 (dd, J = 7.3, 4.9 Hz, 1H), 3.48 – 3.40 (m, 2H), 3.07 – 2.99 (m, 2H) ppm. **^{13}C NMR (101 MHz, Chloroform-d)** δ 158.9, 149.4, 140.5, 135.9, 128.6, 128.4, 126.4, 122.4, 119.3, 35.8, 31.5 ppm. **HRMS m/z (ESI):** calcd for $C_{13}H_{13}NNaS$ (M + Na)$^+$ 238.0661, found 238.0644.

2-(Phenethylthio)thiophene (3am)

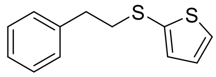

3am

Following the general procedure, the reaction of **1a** (141.3 mg, 0.3 mmol, 1.5 equiv), **2m** (19 µL, 0.2 mmol, 1.0 equiv), DIPEA (71 µL, 0.4 mmol, 2.0 equiv) and anhydrous acetonitrile (2 mL) at room temperature for 16 h. After column chromatography on silica (eluent: petroleum ether), afford **3am** (31.5 mg, 71%) as a colorless oil. **^{1}H NMR (400 MHz, Chloroform-d)** δ 7.37 (d, *J* = 4.6 Hz, 1H), 7.30 (t, *J* = 7.3 Hz, 2H), 7.26 – 7.12 (m, 4H), 7.00 (dd, *J* = 5.3, 3.5 Hz, 1H), 3.09 – 3.01 (m, 2H), 2.96 – 2.88 (m, 2H) ppm. **^{13}C NMR (101 MHz, Chloroform-d)** δ 140.0, 134.4, 133.6, 129.2, 128.6, 128.5, 127.6, 126.4, 40.1, 36.0 ppm. **HRMS m/z (ESI):** calcd for $C_{12}H_{12}NaS_2$ $(M + Na)^+$ 243.0273, found 243.0258.

2-(Phenethylthio)benzo[*d*]oxazole (3an)

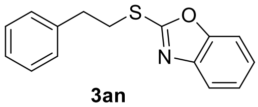

3an

Following the general procedure, the reaction of **1a** (141.3 mg, 0.3 mmol, 1.5 equiv), **2n** (30.2 mg, 0.2 mmol, 1.0 equiv), DIPEA (71 µL, 0.4 mmol, 2.0 equiv) and anhydrous acetonitrile (2 mL) at room temperature for 16 h. After column chromatography on silica (eluent: petroleum ether), afford **3an** (44.6 mg, 87%) as a colorless oil. **^{1}H NMR (400 MHz, Chloroform-d)** δ 7.63 (d, *J* = 7.0 Hz, 1H), 7.44 (d, *J* = 7.6 Hz, 1H), 7.37 – 7.21 (m, 7H), 3.61 – 3.51 (m, 2H), 3.21 – 3.11 (m, 2H) ppm. **^{13}C NMR (101 MHz, Chloroform-d)** δ 164.8, 151.8, 142.0, 139.4, 128.7, 128.6, 126.8, 124.3, 123.8, 118.5, 109.9, 35.6, 33.5 ppm. **HRMS m/z (ESI):** calcd for $C_{15}H_{13}NNaOS$ $(M + Na)^+$ 278.0610, found 278.0640.

2-(Phenethylthio)-1*H*-benzo[*d*]imidazole (3ao)

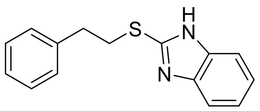

3ao

Following the general procedure, the reaction of **1a** (141.3 mg, 0.3 mmol, 1.5 equiv), **2o** (30.0 mg, 0.2 mmol, 1.0 equiv), DIPEA (71 μL, 0.4 mmol, 2.0 equiv) and anhydrous acetonitrile (2 mL) at room temperature for 16 h. After column chromatography on silica (eluent: petroleum ether), afford **3ao** (36.0 mg, 71%) as a white solid. **^{1}H NMR (400 MHz, Chloroform-d)** δ 7.52 (s, 2H), 7.30 – 7.14 (m, 7H), 3.55 (t, *J* = 7.4 Hz, 2H), 3.05 (t, *J* = 7.4 Hz, 2H) ppm. **^{13}C NMR (101 MHz, Chloroform-d)** δ 150.11, 139.60, 128.74, 128.52, 126.61, 122.33, 35.84, 34.09 ppm. **m.p.:**145.0-151.7 °C. **HRMS m/z (ESI):** calcd for $C_{15}H_{14}N_2NaS$ $(M + Na)^+$ 277.0770, found 277.0750.

General procedure for the synthesis of amination products:

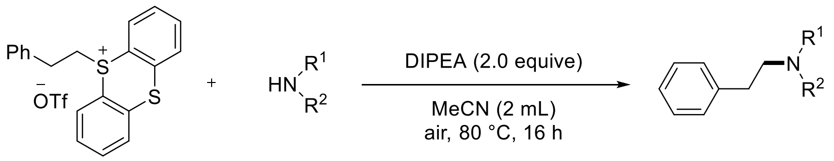

1a **2** **3**

To a 10 mL Schlenk tube was added alkyl sulfonate **1a** (0.3 mmol,1.5 equiv), amines **2** (0.2 mmol, 1.0 equiv), DIPEA (0.4 mmol, 2.0 equiv) and anhydrous acetonitrile (2 mL). The resulting mixture was stirred at 80 °C for 16 h. The reaction mixture was quenched with dichloromethane. The organic layer was concentrated and purified by silica gel chromatography (petroleum ether and ethyl acetate) to afford the desired product **3**.

N-Phenethylaniline (3ap)

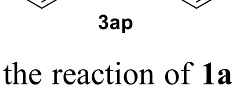

3ap

Following the general procedure, the reaction of **1a** (141.3 mg, 0.3 mmol, 1.5 equiv),

2p (18 μL, 0.2 mmol, 1.0 equiv), DIPEA (71 μL, 0.4 mmol, 2.0 equiv) and anhydrous acetonitrile (2 mL) at 80 °C for 16 h. After column chromatography on silica (eluent: petroleum ether), afford **3ap** (20.7 mg, 53%) as a Pale yellow. **^{1}H NMR (400 MHz, Chloroform-d)** δ 7.39 – 7.04 (m, 7H), 6.79 – 6.48 (m, 3H), 3.68 (s, 1H), 3.40 (t, *J* = 6.7 Hz, 2H), 2.92 (t, *J* = 6.6 Hz, 2H) ppm. **^{13}C NMR (101 MHz, Chloroform-d)** δ 148.0, 139.3, 129.3, 128.8, 128.6, 126.4, 117.5, 113.0, 45.0, 35.5 ppm. **HRMS m/z (ESI):** calcd for $C_{14}H_{15}NNa$ $(M + Na)^+$ 220.1097, found 220.1099.

N-Methyl-*N*-phenethylaniline (3aq)[8]

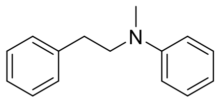

3aq

Following the general procedure, the reaction of **1a** (141.3 mg, 0.3 mmol, 1.5 equiv), **2q** (22 μL, 0.2 mmol, 1.0 equiv), DIPEA (71 μL, 0.4 mmol, 2.0 equiv) and anhydrous acetonitrile (2 mL) at 80 °C for 16 h. After column chromatography on silica (eluent: petroleum ether), afford 3aq (30.0 mg, 71%) as a colorless liquid. **^{1}H NMR (400 MHz, Chloroform-d)** δ 7.38 – 7.14 (m, 7H), 6.80 – 6.67 (m, 3H), 3.65 – 3.51 (m, 2H), 2.95 – 2.83 (m, 5H) ppm. **^{13}C NMR (101 MHz, Chloroform-d)** δ 148.8, 139.8, 129.3, 128.8, 128.5, 126.2, 116.2, 112.2, 54.8, 38.5, 32.9 ppm. **HRMS m/z (ESI):** calcd for $C_{15}H_{17}NNa$ $(M + Na)^+$ 234.1253, found 234.1261.

N-Methyl-*N*-(naphthalen-1-ylmethyl)-2-phenylethan-1-amine (3ar)

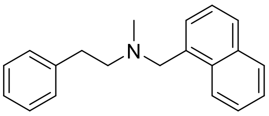

3ar

Following the general procedure, the reaction of **1a** (141.3 mg, 0.3 mmol, 1.5 equiv.), **2r** (33 μL, 0.2 mmol, 1.0 equiv), DIPEA (71 μL, 0.4 mmol, 2.0 equiv) and anhydrous acetonitrile (2 mL) at 80 ℃ for 16 h. After column chromatography on silica (eluent:

petroleum ether), afford **3ar** (48.6 mg, 88%) as a colorless oil. **^{1}H NMR (400 MHz, Chloroform-d)** δ 8.22 (s, 1H), 7.82 (d, J = 25.4 Hz, 2H), 7.45 (dd, J = 22.7, 4.8 Hz, 4H), 7.35 – 7.15 (m, 5H), 3.97 (s, 2H), 2.86 (dt, J = 40.6, 6.9 Hz, 4H), 2.32 (s, 3H) ppm. **^{13}C NMR (101 MHz, Chloroform-d)** δ 140.6, 134.7, 133.9, 132.5, 128.8, 128.4, 128.3, 128.0, 127.4, 125.9, 125.8, 125.6, 125.1, 124.7, 60.6, 59.8, 42.1, 33.7 ppm. **HRMS m/z (ESI):** calcd for $C_{20}H_{21}NNa$ $(M + Na)^+$ 298.1566, found 298.1555.

4. Mechanistic studies

Radical trapping experiment with TEMPO

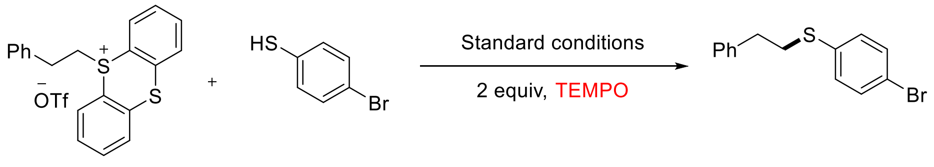

1a **2a** **3aa**, 46%

To a 10 mL Schlenk tube was added alkyl sulfonate **1a** (0.3 mmol, 141 mg, 1.5 equiv), thiophenol **2a** (0.2 mmol, 38.32 g, 1.0 equiv), DIPEA (0.4 mmol, 71 μL, 2.0 equiv), TEMPO (0.4 mmol, 62.5 mg, 2 equiv) and anhydrous acetonitrile (2 mL). The resulting mixture was stirred at room temperature for 16 h. After the reaction was completed, it was quenched with water. The residue was then extracted with CH_2Cl_2 (3 × 20 mL) and the combined organic layers were dried over anhydrous Na_2SO_4. The organic layer was concentrated in vacuo, resulting in a colorless oil. It was then concentrated and purified by silica gel chromatography to afford **3aa** (yield: 46%).

Radical trapping experiment with BHT

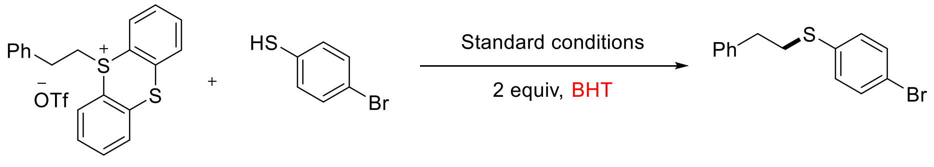

1a **2a** **3aa**, 83%

To a 10 mL Schlenk tube was added alkyl sulfonate **1a** (0.3 mmol, 141 mg, 1.5 equiv),

thiophenol **2a** (0.2 mmol, 38.32 g, 1.0 equiv), DIPEA (0.4 mmol, 71 μL, 2.0 equiv), BHT (0.4 mmol, 62.5 mg, 2 equiv) and anhydrous acetonitrile (2 mL). The resulting mixture was stirred at room temperature for 16 h. After the reaction was completed, it was quenched with water. The residue was then extracted with CH_2Cl_2 (3 × 20 mL) and the combined organic layers were dried over anhydrous Na_2SO_4. The organic layer was concentrated in vacuo, resulting in a colorless oil. It was then concentrated and purified by silica gel chromatography to afford **3aa** (yield: 83%).

5. References

1. C. Chen, M. Wang, H. Lu, B. Zhao, Z. Shi, *Angew. Chem. Int. Ed.* **2021**, *60*, 21756−21760.
2. D. V. Jawale, J.-A. Tchuiteng-Kouatchou, F. Fossard, F. Miserque, V. Geertsen, E. Gravel and E. Doris, *Green Chem.*, **2022**, 24 , 1231.
3. Y. Cai, F. Nie, Q. Song, *J. Org. Chem.* **2021**, *86*, 12419– 12426.
4. Shinkar, E. V.; Shvetsova, A. V.; Okhlobystin, A. O.; Berberova, N. T. *Russ J Electrochem*. **2020**. 56, 285–292.
5. Guo, Z.; Liu, X.; Bai, R.; Che, Y.; Chi, Y.; Guo, C.; Xing, H. *Inorg. Chem*. **2021**, 60, 8672– 8681.
6. F. Zhang, Y. Wang, Y. Wang, Y. Pan, *Org. Lett*. **2021**, 23, 7524−7528.
7. Z. Qiao, J. Wei, X. Jiang, *Org. Lett*. **2014**, 16, 1212−1215.
8. Y. Wei, Q. Xuan, Y. Zhou, Q. Song, *Org. Chem. Front*. **2018**, 5, 3510.

6. Spectra of ^1H NMR, ^{13}C NMR

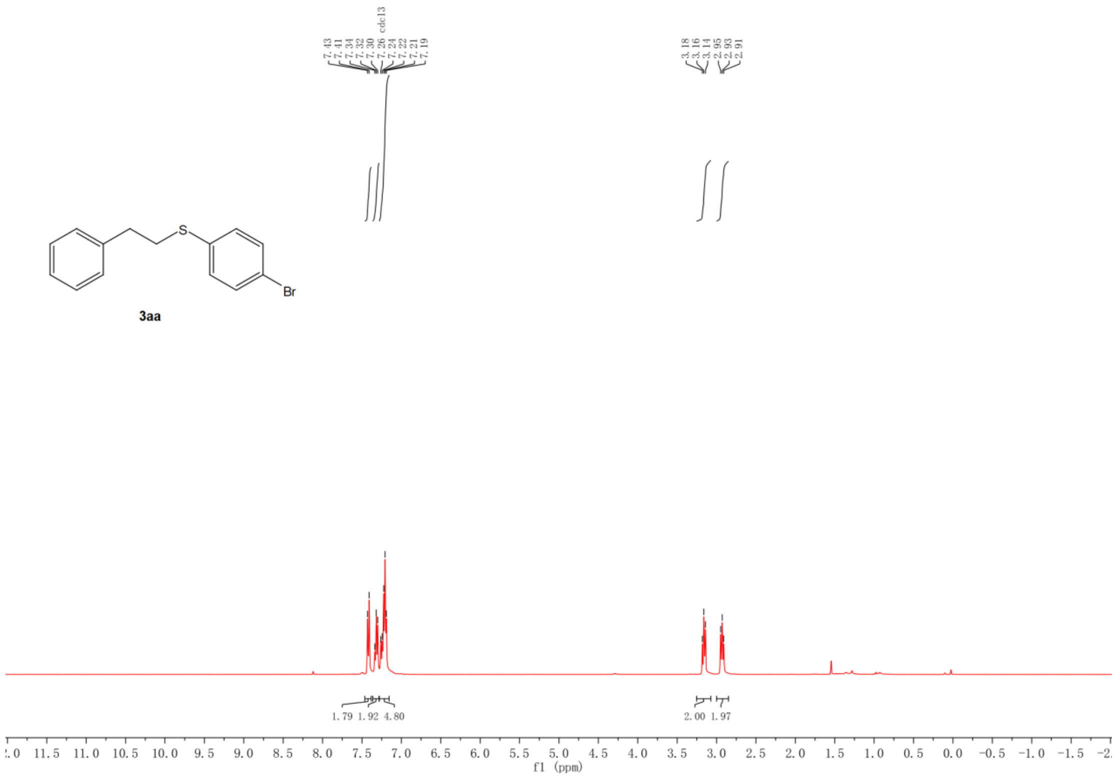

Figure S1. ^1H NMR spectra (400 MHz) of **3aa** in $CDCl_3$.

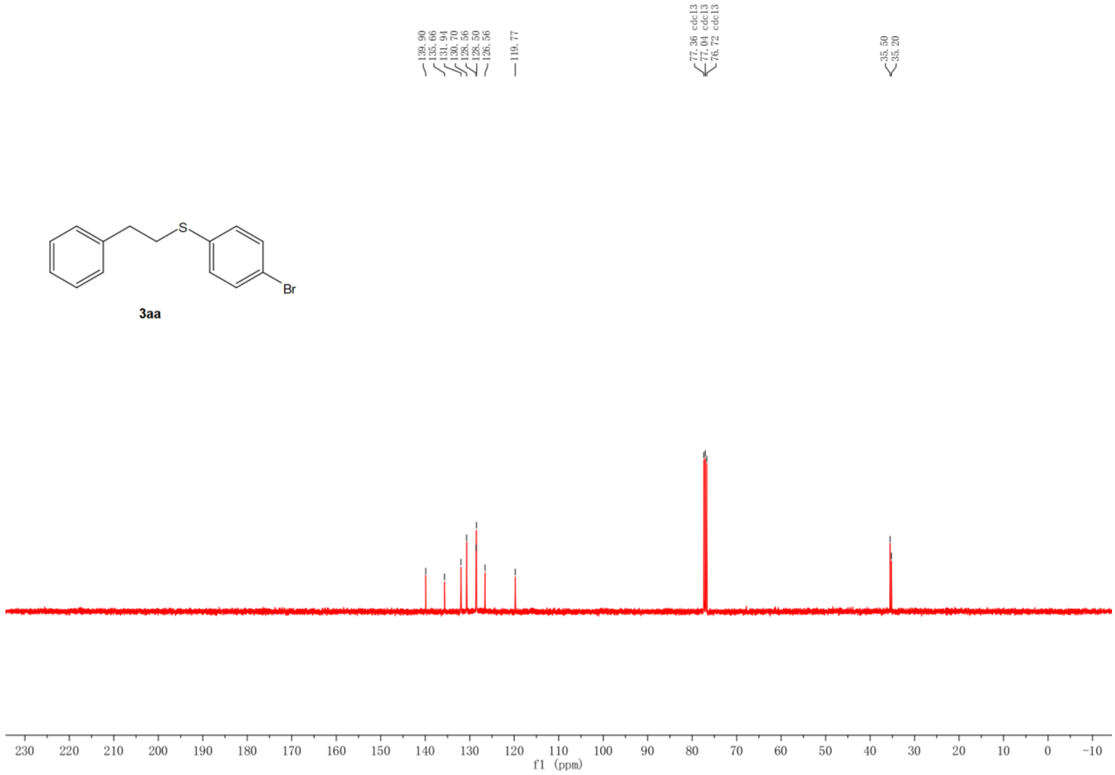

Figure S2. ^{13}C NMR spectra (400 MHz) of **3aa** in $CDCl_3$.

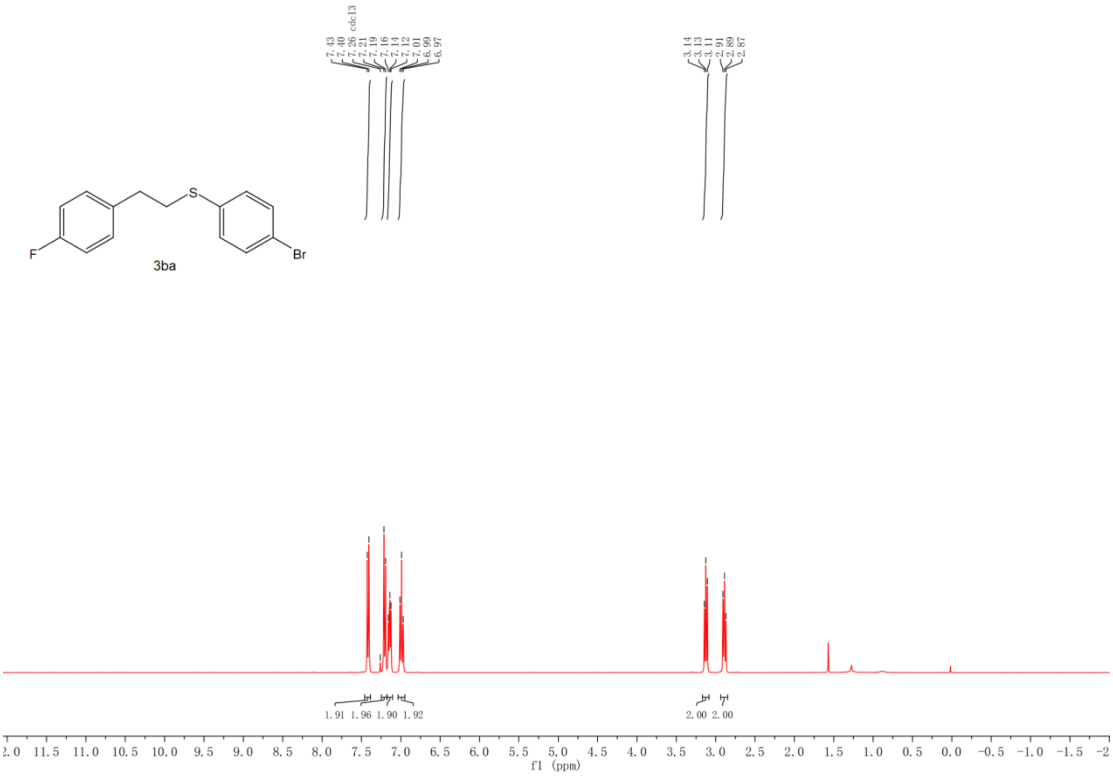

Figure S3. ^{1}H NMR spectra (400 MHz) of **3ba** in $CDCl_3$.

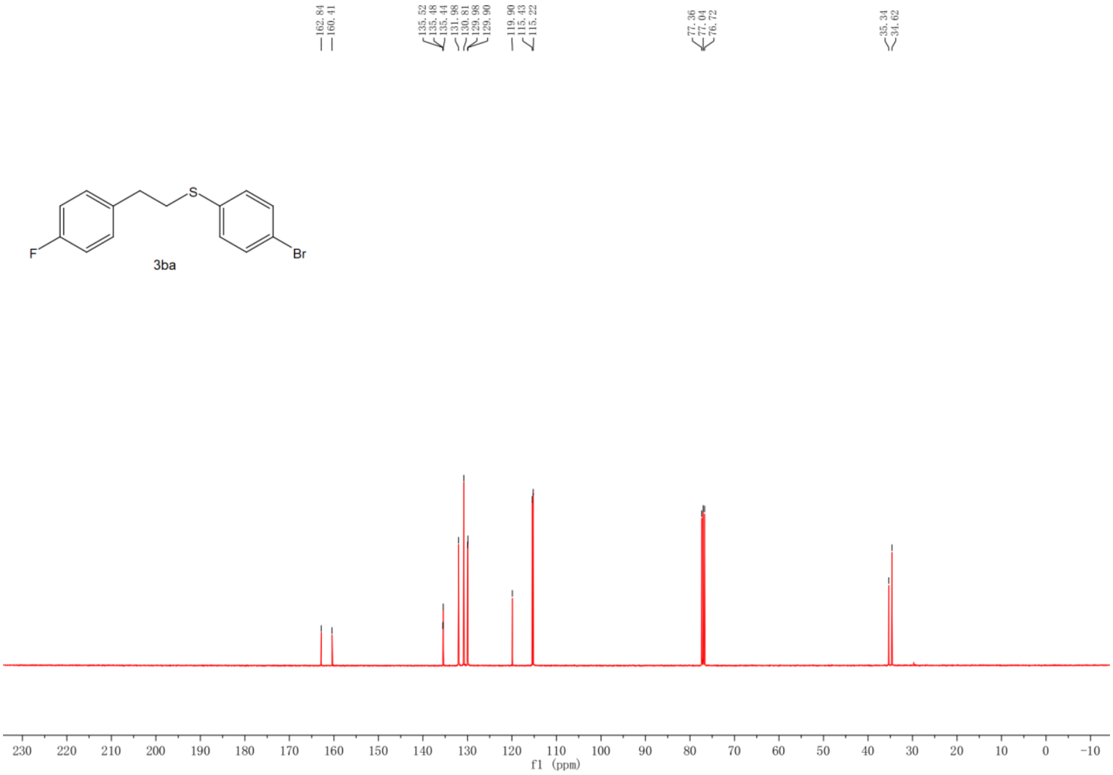

Figure S4. ^{13}C NMR spectra (400 MHz) of **3ba** in $CDCl_3$.

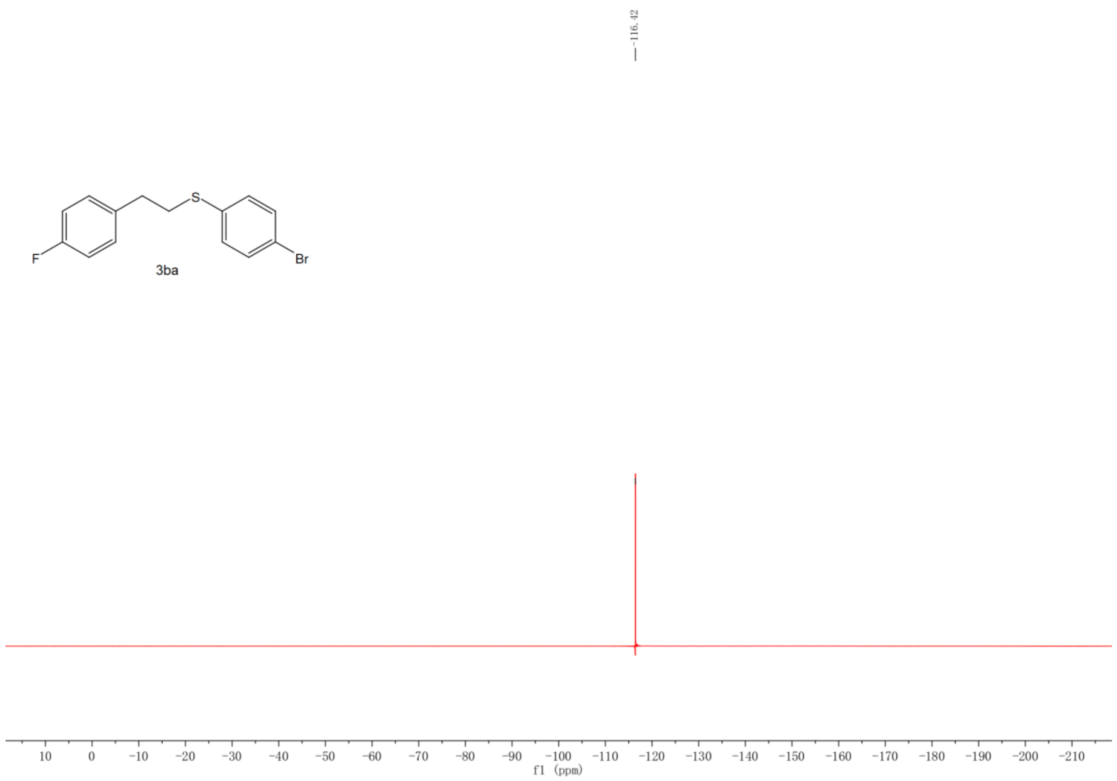

Figure S5. ^{19}F NMR spectra (400 MHz) of **3ba** in $CDCl_3$.

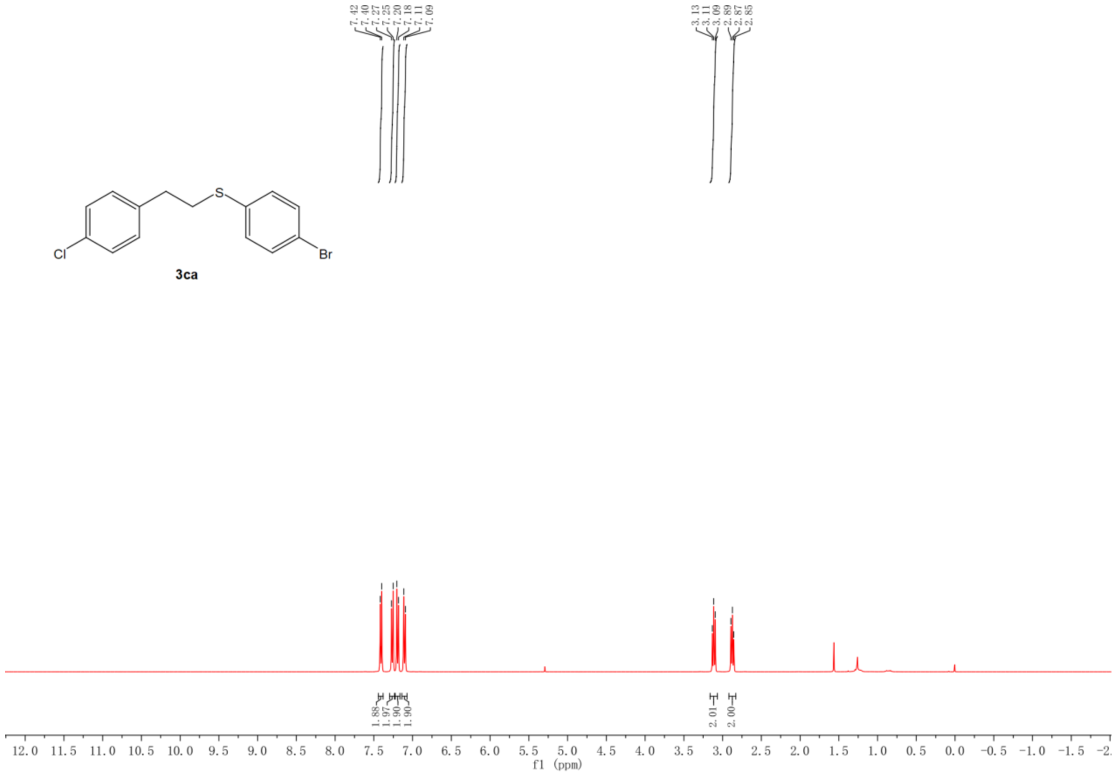

Figure S6. ^{1}H NMR spectra (400 MHz) of **3ca** in $CDCl_3$.

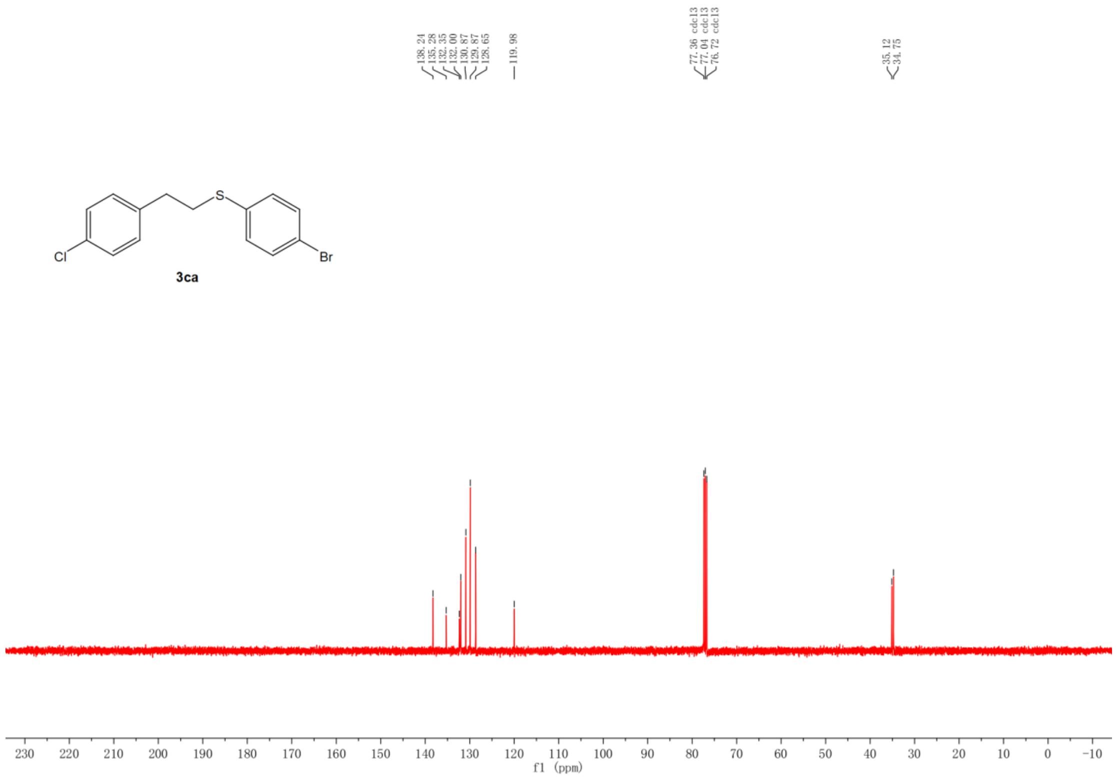

Figure S7. ^{13}C NMR spectra (400 MHz) of **3ca** in $CDCl_3$.

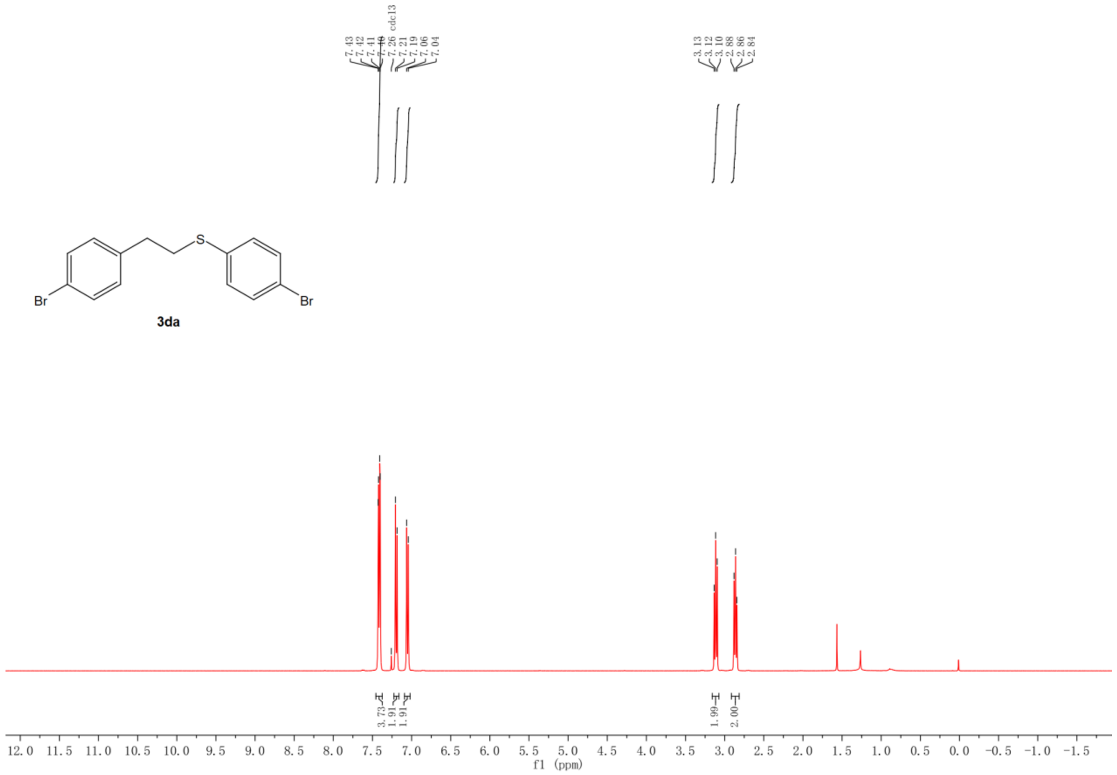

Figure S8. ^{1}H NMR spectra (400 MHz) of **3da** in $CDCl_3$.

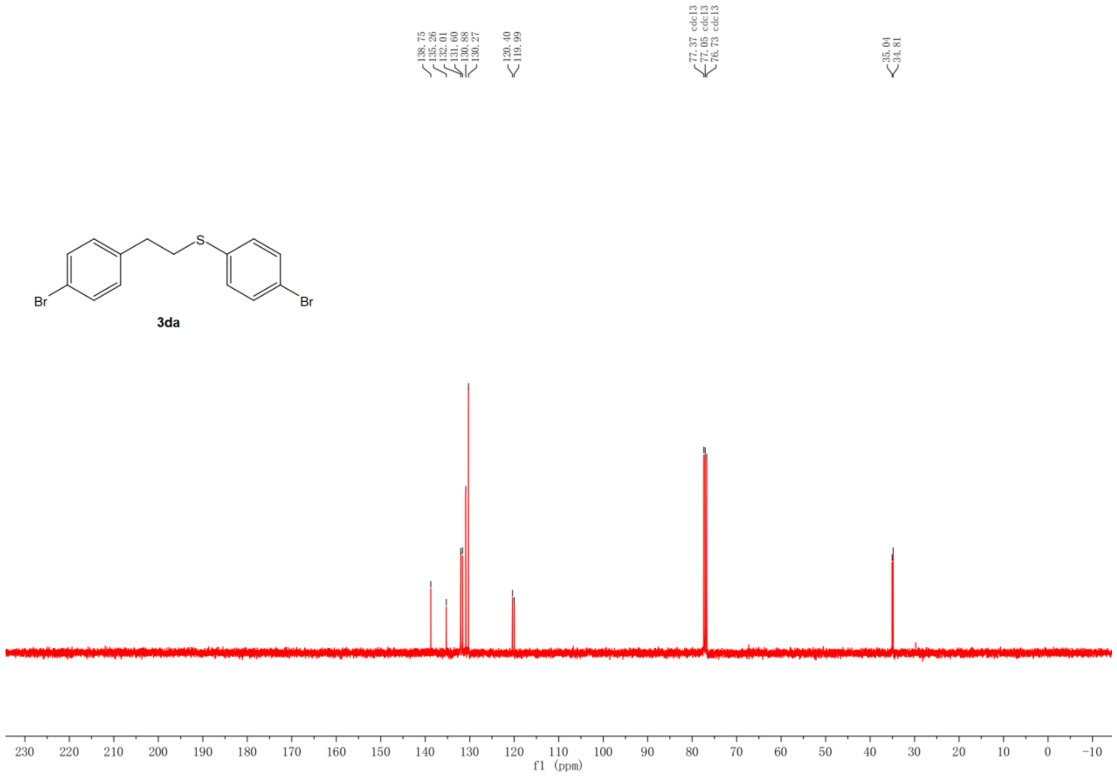

Figure S9. ^{13}C NMR spectra (400 MHz) of **3da** in $CDCl_3$.

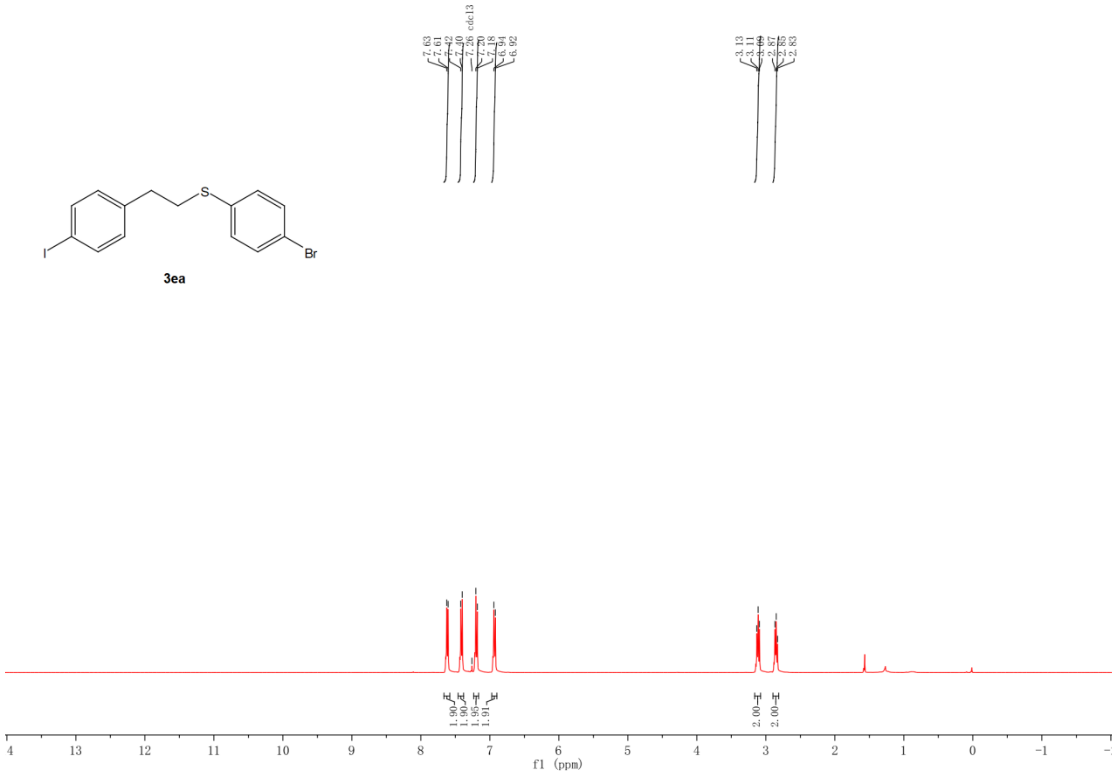

Figure S10. ^{1}H NMR spectra (400 MHz) of **3ea** in $CDCl_3$.

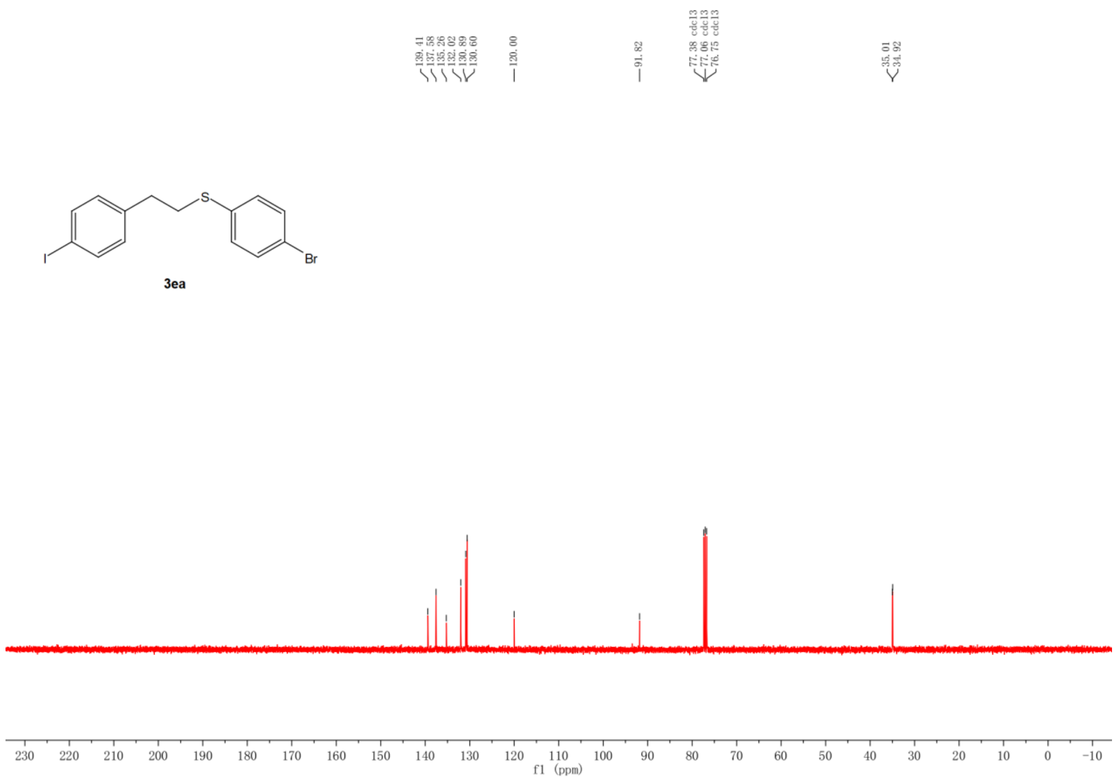

Figure S11. ^{13}C NMR spectra (400 MHz) of **3ea** in $CDCl_3$.

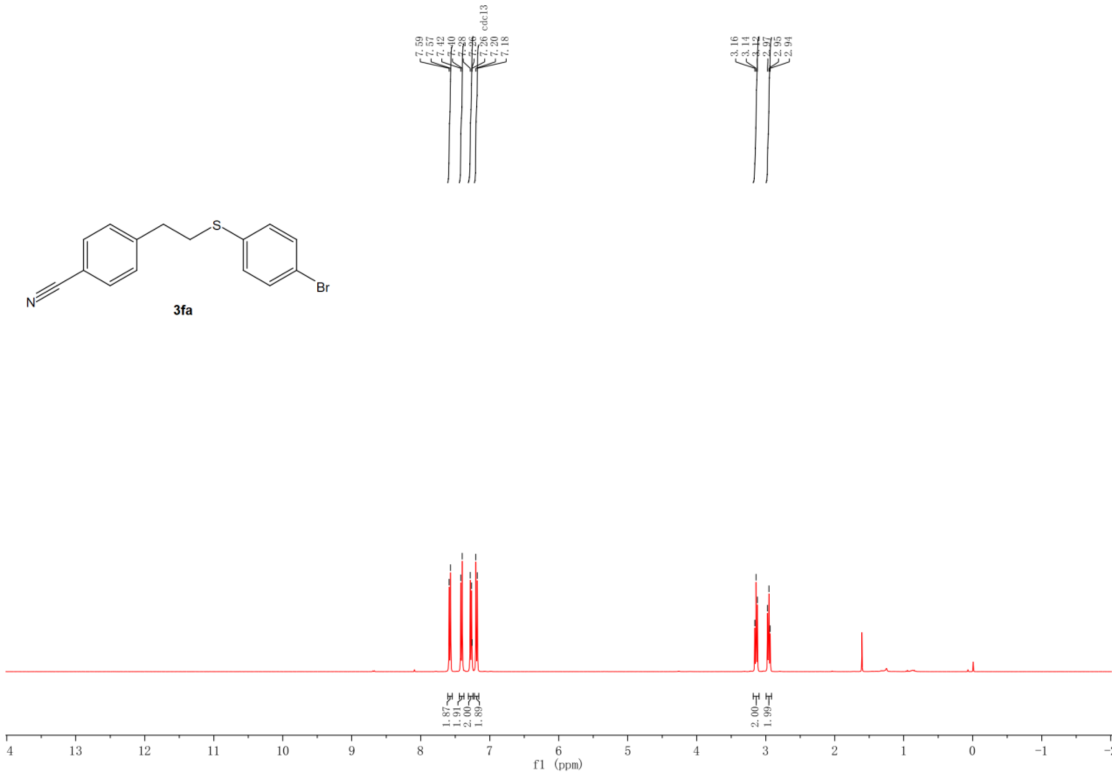

Figure S12. ^{1}H NMR spectra (400 MHz) of **3fa** in $CDCl_3$.

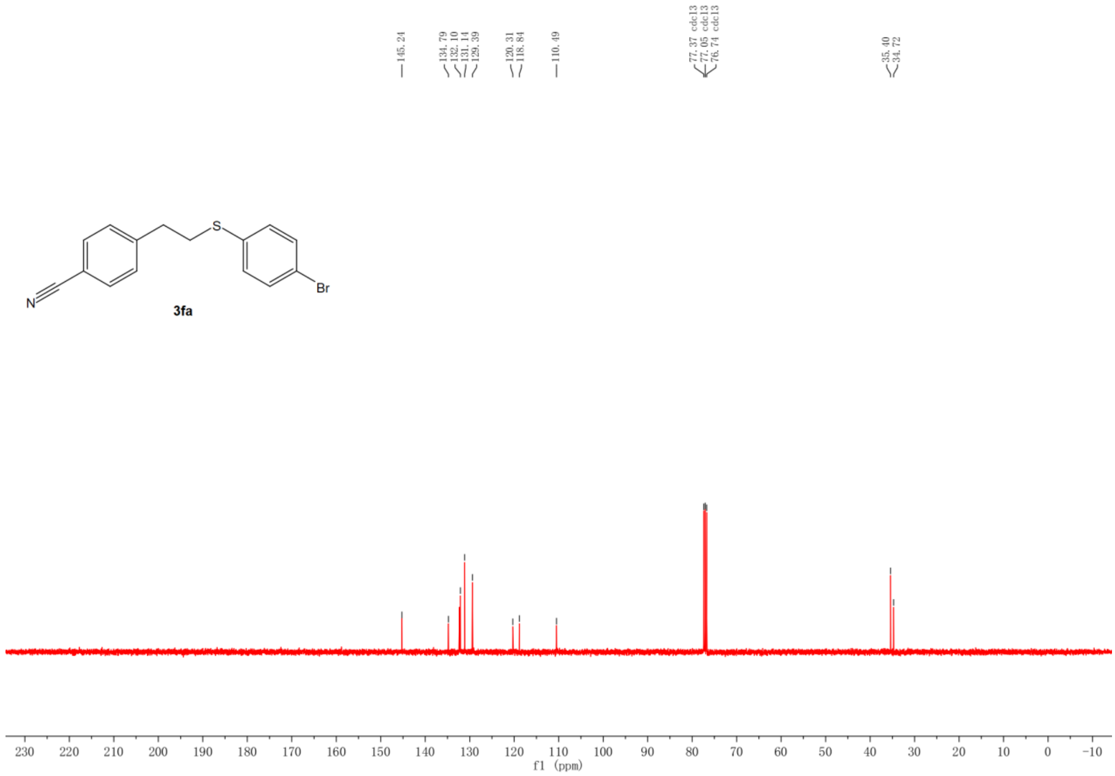

Figure S13. ^{13}C NMR spectra (400 MHz) of **3fa** in $CDCl_3$.

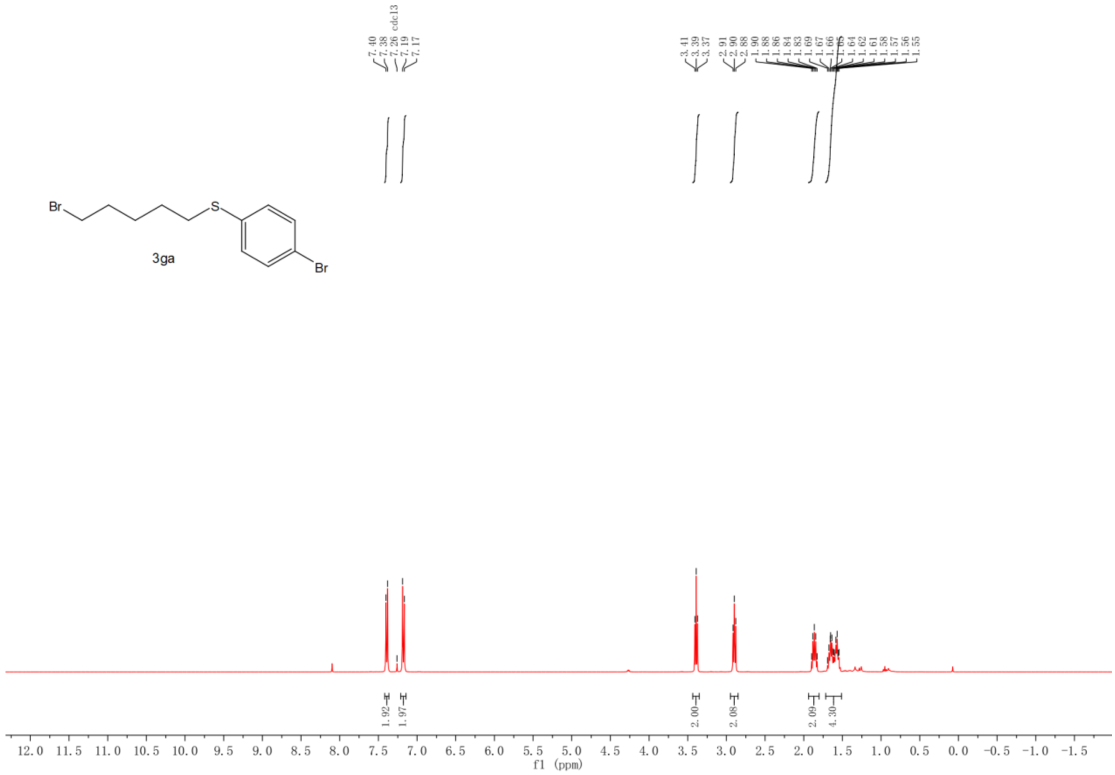

Figure S14. ^{1}H NMR spectra (400 MHz) of **3ga** in $CDCl_3$.

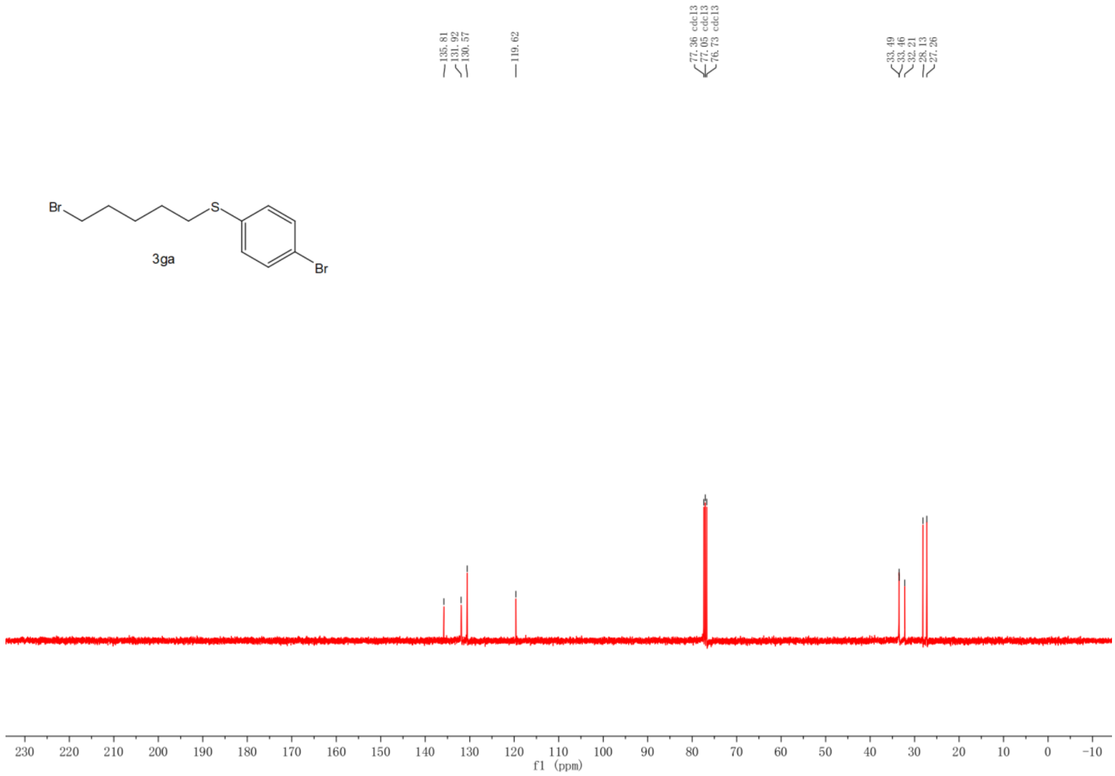

Figure S15. ^{13}C NMR spectra (400 MHz) of **3ga** in $CDCl_3$.

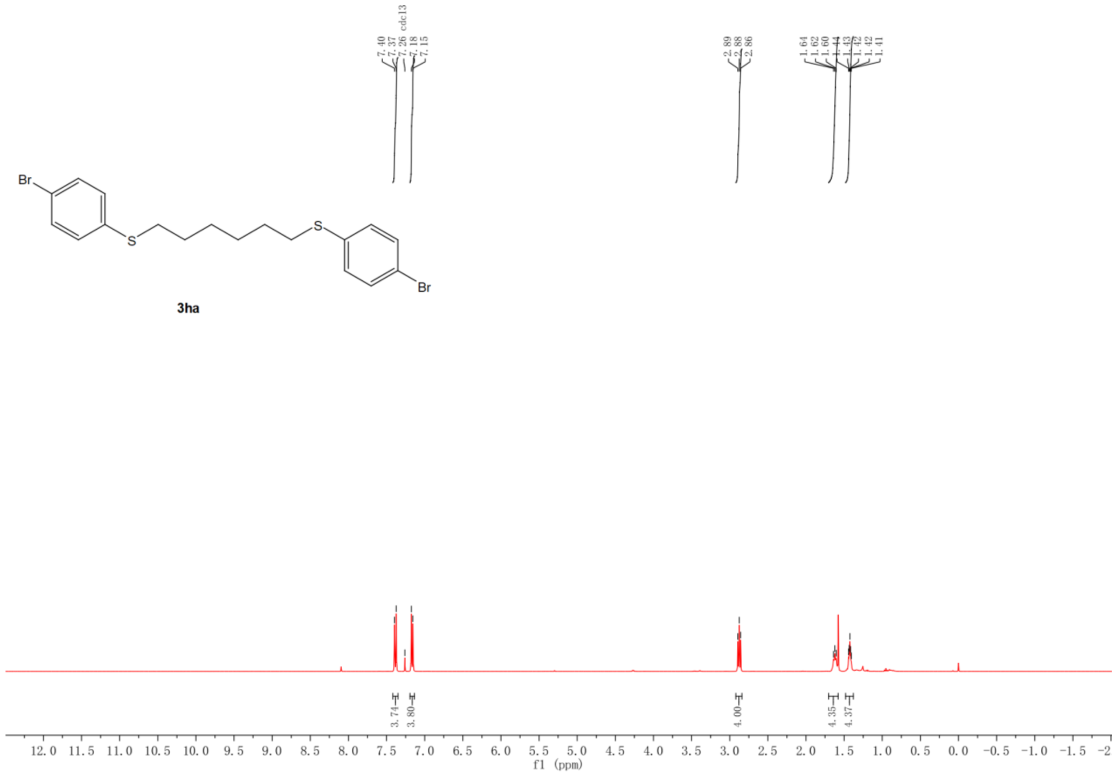

Figure S16. ^{1}H NMR spectra (400 MHz) of **3ha** in $CDCl_3$.

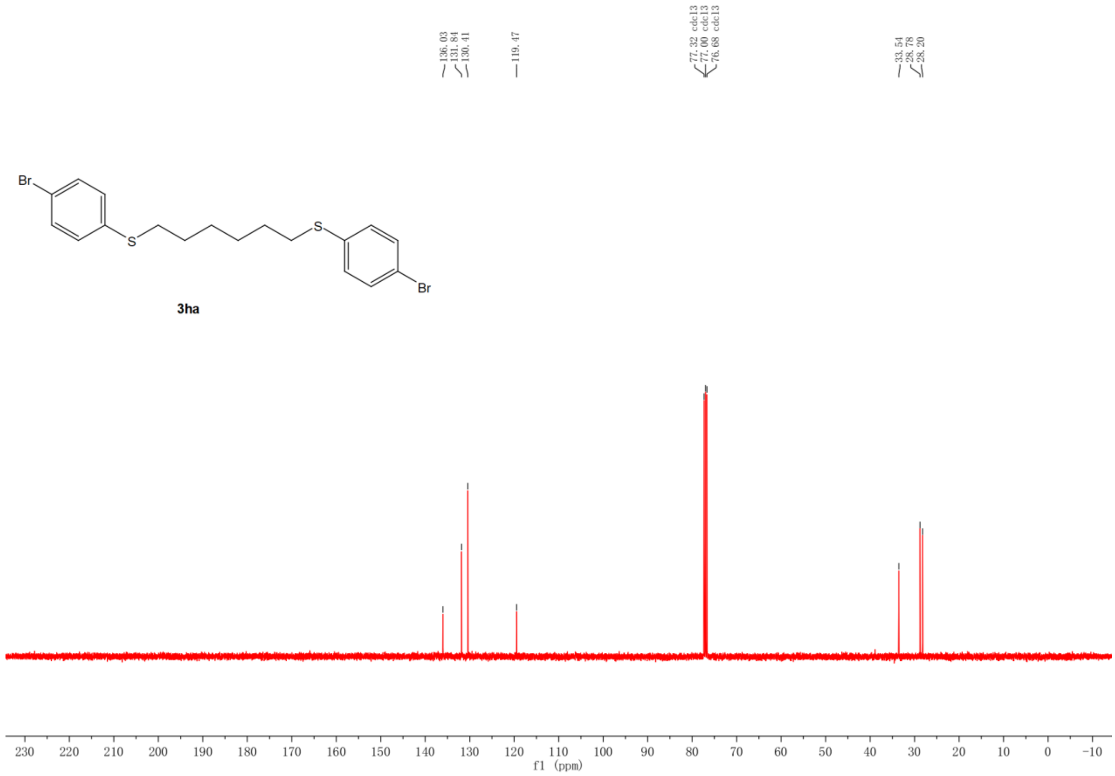

3ha

Figure S17. ^{13}C NMR spectra (400 MHz) of **3ha** in $CDCl_3$.

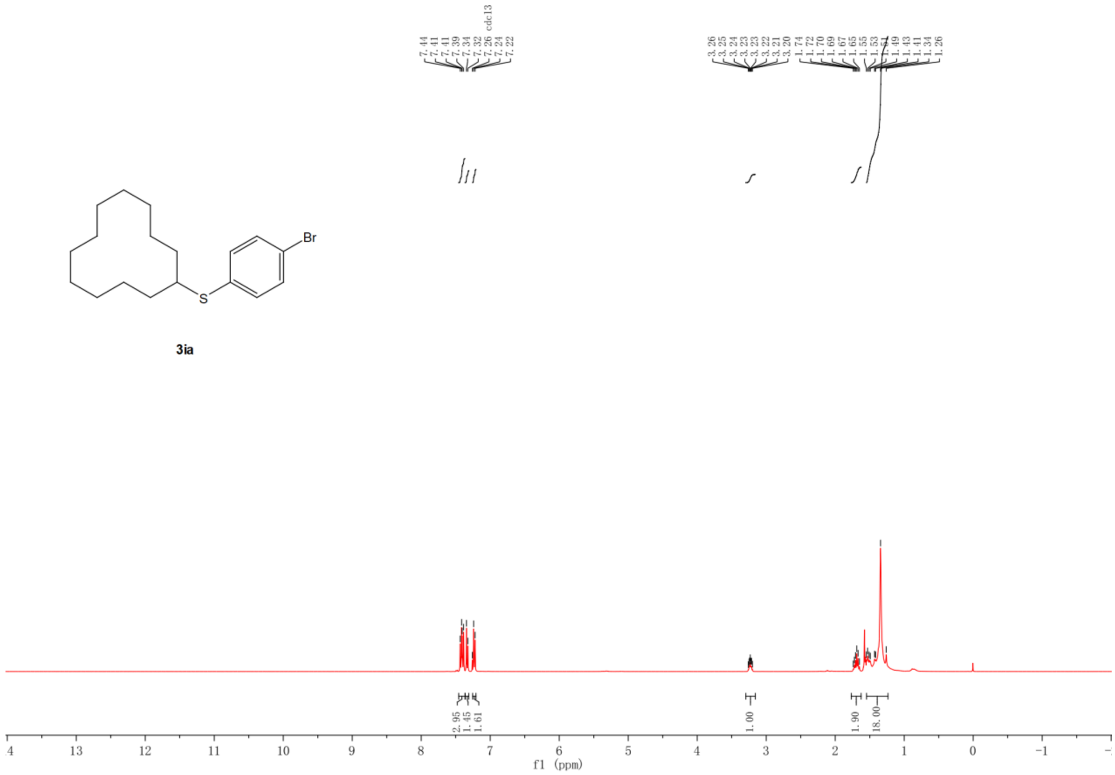

3ia

Figure S18. ^{1}H NMR spectra (400 MHz) of **3ia** in $CDCl_3$.

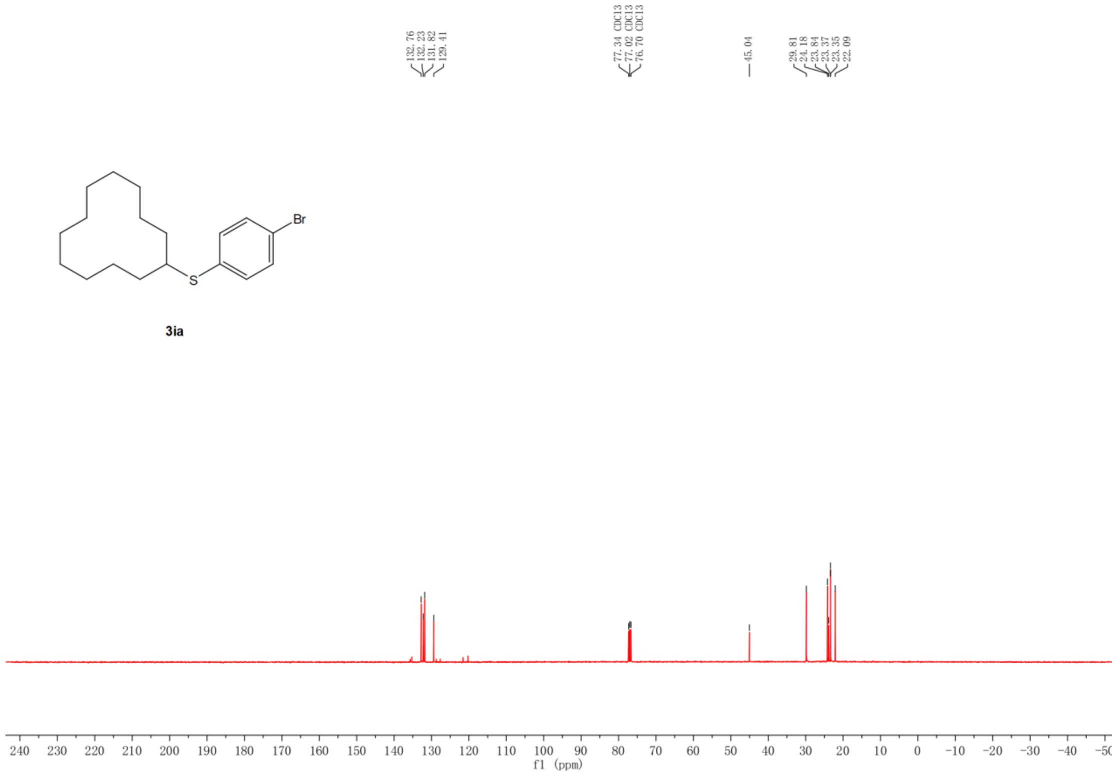

Figure S19. ^{13}C NMR spectra (400 MHz) of **3ia** in $CDCl_3$.

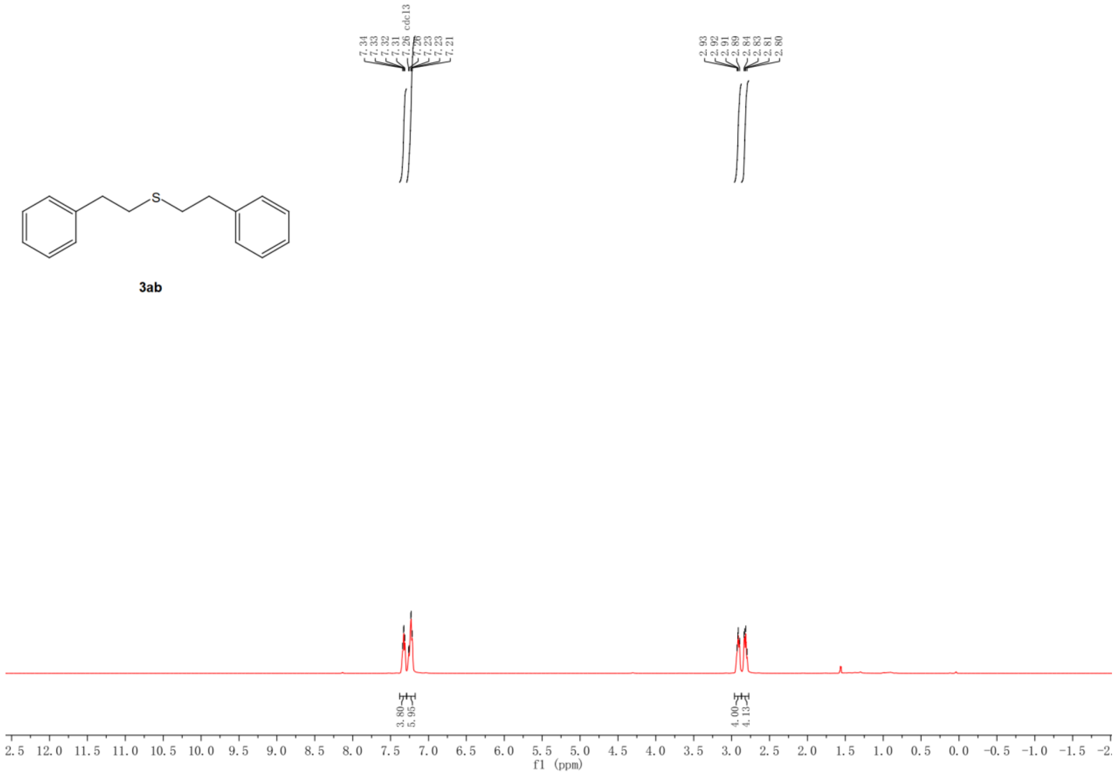

Figure S20. ^{1}H NMR spectra (400 MHz) of **3ab** in $CDCl_3$.

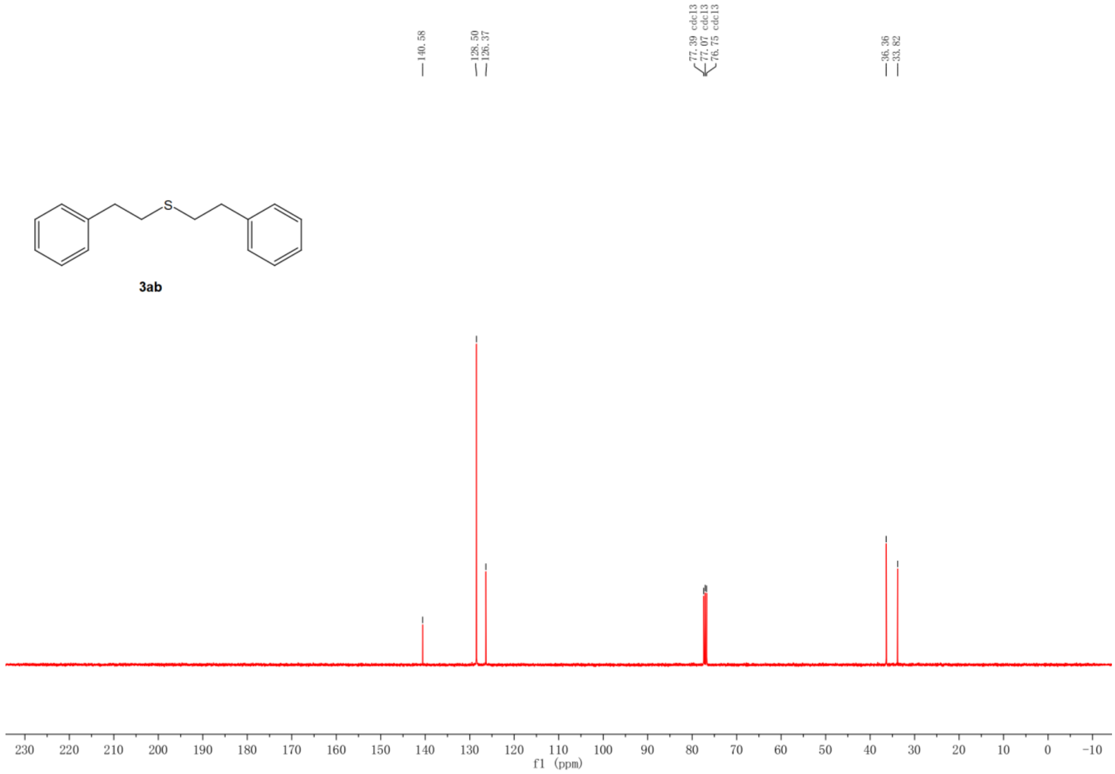

Figure S21. ^{13}C NMR spectra (400 MHz) of **3ab** in $CDCl_3$.

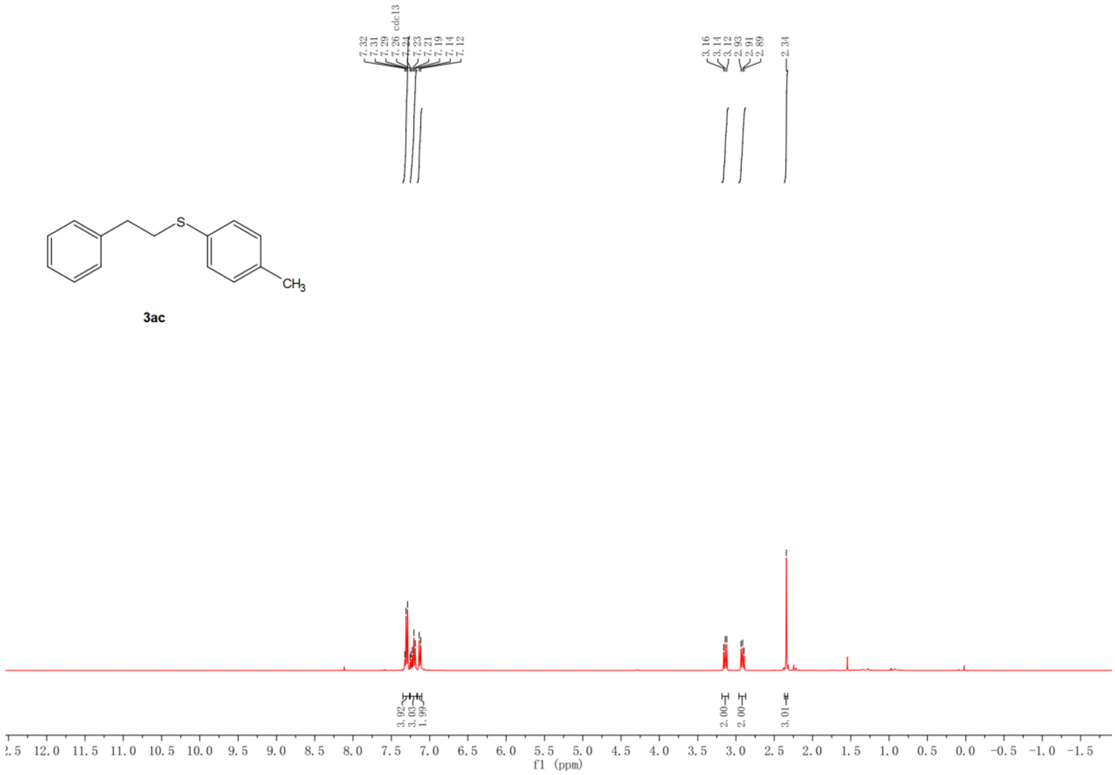

Figure S22. ^{1}H NMR spectra (400 MHz) of **3ac** in $CDCl_3$.

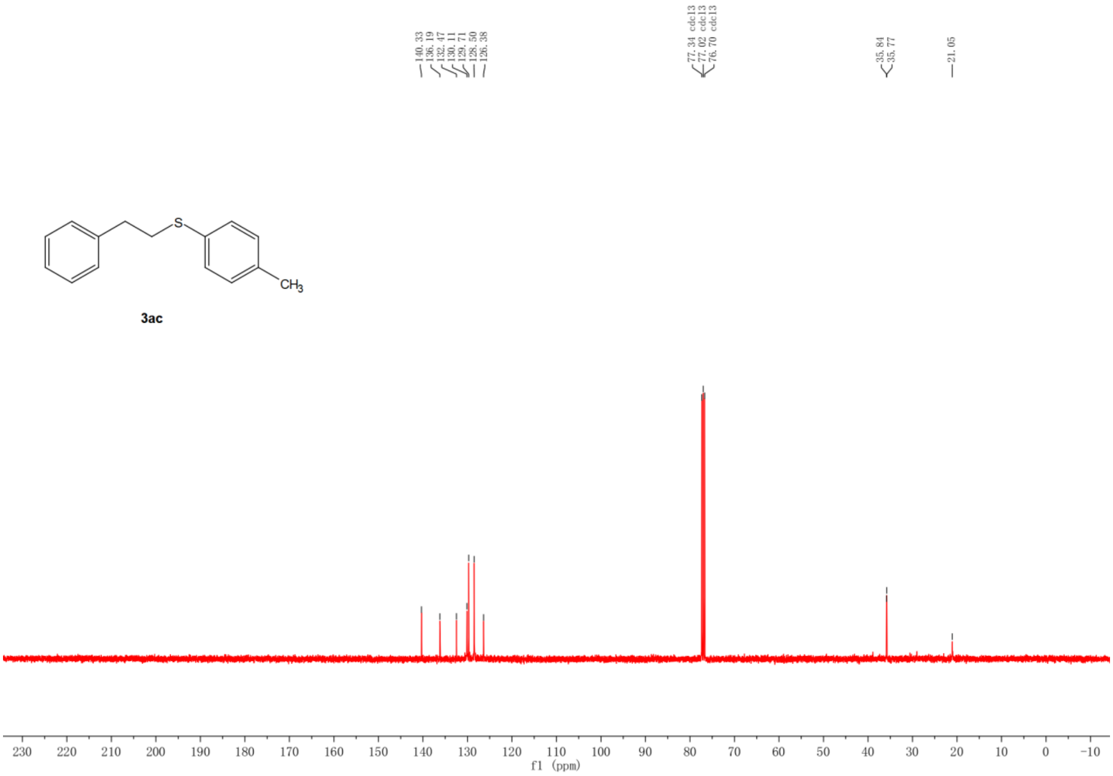

Figure S23. ^{13}C NMR spectra (400 MHz) of **3ac** in $CDCl_3$.

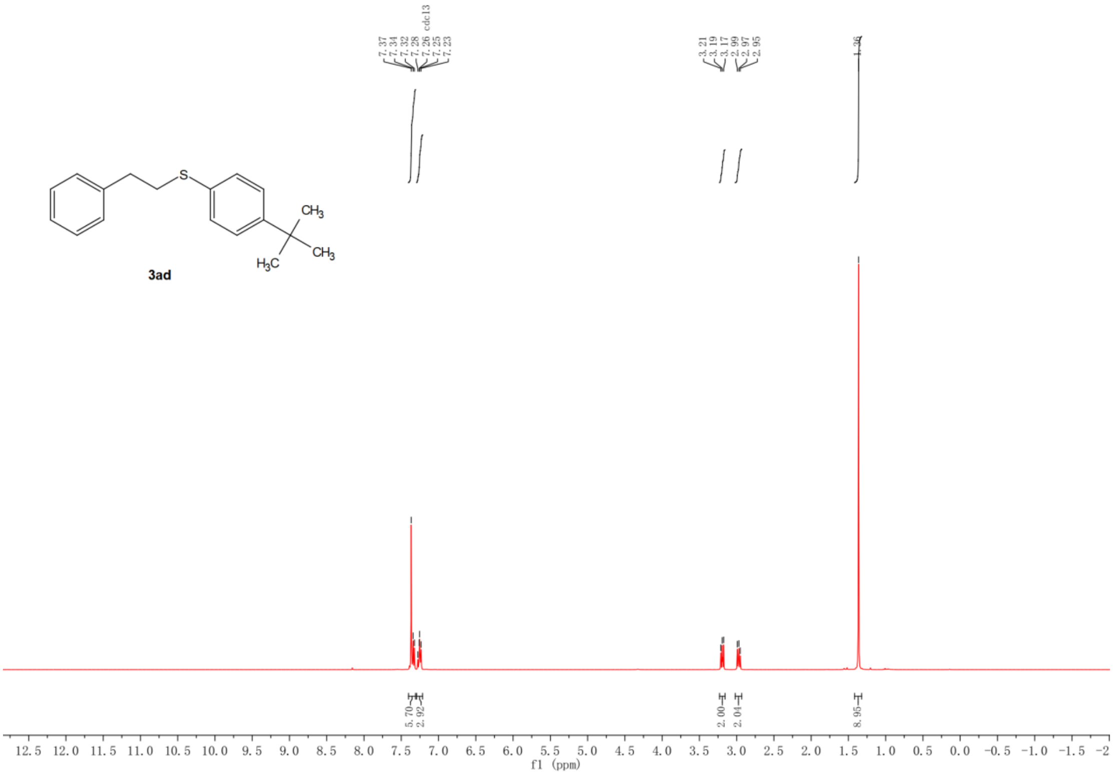

Figure S24. ^{1}H NMR spectra (400 MHz) of **3ad** in $CDCl_3$.

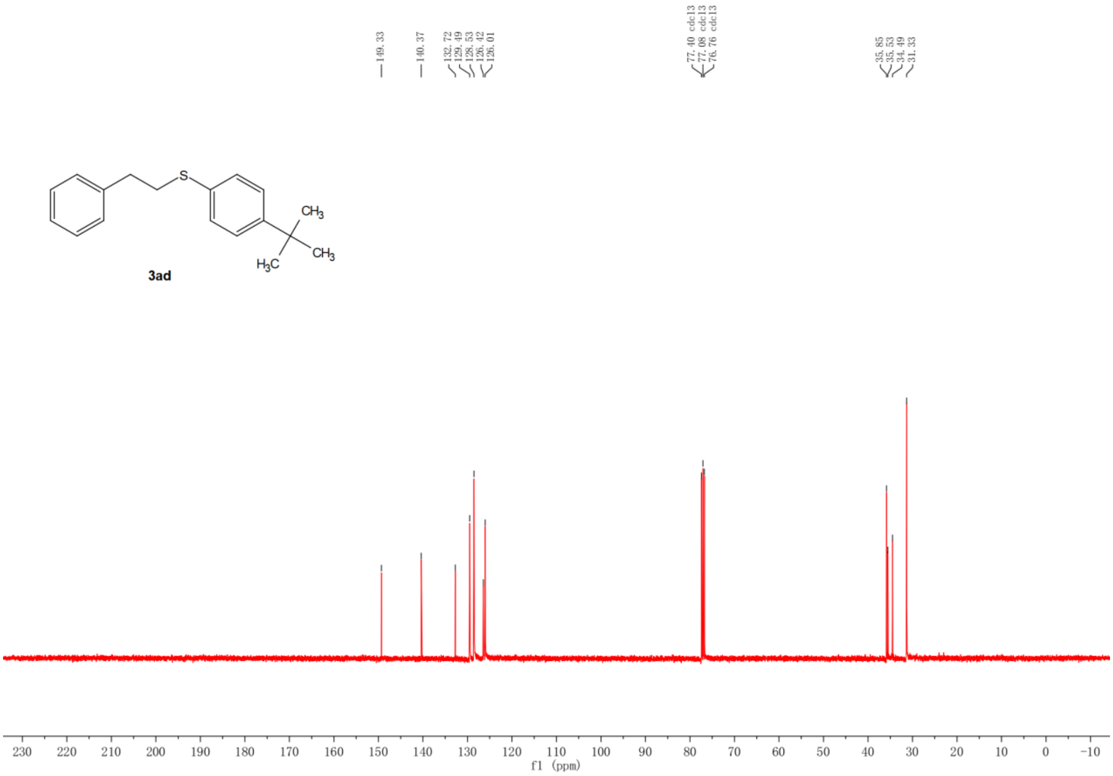

Figure S25. ^{13}C NMR spectra (400 MHz) of **3ad** in $CDCl_3$.

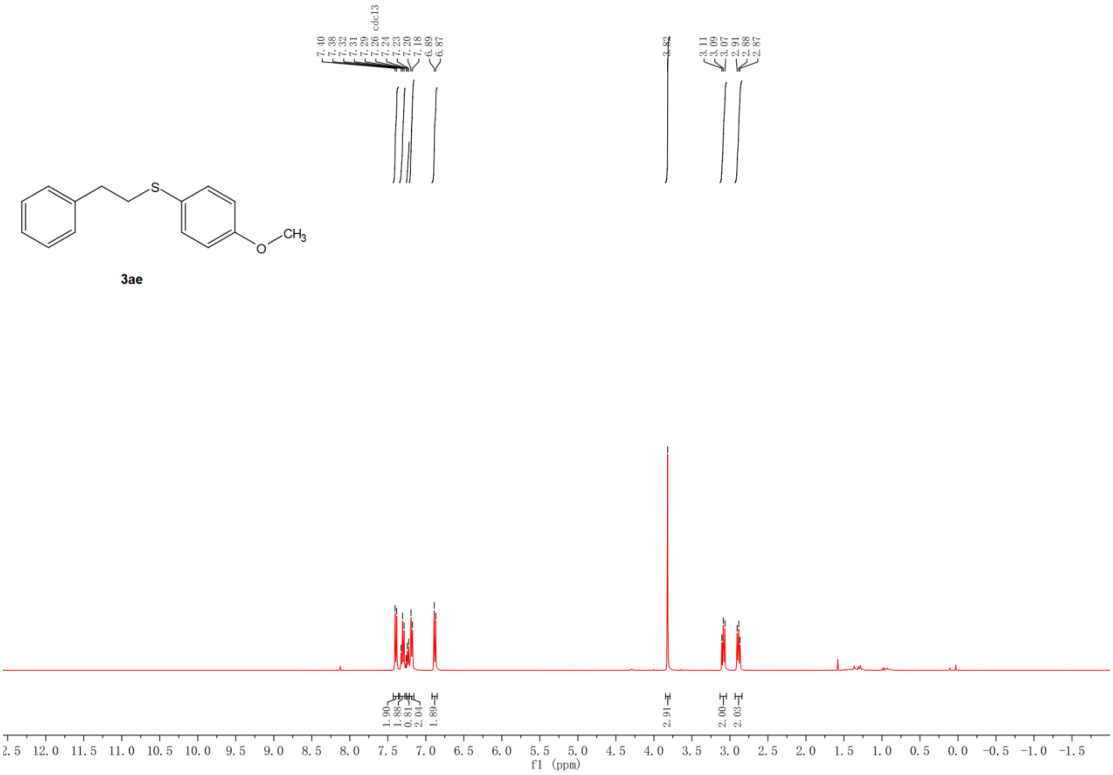

Figure S26. ^{1}H NMR spectra (400 MHz) of **3ae** in $CDCl_3$.

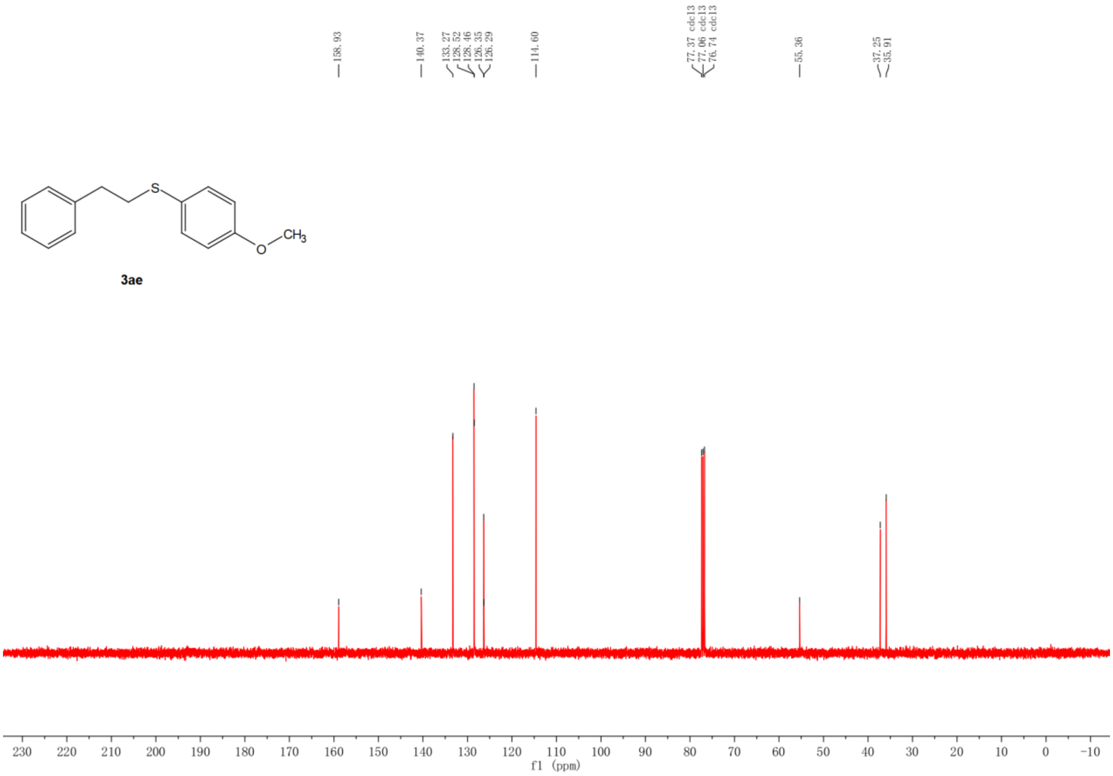

Figure S27. ^{13}C NMR spectra (400 MHz) of **3ae** in $CDCl_3$.

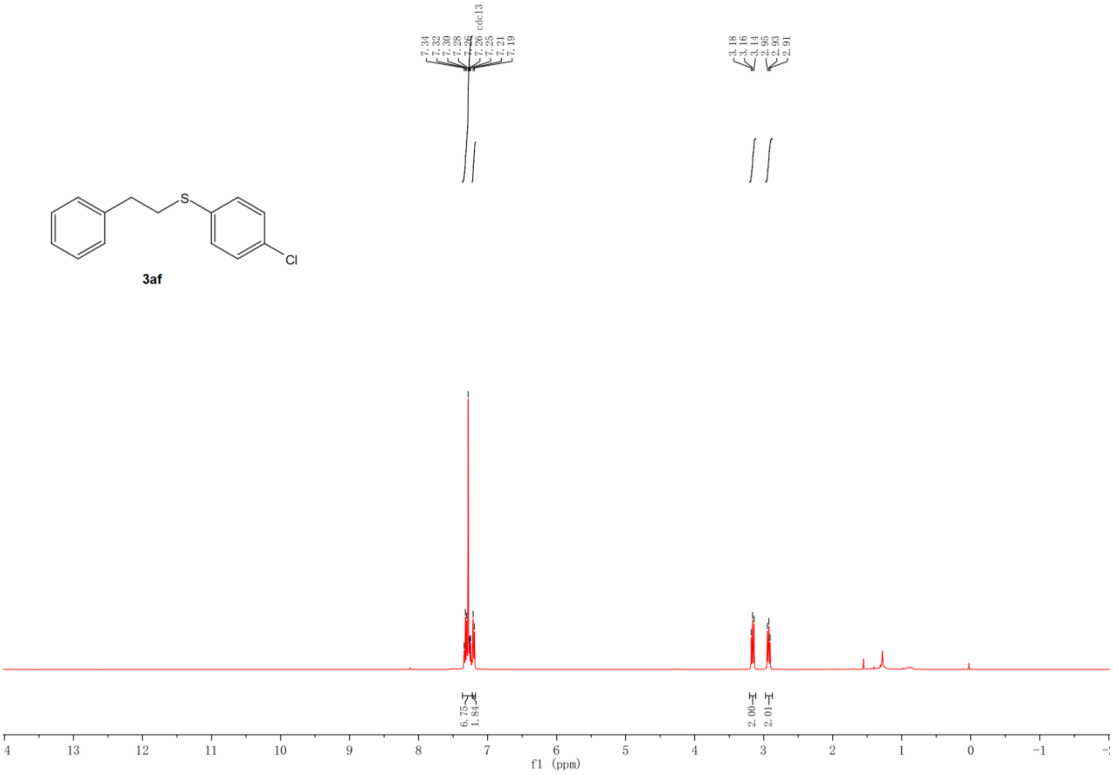

Figure S28. ^{1}H NMR spectra (400 MHz) of **3af** in $CDCl_3$.

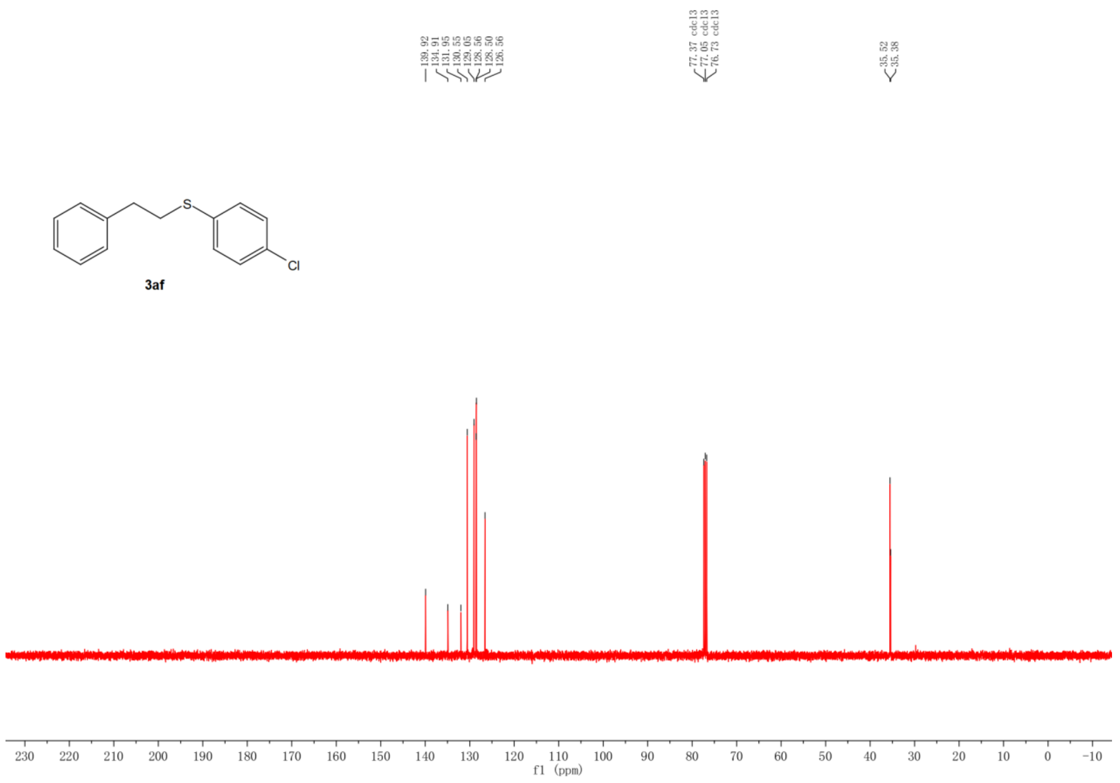

Figure S29. ^{13}C NMR spectra (400 MHz) of **3af** in $CDCl_3$.

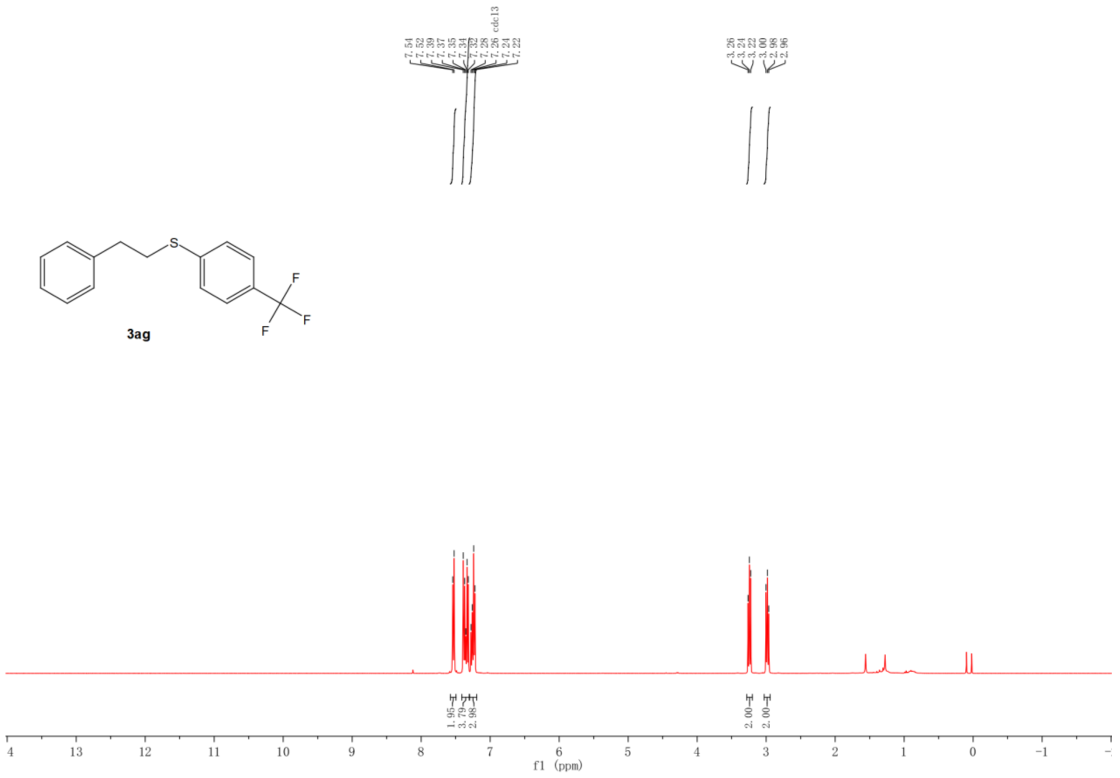

Figure S30. ^{1}H NMR spectra (400 MHz) of **3ag** in $CDCl_3$.

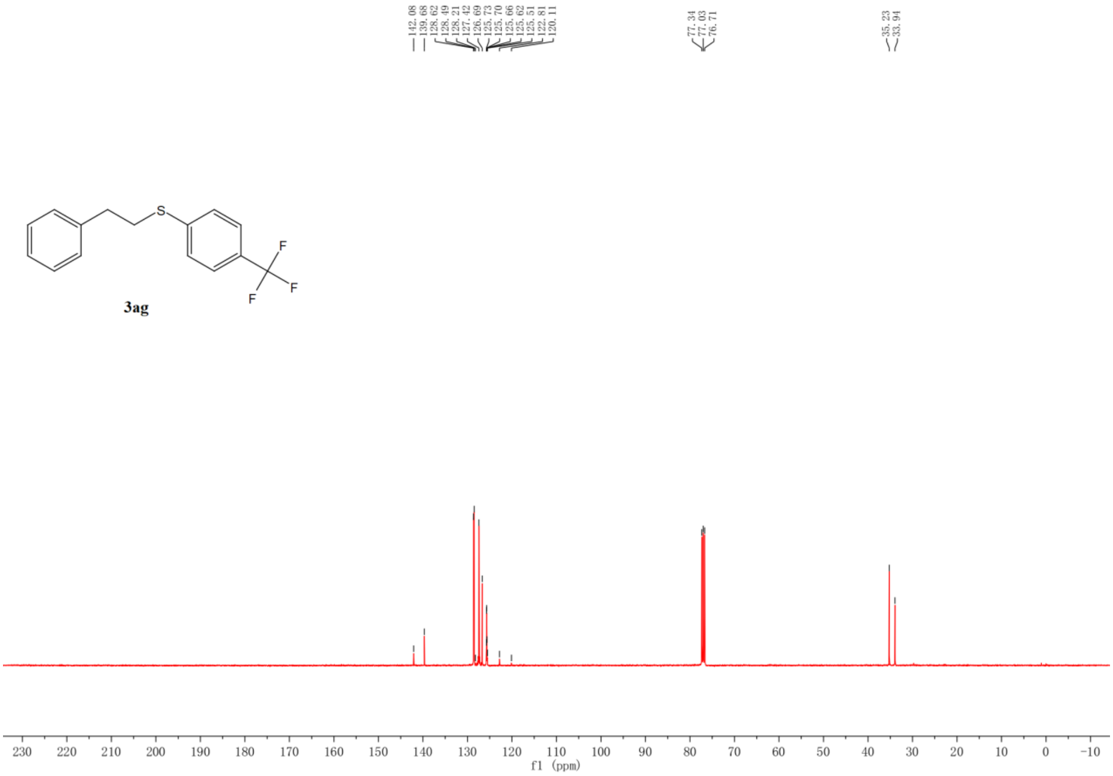

Figure S31. ^{13}C NMR spectra (400 MHz) of **3ag** in $CDCl_3$.

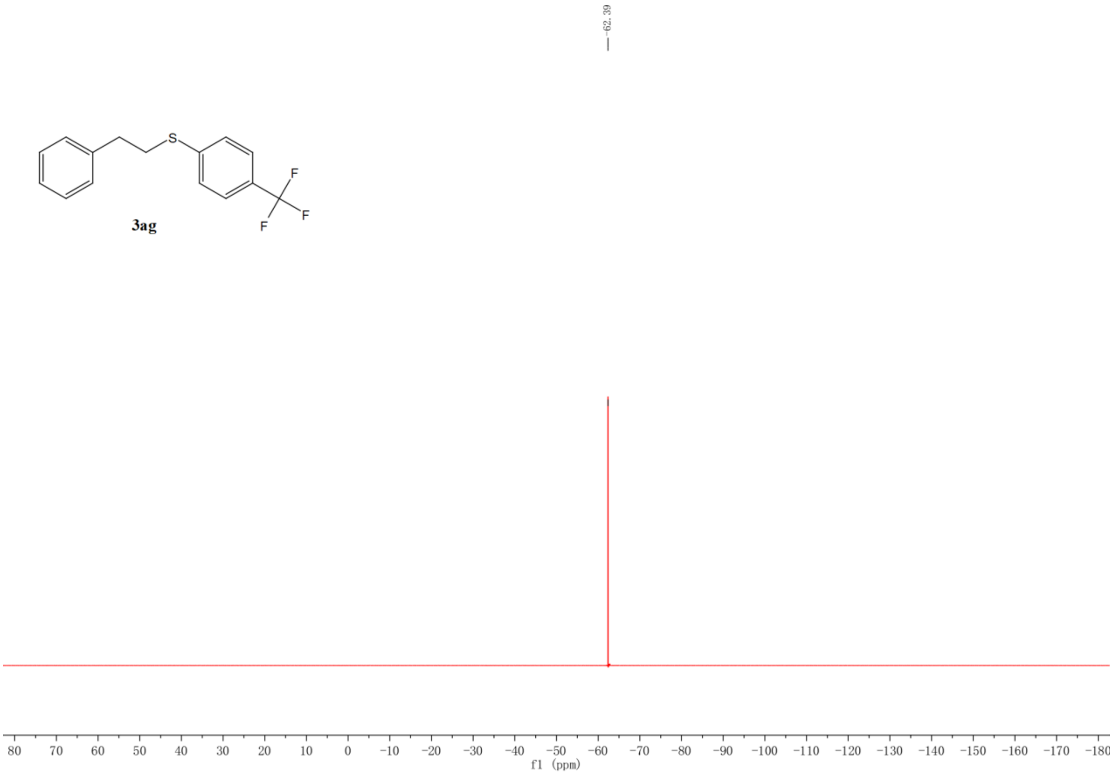

Figure S32. ^{19}F NMR spectra (400 MHz) of **3ag** in $CDCl_3$.

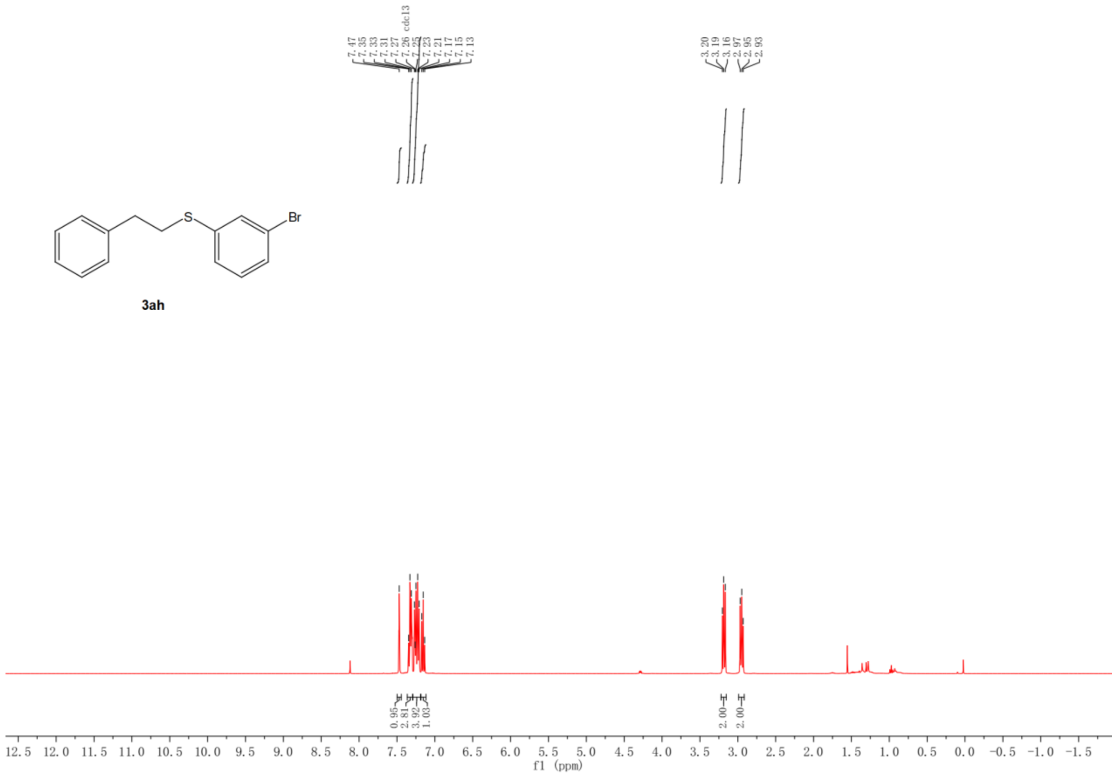

Figure S33. ^{1}H NMR spectra (400 MHz) of **3ah** in $CDCl_3$.

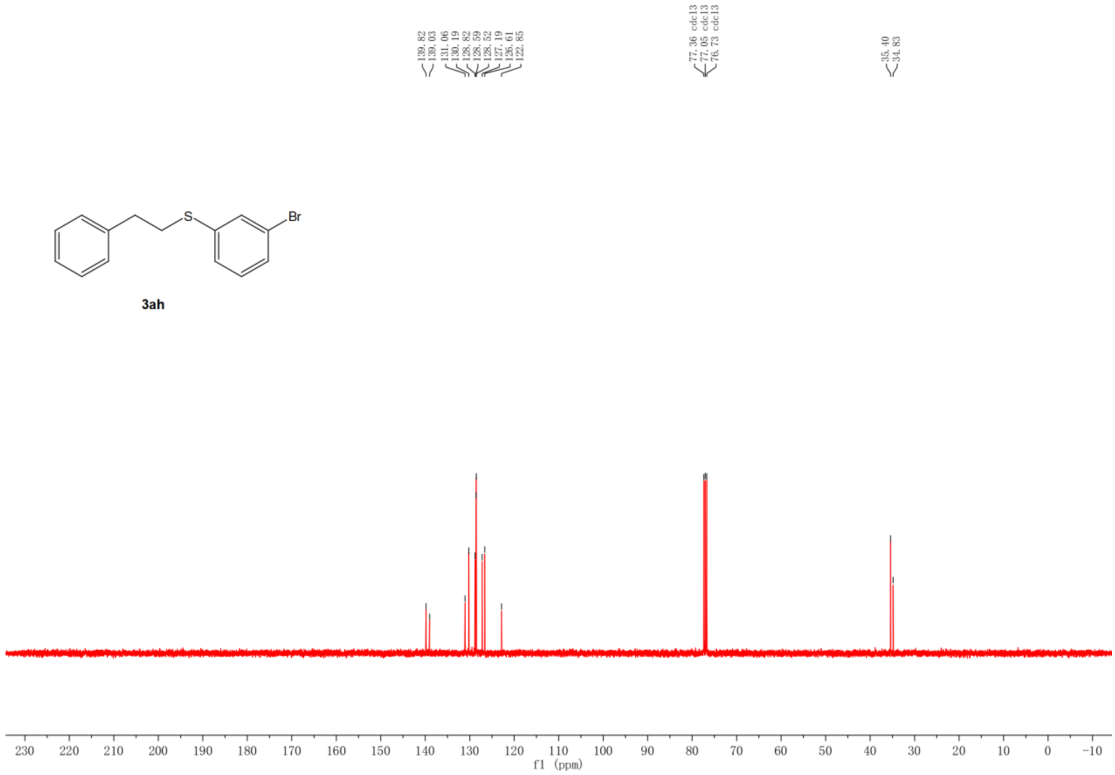

Figure S34. ^{13}C NMR spectra (400 MHz) of **3ah** in $CDCl_3$.

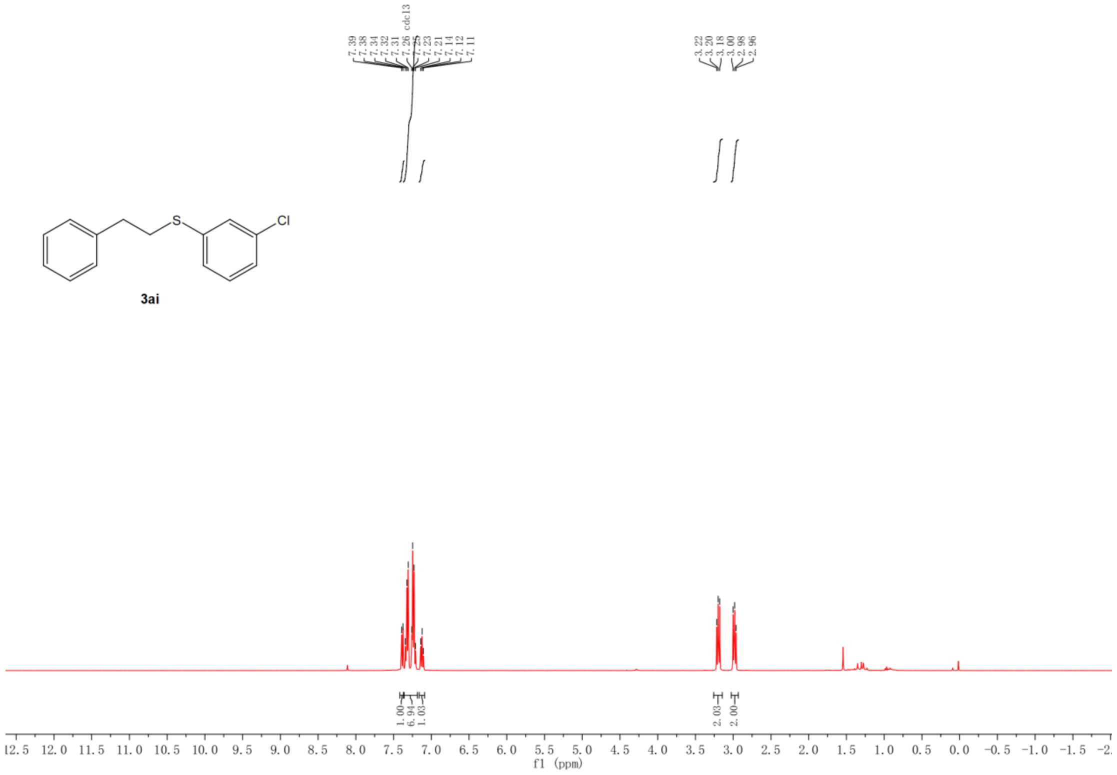

Figure S35. ^{1}H NMR spectra (400 MHz) of **3ai** in $CDCl_3$.

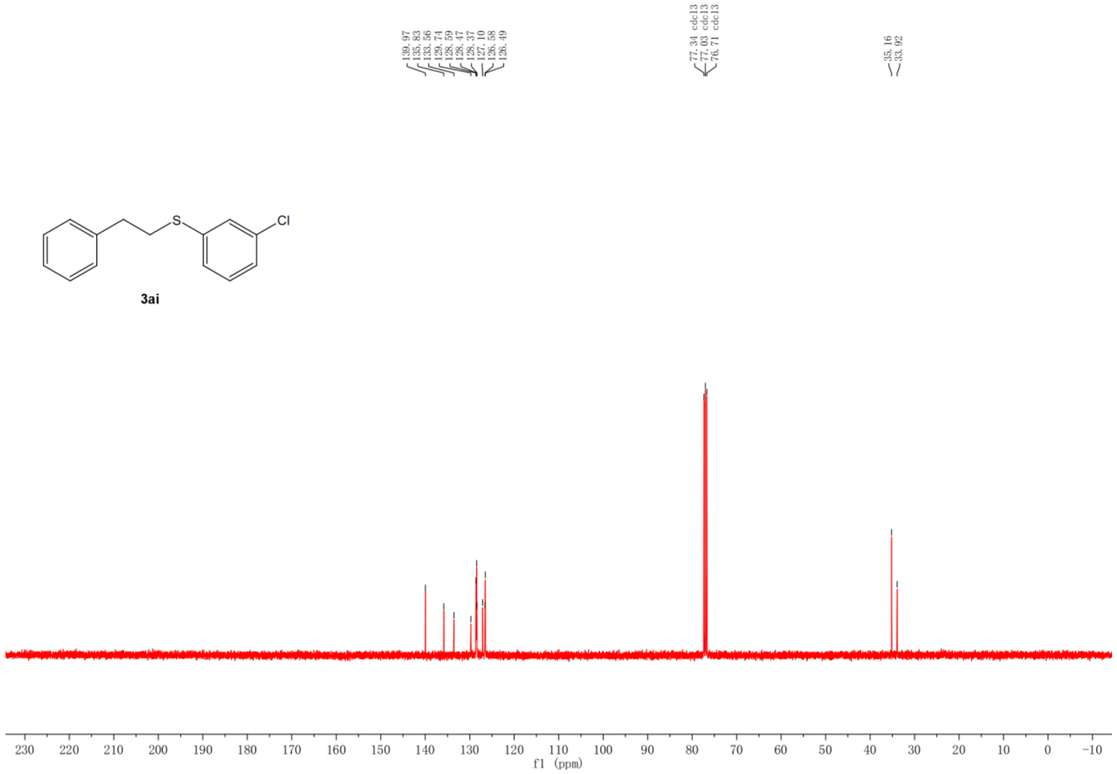

Figure S36. ^{13}C NMR spectra (400 MHz) of **3ai** in $CDCl_3$.

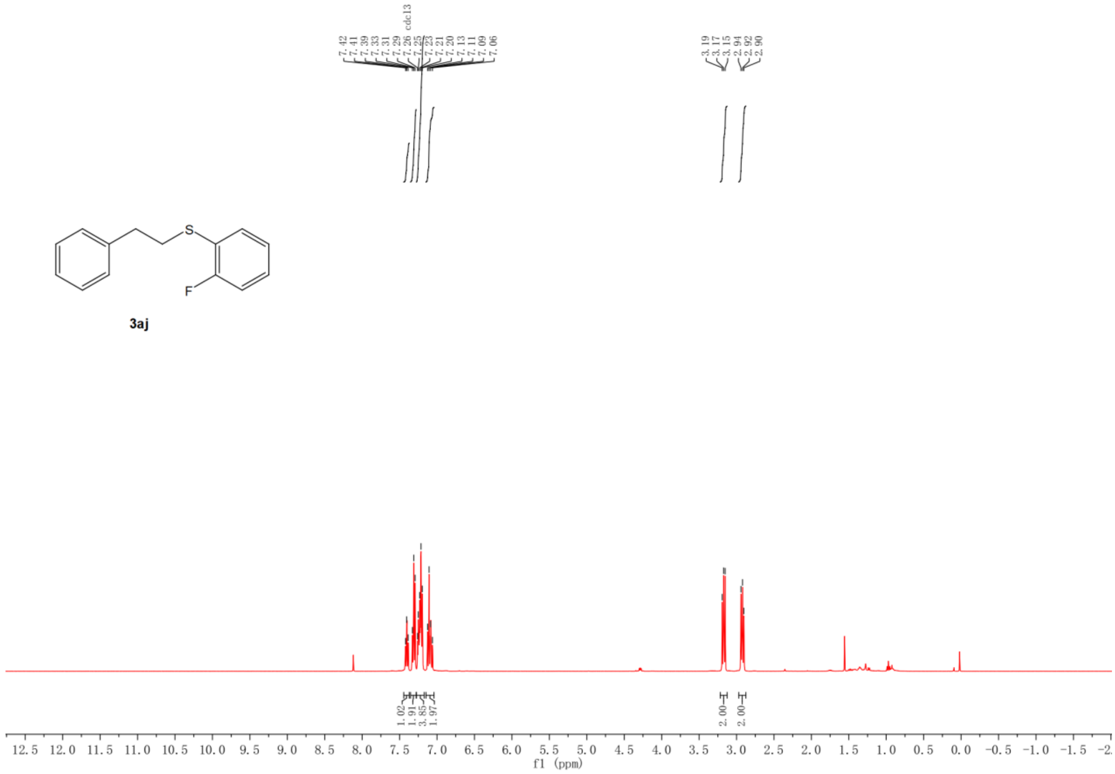

Figure S37. ^{1}H NMR spectra (400 MHz) of **3aj** in $CDCl_3$.

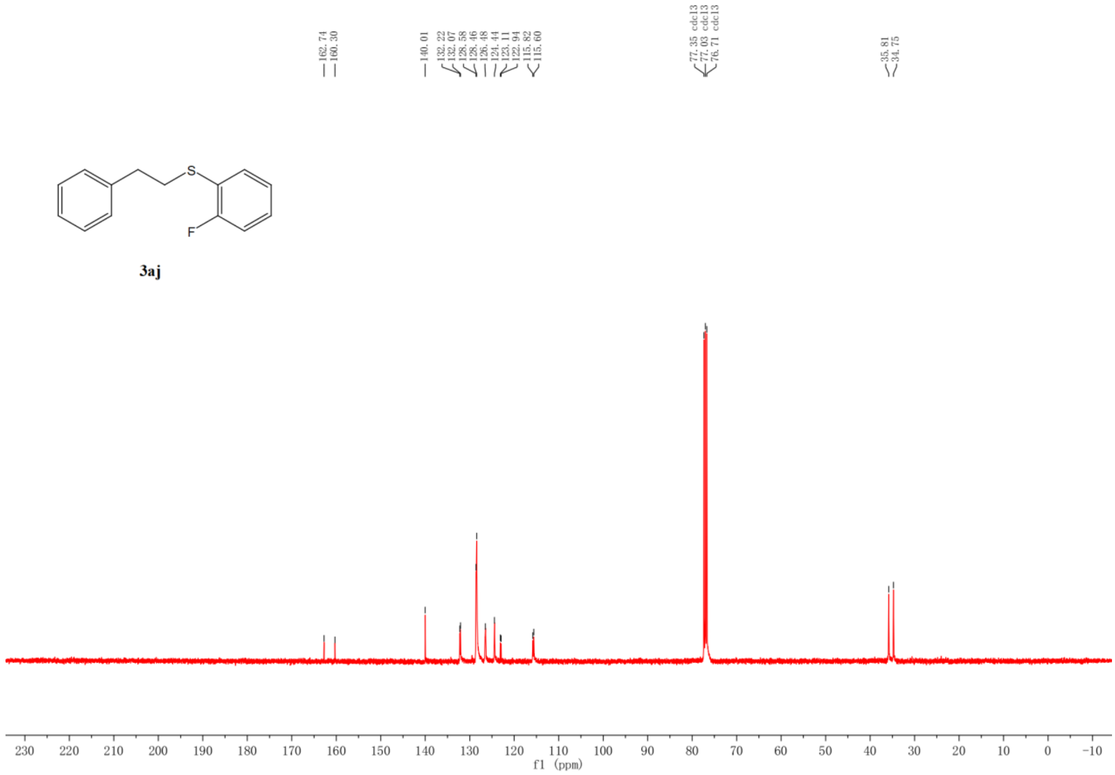

Figure S38. ^{13}C NMR spectra (400 MHz) of **3aj** in $CDCl_3$.

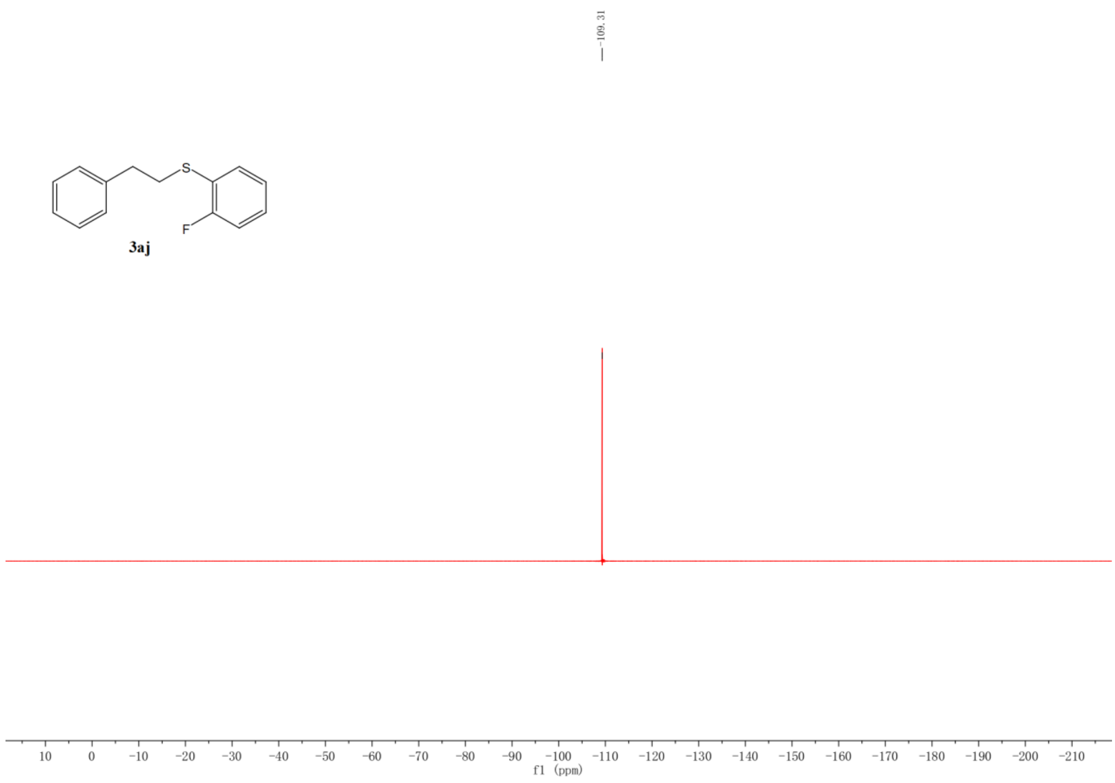

Figure S39. ^{19}F NMR spectra (400 MHz) of **3aj** in $CDCl_3$.

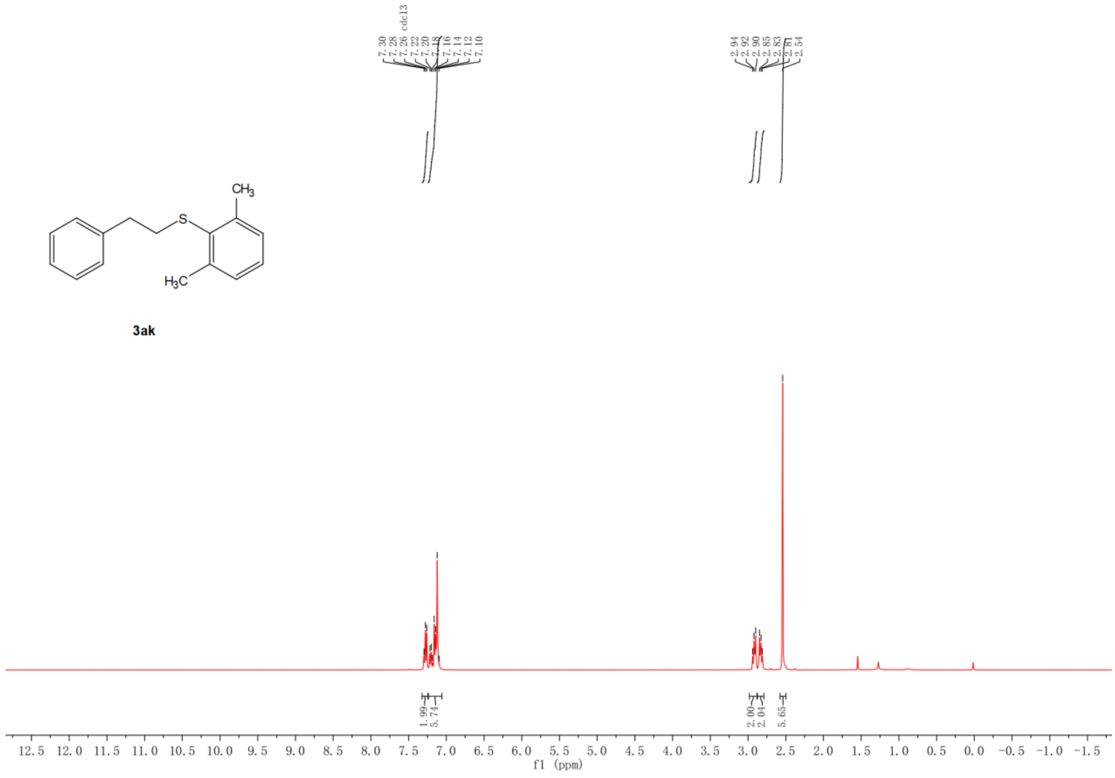

Figure S40. ^{1}H NMR spectra (400 MHz) of **3ak** in $CDCl_3$.

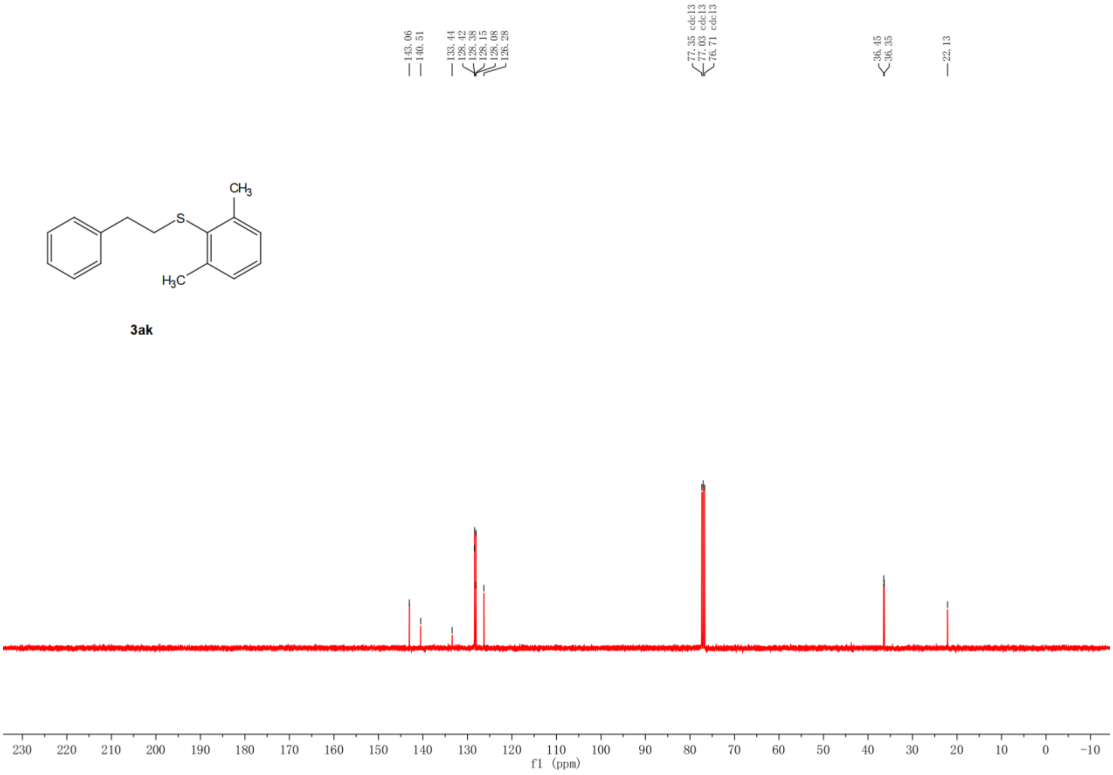

Figure S41. ^{13}C NMR spectra (400 MHz) of **3ak** in $CDCl_3$.

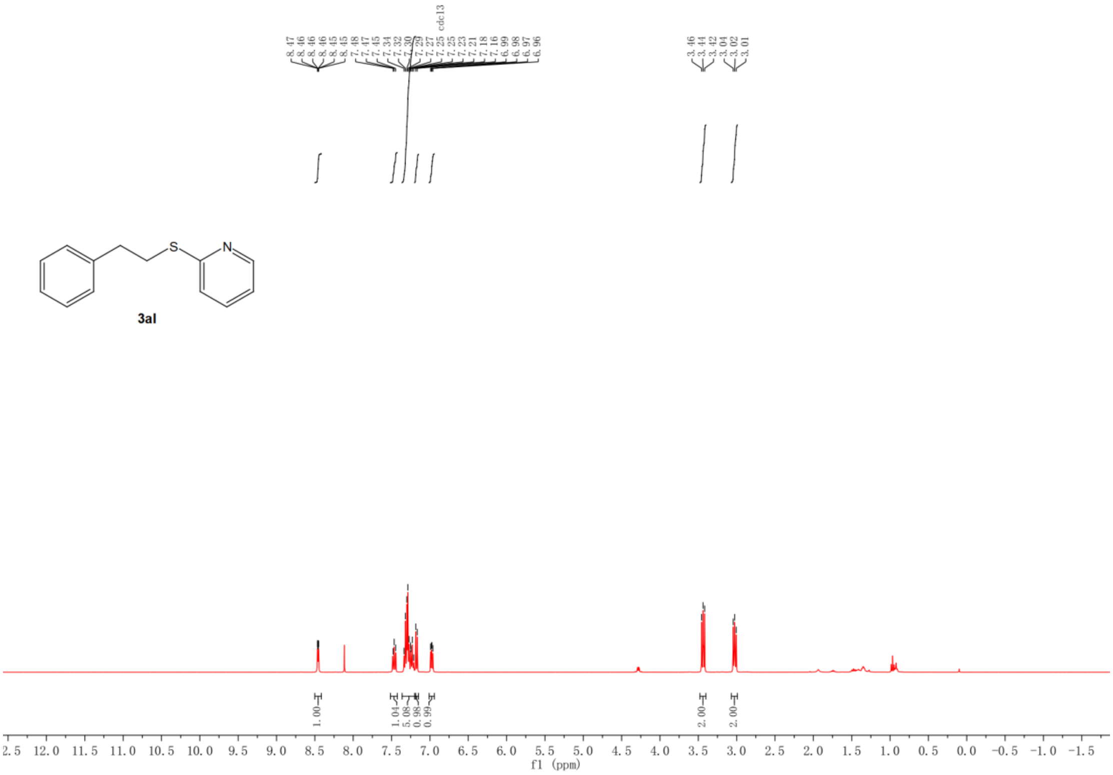

Figure S42. ^{1}H NMR spectra (400 MHz) of **3al** in $CDCl_3$.

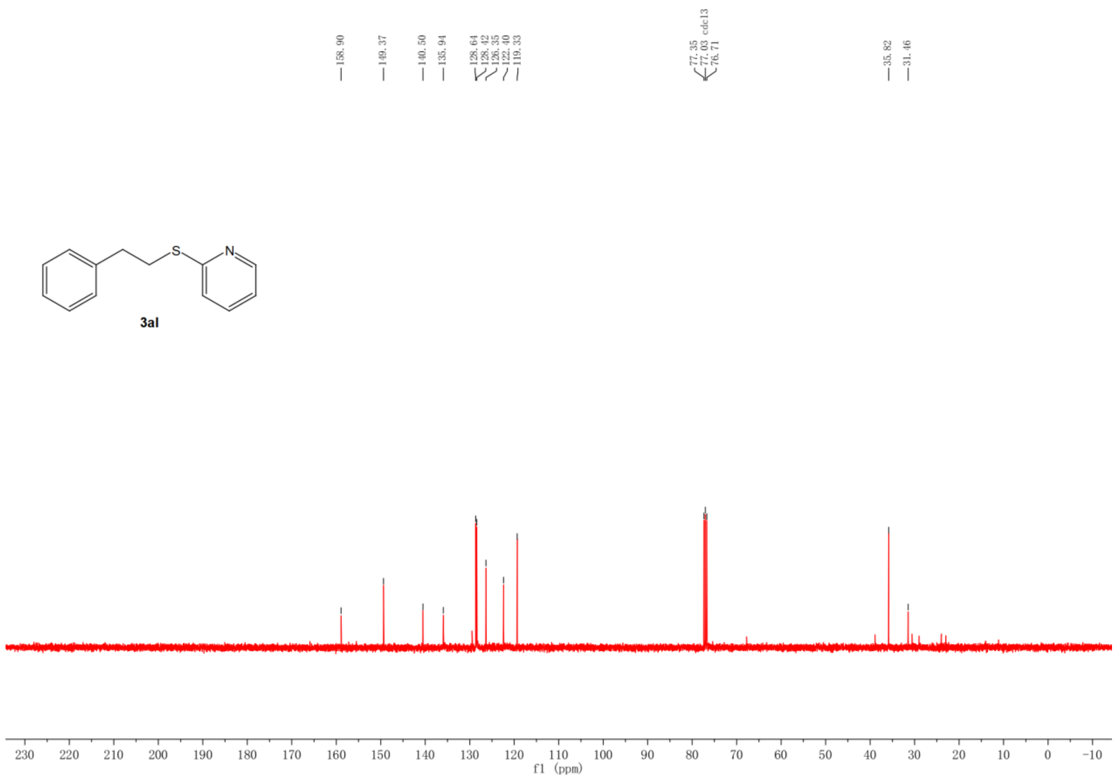

Figure S43. ^{13}C NMR spectra (400 MHz) of **3al** in $CDCl_3$.

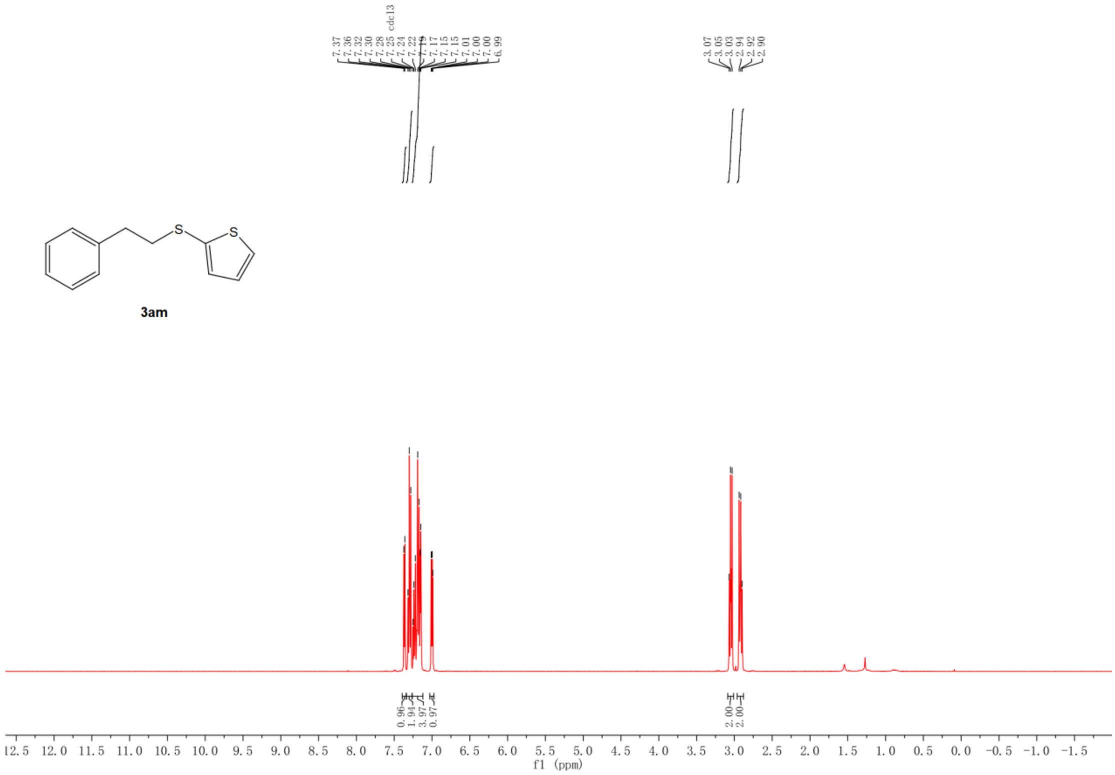

Figure S44. ^{1}H NMR spectra (400 MHz) of **3am** in $CDCl_3$.

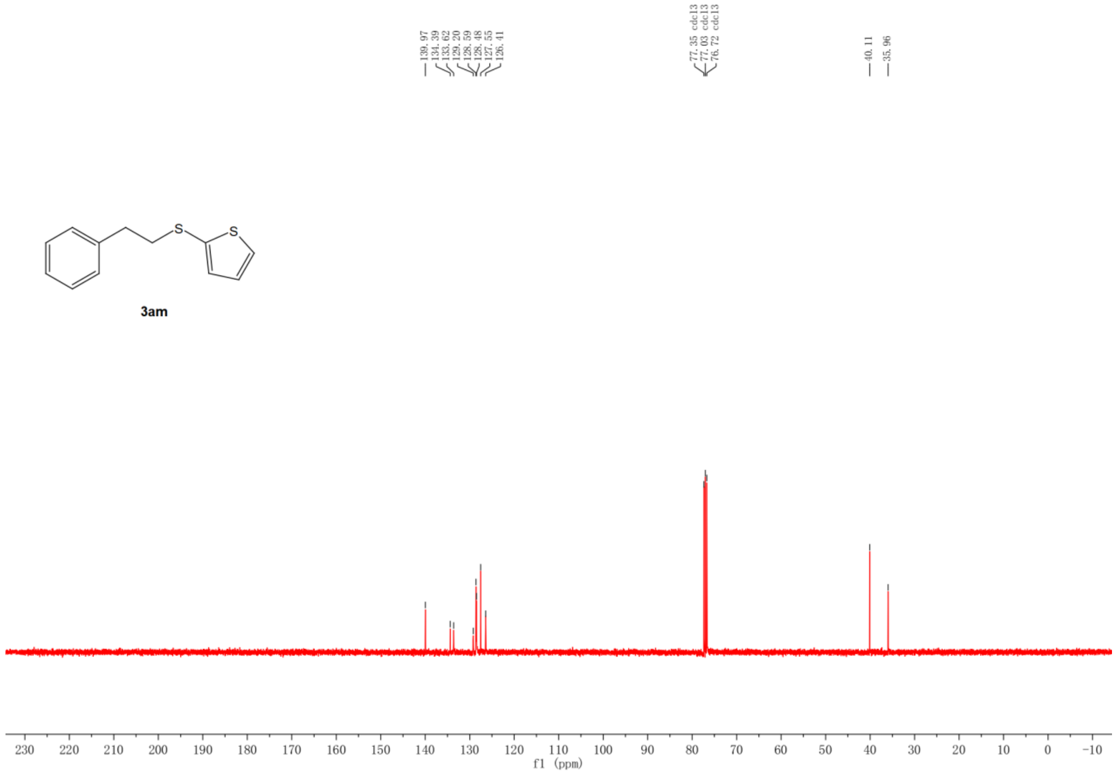

Figure S45. ^{13}C NMR spectra (400 MHz) of **3am** in $CDCl_3$.

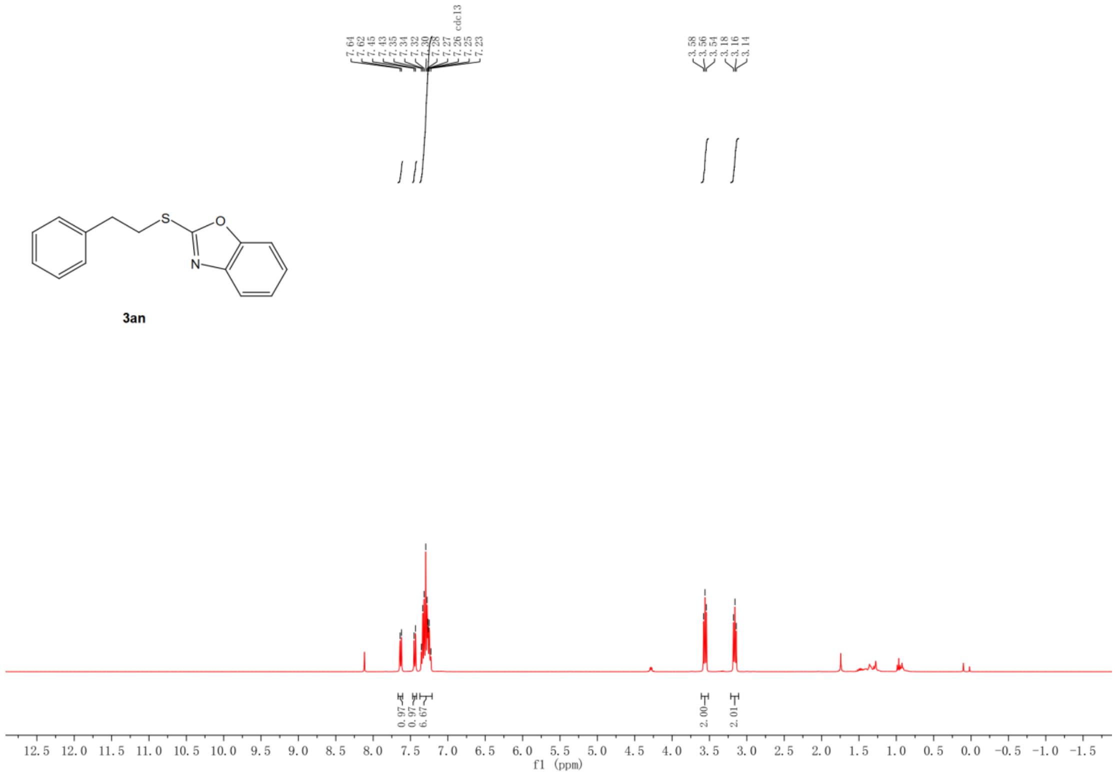

Figure S46. ^{1}H NMR spectra (400 MHz) of **3an** in $CDCl_3$.

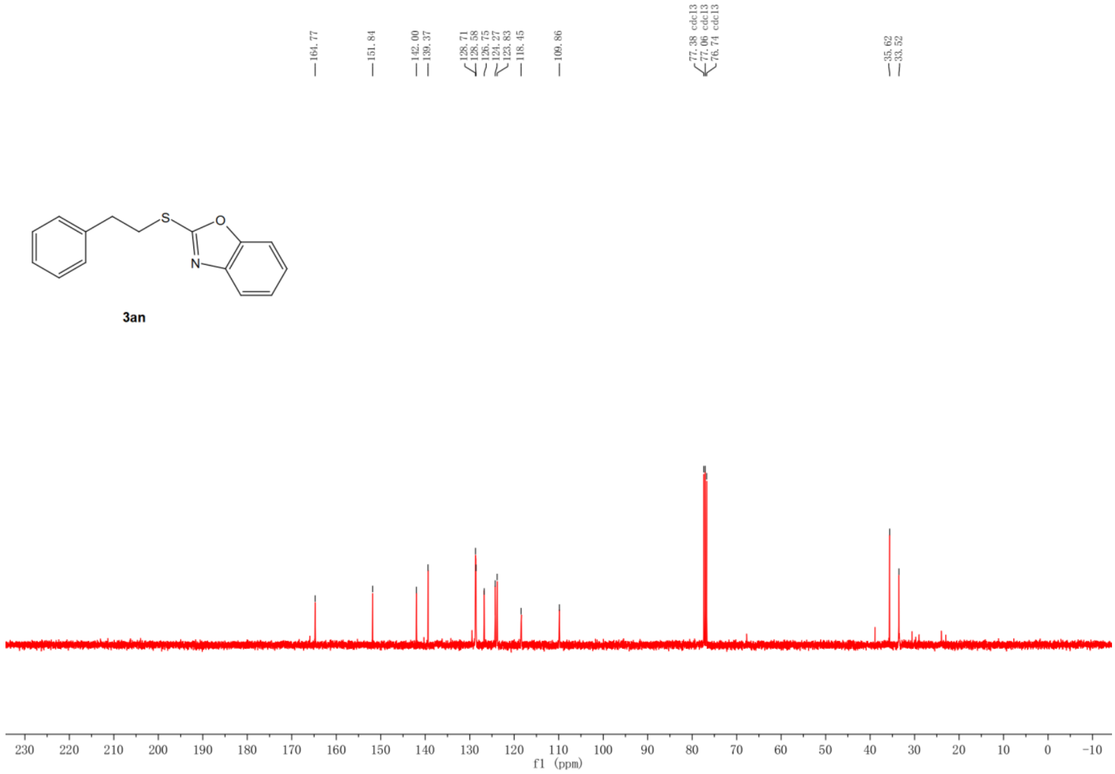

Figure S47. ^{13}C NMR spectra (400 MHz) of **3an** in $CDCl_3$.

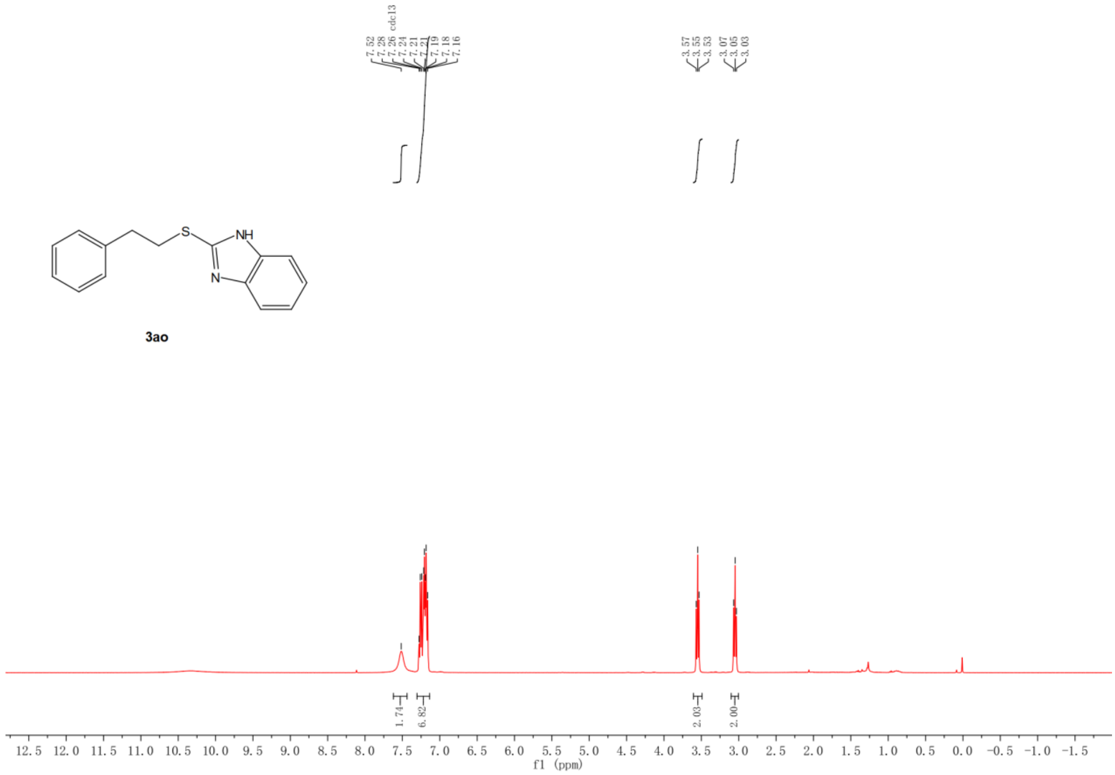

Figure S48. ^{1}H NMR spectra (400 MHz) of **3ao** in $CDCl_3$.

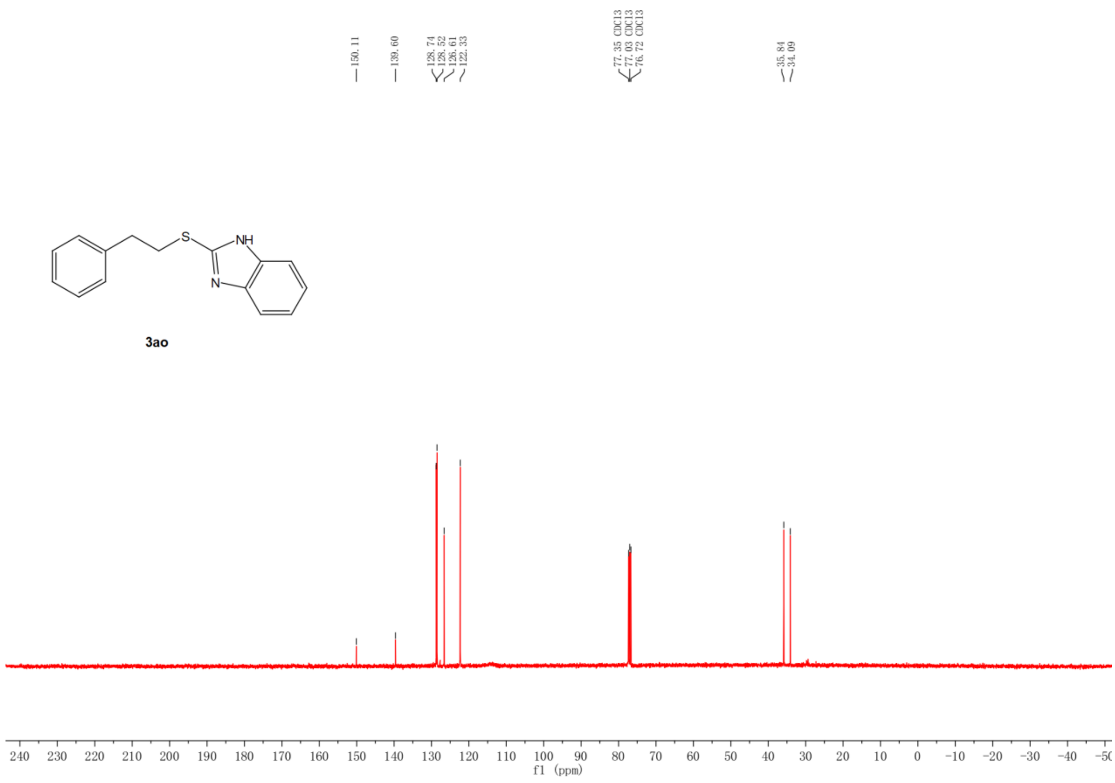

Figure S49. ^{13}C NMR spectra (400 MHz) of **3ao** in $CDCl_3$.

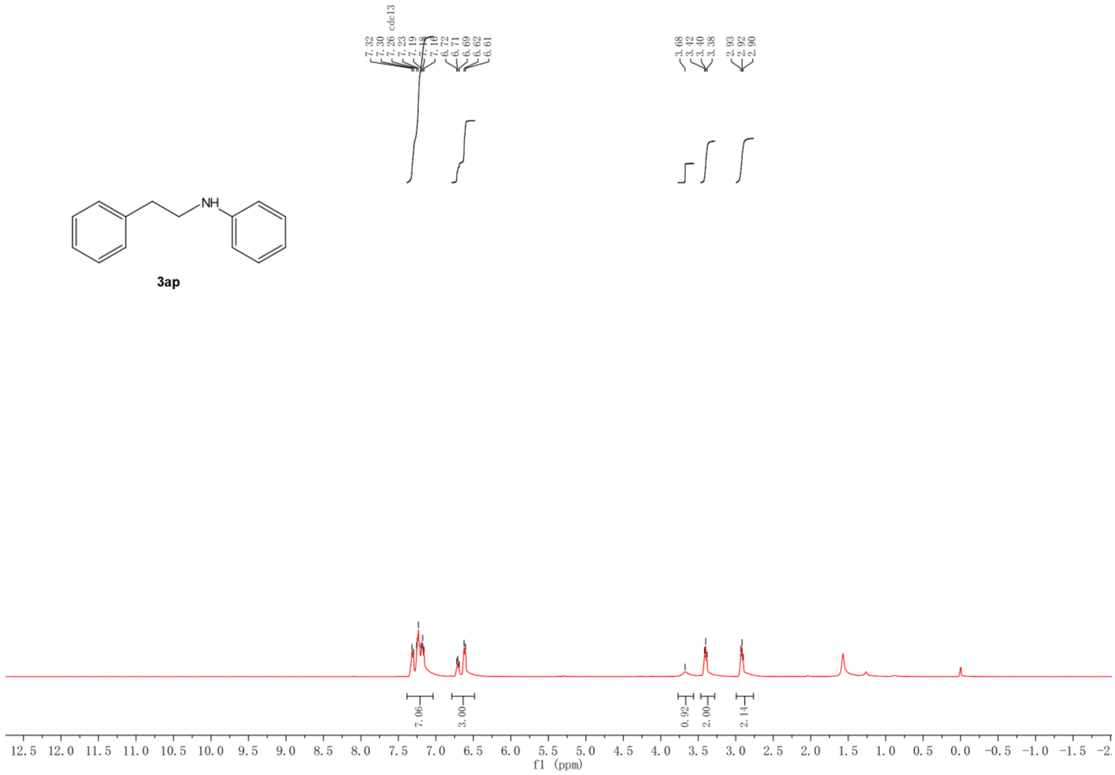

Figure S50. ^{1}H NMR spectra (400 MHz) of **3ap** in $CDCl_3$.

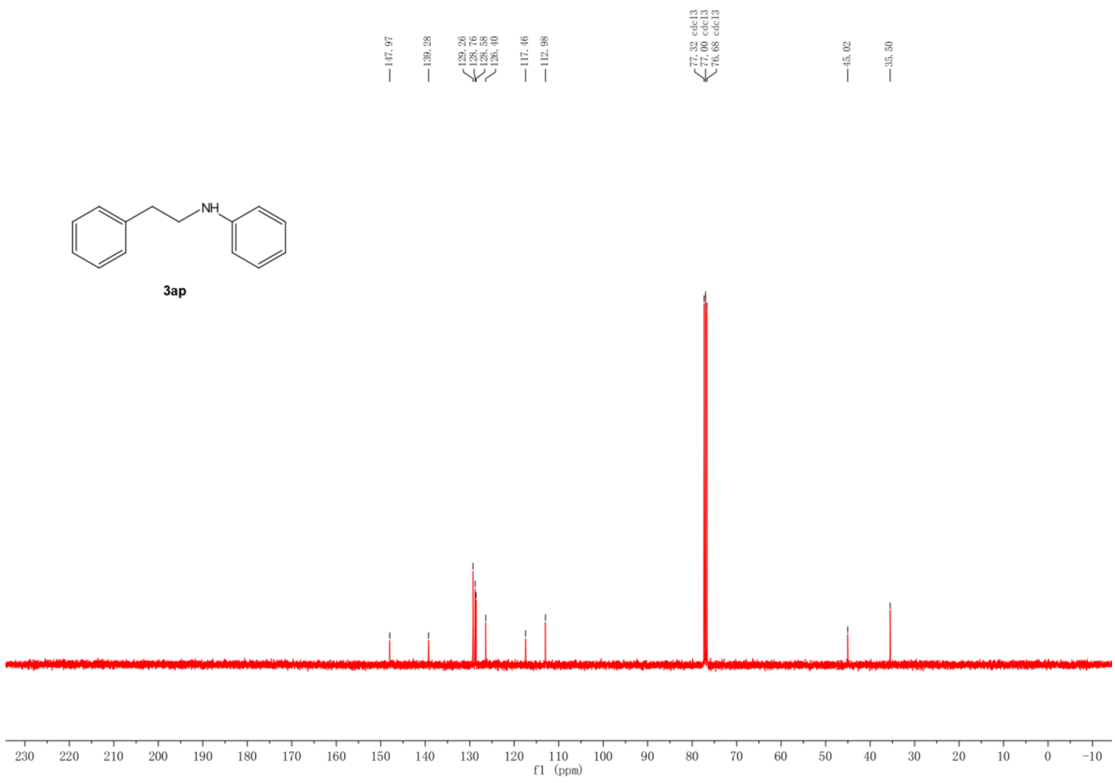

Figure S51. ^{13}C NMR spectra (400 MHz) of **3ap** in $CDCl_3$.

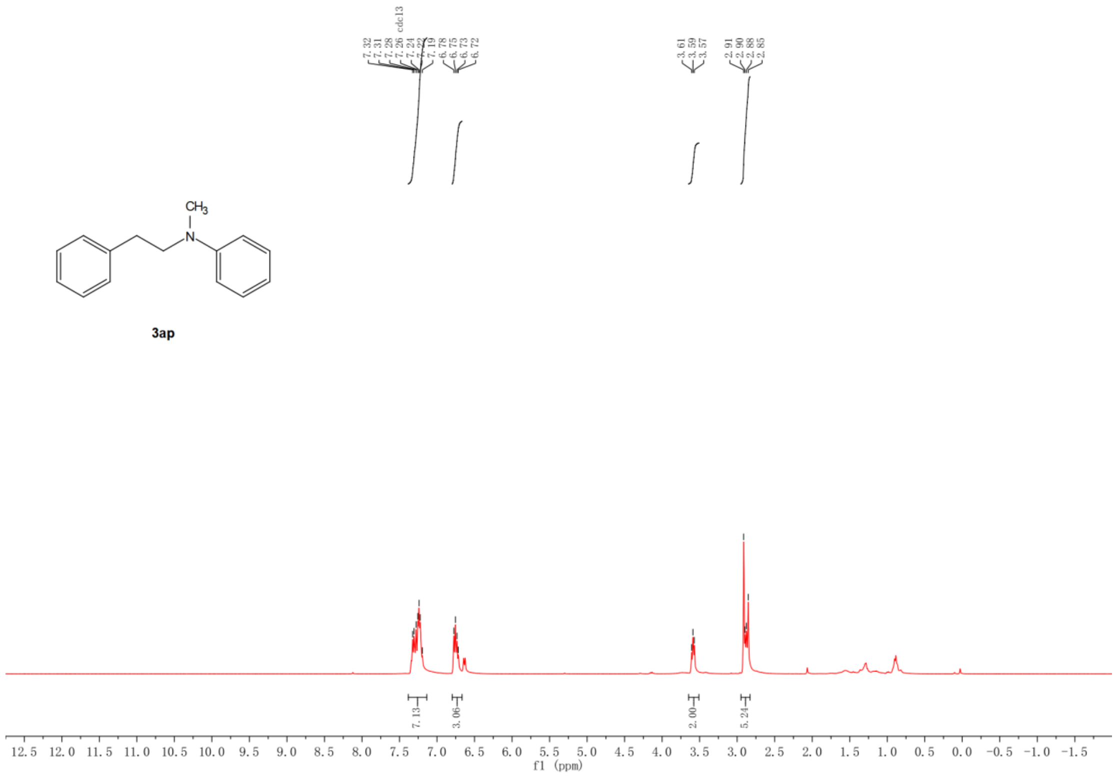

Figure S52. ^{1}H NMR spectra (400 MHz) of **3aq** in $CDCl_3$.

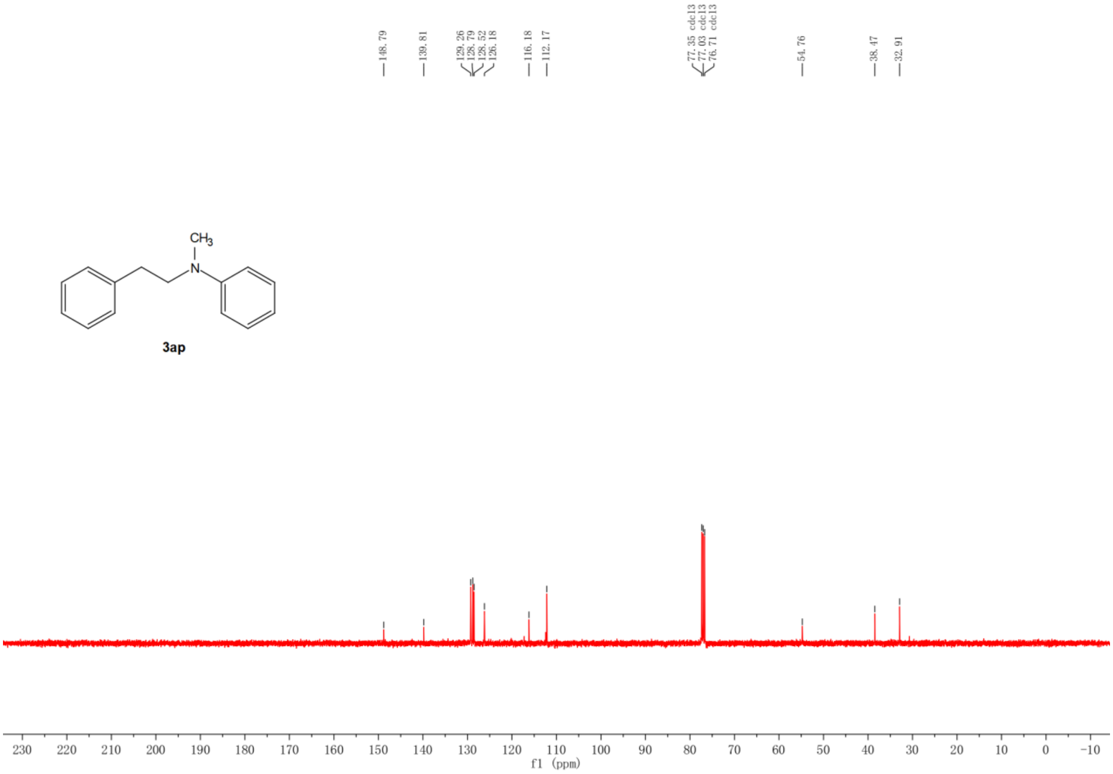

Figure S53. ^{13}C NMR spectra (400 MHz) of **3aq** in $CDCl_3$.

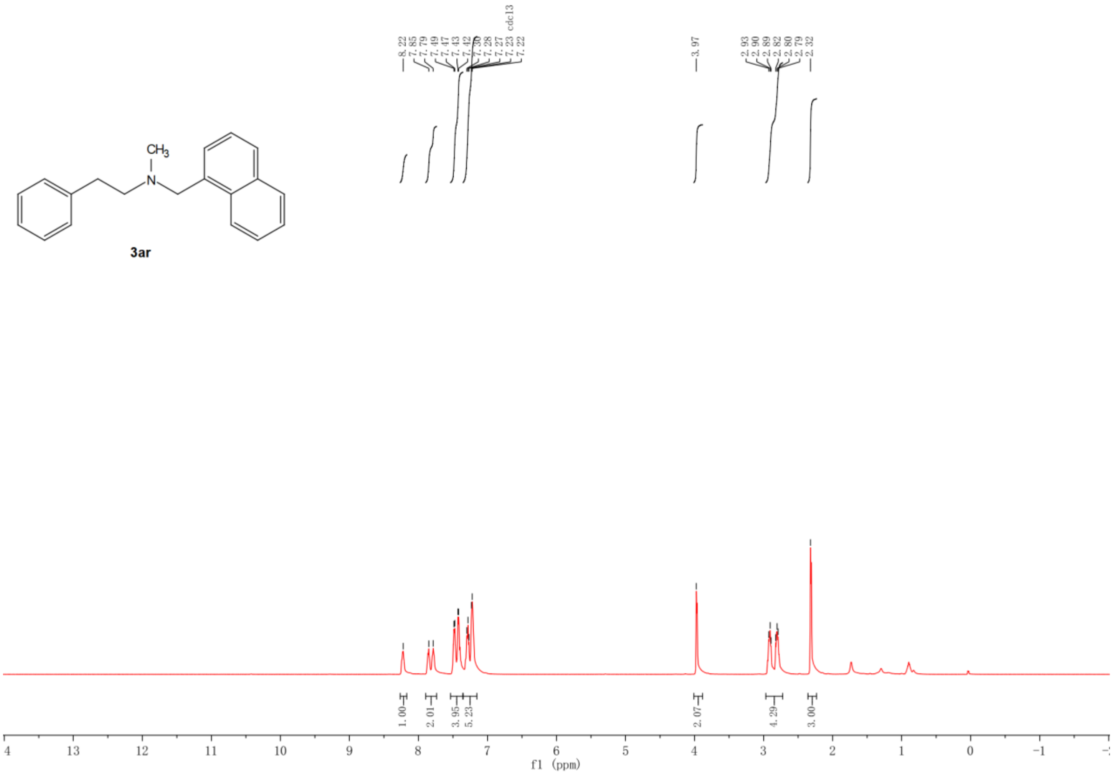

Figure S54. ^{1}H NMR spectra (400 MHz) of **3ar** in $CDCl_3$.

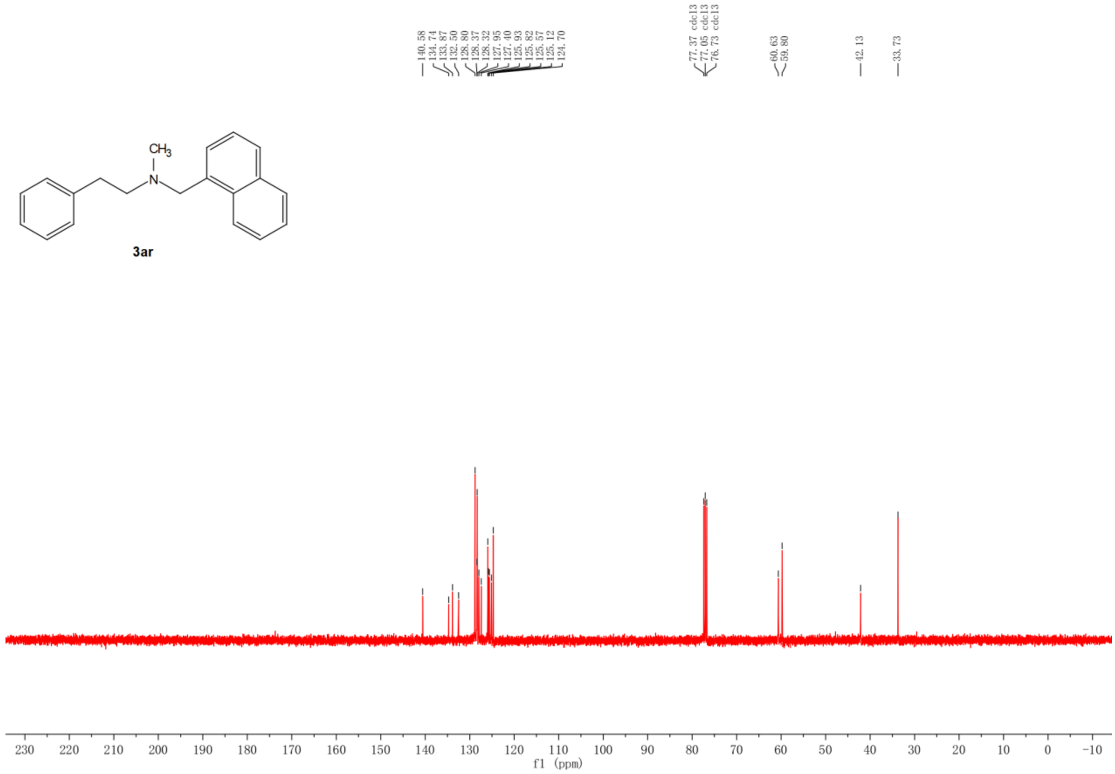

Figure S55. ^{13}C NMR spectra (400 MHz) of **3ar** in $CDCl_3$.
